# *Klebsiella pneumoniae* bacteremia mortality: a systematic review and meta-analysis

**DOI:** 10.3389/fcimb.2023.1157010

**Published:** 2023-04-20

**Authors:** Dan Li, Xiangning Huang, Huayun Rao, Hua Yu, Shanshan Long, Yulian Li, Jie Zhang

**Affiliations:** ^1^ School of Medicine, University of Electronic Science and Technology of China, Chengdu, Sichuan, China; ^2^ Department of Laboratory Medicine, Sichuan Provincial People’s Hospital, University of Electronic Science and Technology of China, Chengdu, Sichuan, China; ^3^ Department of Laboratory Medicine, Medical Center Hospital of Qionglai City, Chengdu, Sichuan, China; ^4^ College of Medical Technology, Chengdu University of Traditional Chinese Medicine, Chengdu, China

**Keywords:** *Klebsiella pneumoniae*, bacteremia, mortality, meta-analysis, carbapenem-resistant

## Abstract

**Objective:**

To analyze the mortality rate of patients with *Klebsiella pneumoniae* bacteremia (KPB) and the impact of extended spectrum beta-lactamase (ESBL) producing or carbapenem-resistance (CR) KP on the mortality rate among patients with bacteremia.

**Methods:**

EMbase, Web of Science, PubMed, and The Cochrane Library were searched up to September 18^th^, 2022. Two reviewers independently extracted data and evaluated risk of bias of included studies by ROBINS-I tool. A meta-regression analysis was conducted using a mixed-effects model to explore possible sources of heterogeneity. A random-effects model was used for pooled analysis in case of significant heterogeneity (I^2^>50%). Otherwise, the fixed-effects model was performed.

**Results:**

A total of 157 studies (37,915 enrolled patients) were included in the meta-analysis. The pooled death proportions of KPB were 17% (95% CI=0.14-0.20) at 7-day, 24% (95% CI=0.21-0.28) at 14-day, 29% (95% CI=0.26-0.31) at 30-day, 34% (95% CI=0.26-0.42) at 90-day, and 29% (95% CI=0.26-0.33) in hospital, respectively. Heterogeneity was found from the intensive care unit (ICU), hospital-acquired (HA), CRKP, and ESBL-KP in the meta-regression analysis. More than 50% of ICU, HA, CRKP, and ESBL-KP were associated with a significant higher 30-day mortality rates. The pooled mortality odds ratios (ORs) of CRKP *vs*. non-CRKP were 3.22 (95% CI 1.18-8.76) at 7-day, 5.66 (95% CI 4.31-7.42) at 14-day, 3.87 (95% CI 3.01-3.49) at 28- or 30-day, and 4.05 (95% CI 3.38-4.85) in hospital, respectively.

**Conclusions:**

This meta-analysis indicated that patients with KPB in ICU, HA-KPB, CRKP, and ESBL-KP bacteremia were associated with a higher mortality rate. The high mortality rate caused by CRKP bacteremia has increased over time, challenging the public health.

## Introduction

*Klebsiella pneumoniae* (KP) is well known as an opportunistic pathogen which can cause invasive human infections such as bacteremia. Also, KP is the second most common cause of gram-negative bacteremia, following *Escherichia coli* (E. coli) (Cubero et al., 2018; Hyun et al., 2018). The estimated incidence rate of *Klebsiella pneumoniae* bacteremia (KPB) was increased from 10.2 to 18.7 per 100 000 inhabitants in a region of Canada (Pépin et al., 2010), and the reported mortality rate of KPB varied widely from 11% to 81% in most studies (Chang et al., 2015; Imai et al., 2019; Reid et al., 2019; Falcone et al., 2020; Balkan et al., 2021; Meng et al., 2022). It is important to accurately estimate the mortality rate of KPB to best define the infectious disease, and it can accurately convey prognosis and improve the guidance to control this disease.

Antibiotic resistance has become a major challenge for public health globally, which is associated with nearly 5 million deaths and killed at least 1.27 million people worldwide in 2019 alone (CDC, 2022). In addition, the cost to treat infections caused by multidrug-resistant pathogens is high, making a big healthcare burden to society (Zhen et al., 2021). ESBL-producing germs cost the highest for community-onset and hospital-onset infections, with an estimated more than $1.2 billion (Nelson et al., 2021). Antibiotic resistance can develop in KP isolates, especially producing extended-spectrum β-lactamases (ESBL) and carbapenemases. Although many studies (including guidelines) were adopted to maintain the progress in combating antimicrobial resistance, the prevalence of ESBL- and carbapenem-resistant *Klebsiella pneumoniae* (CRKP) has increased dramatically (Gao et al., 2020). According to the 20 Years of the SENTRY Antimicrobial Surveillance Program (Castanheira et al., 2019), a significant increase in CRKP strains was noted over time. Most studies reported patients with ESBL-producing or CRKP bacteremia were associated with higher mortality rate when compared with those who had non-ESBL or non-CRKP bacteremia (Lee et al., 2012; Li et al., 2014; Li and Huang, 2017; Xiao et al., 2018; Lee et al., 2019; Shen et al., 2020; Meng et al., 2022). However, some other researchers reported contrary or similar mortality rates (Lee et al., 2011; Pau et al., 2015; Kim et al., 2019; Lee et al., 2021). Therefore, conducting accurate estimation of the mortality gap between CRKP and non-CRKP bloodstream infections is needed.

Although many individual studies reported the incidence rates of KPB, there is a lack of precise estimations due to limitations, such as biased data collection from single medical center, relatively small sample size, and the different endpoints of the disease. Besides, the impact of ESBL-KP or CRKP on mortality rates among patients with bacteremia should also be studied since 2018 (Ramos-Castaneda et al., 2018). Thus, a systematic review was performed to analyze the mortality rate in KPB as well as the impact of ESBL-KP or CRKP on the mortality rate of patients with bacteremia.

## Methods

### Reporting guideline

The study was reported according to the Preferred Reporting Items for Systematic reviews and Meta-Analyses (PRISMA) guidelines (Page et al., 2021). The PRISMA 2020 checklist is presented in **Supplementary Materials 1** **Table S1**.

### Database search

Two reviewers independently searched EMbase, Web of Science, PubMed, and The Cochrane Library, up to September 18^th^, 2022. The search was performed using both Medical Subject Headings (MeSH) and keywords: “Klebsiella pneumoniae”, “bacteremia or bloodstream infection”, and “mortality or death or survival or outcome” with no date or language restrictions. The search strategy is shown in **Supplementary Material 1 Text S1**. In addition, manual search was performed to find relevant studies from the references of the found studies.

### Study criteria and definitions

Inclusion criteria were as follows: i) Observational studies; ii) Studies reported with more than fifty patients with bacteremia caused by KP; iii) Eligible studies were included with no language restrictions. Exclusion criteria were as follows: i) *In vitro* studies, case reports or case series (n < 50), reviews, conference abstracts, study protocols, trial registrations, and duplicate publications; ii) Studies that included KPB patients as a sub-group or included only a sub-population of KPB patients based on infectious foci or patient characteristics, for the reason that these studies in most cases cannot derive the number of patients who died from KPB or provide sufficient information to evaluate the risk of bias.

The definitions were based on articles included in the study. KPB was defined as the isolation of KP in a blood culture specimen. CRKP was defined as resistant to at least one carbapenem or produced a carbapenemase. Hospital-acquired (HA) bacteremia was defined as a positive blood culture from a patient 48 hours after admission and no signs of infection had been noted at admission. Appropriate antibiotic therapy was defined as treatment regimen included at least one antimicrobial agent active *in vitro* against KP.

### Data extraction

Two authors independently extracted and then cross-checked the studies. The following data of the studies was extracted: the first author’s name, published year, study year(s), location, study design, patient characteristics (i.e., age, sex, and number of patients), and outcomes (7-day,14-day, 28- or 30-day, 90-day, and in hospital mortality rates if reported).

### Risk of bias assessment

The Risk of Bias in Nonrandomized Studies of Interventions (ROBINS-I) tool (Sterne JAC and Elbers, 2016) was used to evaluate the risk of bias of the included studies. This tool covers seven domains and refer to a hypothetical randomized trial as a “target” randomized trial. The seven domains were as follows: bias due to confounding, bias in selection of participants, bias in classification of interventions, bias due to deviations from intended interventions, bias due to missing data, bias in measurement of outcomes, and bias in selection of the reported result. Two investigators independently answered the signaling questions to judge for each bias domain. The overall risk of bias is categorized as ‘Low’, ‘Moderate’, ‘Serious’ or ‘Critical’. Any disagreement was resolved by a third researcher.

### Statistical analysis

The primary outcome was the 30-day mortality rate of single proportion after diagnosis of KPB; besides, the 7-, 14-, 90-day, and in hospital mortality rates were also analyzed. If two studies have same data while the endpoints were different, both studies would be included. Statistical heterogeneity among studies was determined by I^2^ statistics (degree of heterogeneity) and the Cochran Q test (p<0.05 indicated significant heterogeneity among studies). A random-effects model was used for pooled analysis in case of significant heterogeneity (I^2^>50%). Otherwise, the fixed-effects model was performed. Sensitivity analyses was performed by excluding one study in each turn to evaluate the influence of the individual trial on the overall pooled effects. The publication bias was assessed by visual inspection of the funnel plot and Peter’s test (Bai et al., 2022). If publication bias exsists, trim and filled model will be used to adjust for the funnel plot asymmetry.

In order to explore possible sources of heterogeneity (single proportion of 30-day mortality), a meta-regression analyse was conducted using a mixed-effects model. The variables were as follows: proportion of male, study design (classified into prospective or retrospective, cohort or case control or cross sectional), country (Asia, Europe, North America), study period (2006–2010, 2011–2015, 2016–2020, 2021–2022), mean or median age, proportion of ICU patients, proportion of patients used appropriate empirical antibiotic therapy, proportion of patients with CRKP bacteremia, proportion of patients with ESBL-KP bacteremia, and proportion of patients with hospital-acquired (HA) KPB. Subgroup analyses would be prespecified if significant factors were found in the meraregression model. Besides, pooled odds ratios (ORs) and 95% CI were further performed to compare mortality rates in patients with ESBL-KP or CRKP with those in patients with non-ESBL-KP or non-CRKP bacteremia.

The meta-analysis was conducted by R (version 4.0.3) package meta (Schwarzer, 2022).

## Results

### Study characteristics

**Figure 1:**
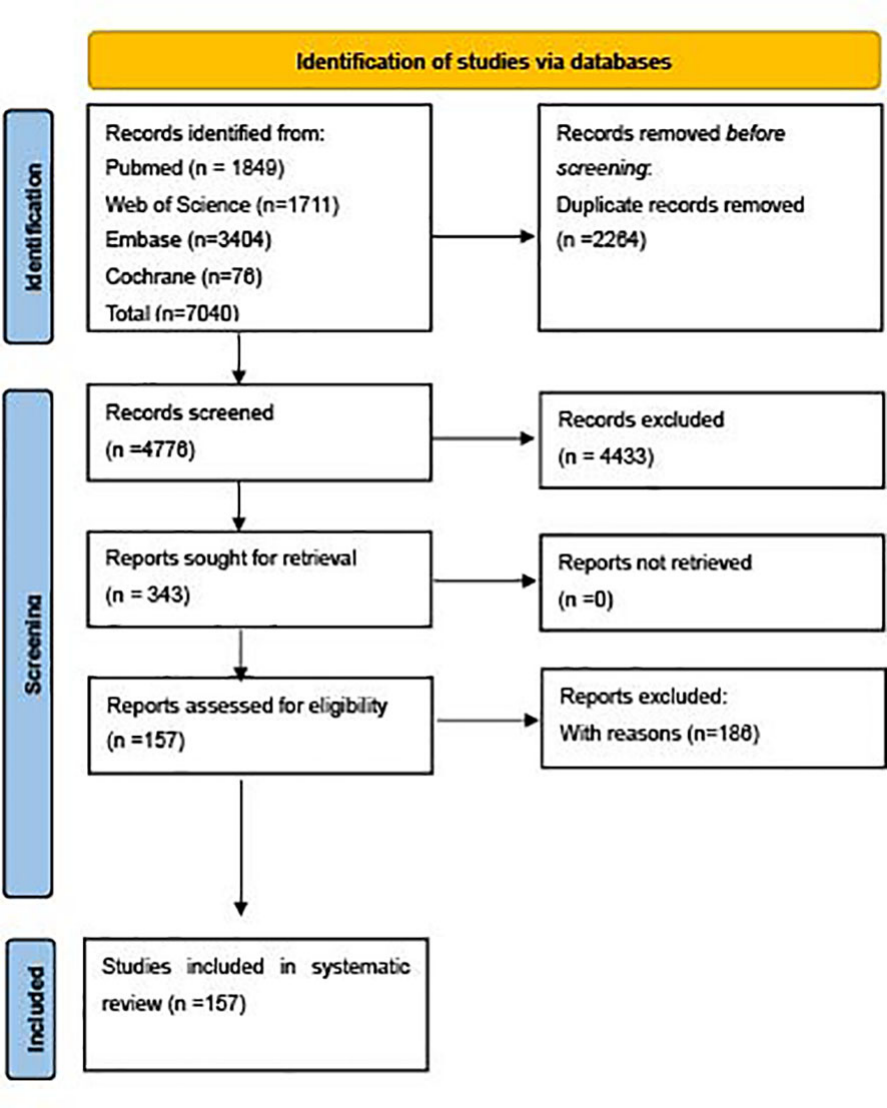
Flow of diagram of meta-analysis generated by PRISMA2020.

Of the 7,040 retrieved studies, 157 papers (37,915 enrolled patients) were included in the review (**Figure 1**). The references and studies with excluding reasons are listed in **Supplementary Materials 1 Text S2** and **Table S2**, respectively. The characteristics of the included 157 articles are described in **Supplementary Materials 1** **Table S3**, consisting of 18 prospective and 139 retrospective observational studies. Out of the 157 studies, 130 studies reported data from single centers and 27 studies reported data from multiple centers. These studies were published between 2001 and 2022, and the sample size varied from 50 to 5,712. The top five countries where studies were performed were China (65 studies), Italy (14 studies), South Korea (12 studies), Greece (10 studies), and Turkey (7 studies).

### Risk of bias assessment

The Robvis tool (McGuinness and Higgins, 2021) was used to assess publication quality by the risk of bias. The results for each study and each domain are presented in **Supplementary Materials 2** **Figure S1**. Since ROBINS-I tool emulates a hypothetical randomized trial to evaluate risk of bias in each domain, it is rare that an observational study would be judged as at low risk of bias in confounding domain. Therefore, most studies were judged as at least moderate overall risk of bias. In addition, for the reason that the primary study object was mortality rate, which was an objective measure, it was unlikely to be manipulated, almost all of studies included (except two studies) in the meta-analysis were judged as low risk of bias in measurement of outcomes domain. 149 (94.9%) studies were judged as serious or critical overall risk of bias, and the left 8 (5.1%) studies were at moderate overall risk of bias.

### Estimates of single proportion mortality rates in KPB

**Figure 2:**
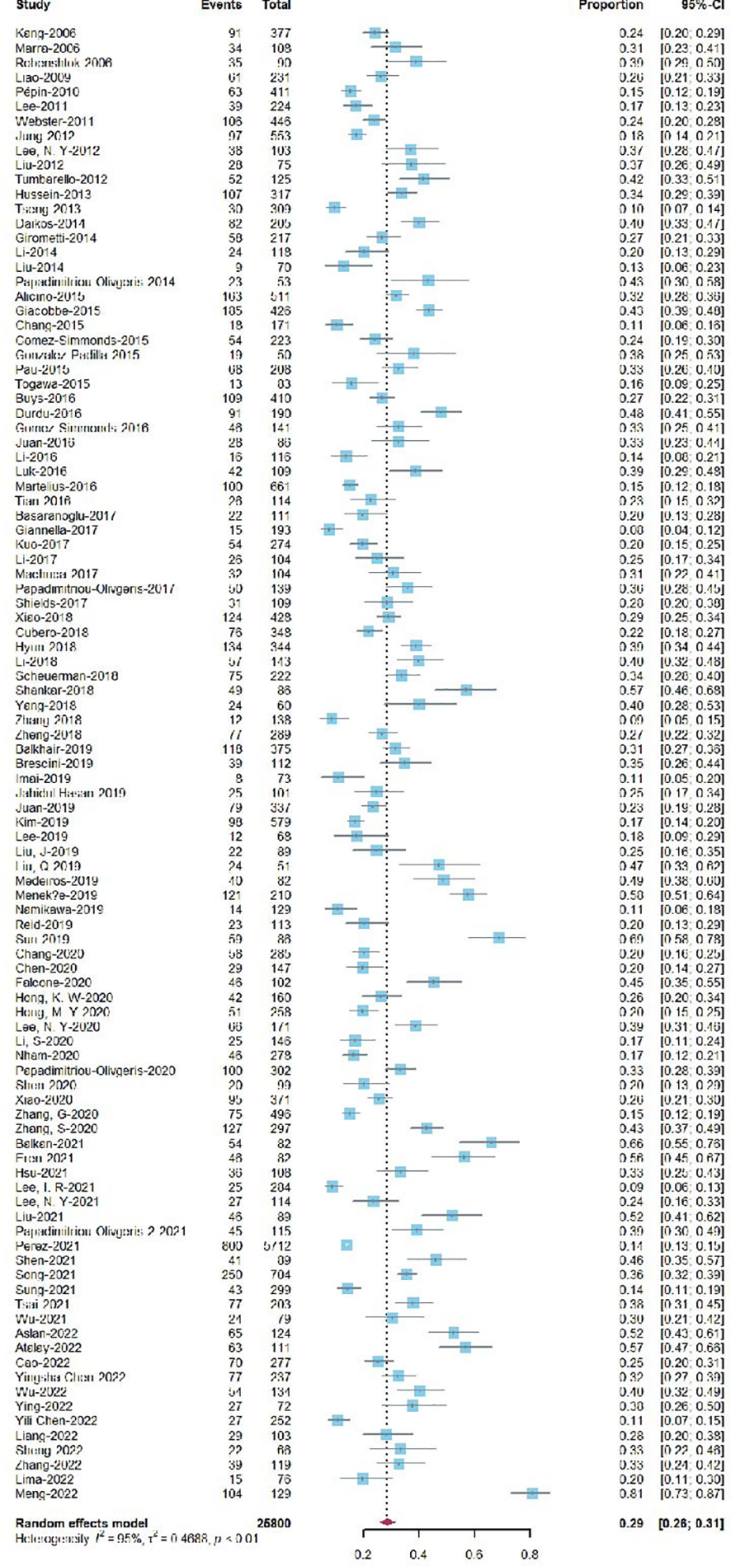
Forest plot of single proportion 30-day mortality for KPB.

A total of 101 studies reported 30-day mortality with a total of 25,800 patients, yielding a proportion of 29% (95% CI=0.26-0.31) (**Figure 2**). In addition, the pooled 7-, 14-, 90-day, and in hospital mortality rates for KPB are listed in **Supplementary Materials 2** **Figures S2-S5**, and the corresponding pooled death proportions were 17% (95% CI=0.14-0.20), 24% (95% CI=0.21-0.28), 34% (95% CI=0.26-0.42), and 29% (95% CI=0.26-0.33), respectively. Heterogeneities were observed high among all endpoints.

### Comparison of ESBL-KP and non-ESBL-KP bacteremia

The pooled mortality rates at different time points (14-, 28- or 30-day, and in hospital) with ESBL-KP *vs.* non-ESBL-KP are presented in **Supplementary Materials 2** **Figures S6-S8**. Overall, ESBL-KP bacteremia was associated with a higher mortality than non-ESBL-KP bacteremia. The pooled ORs at various time points (14-, 28- or 30-day, and in hospital) were 1.82 (95% CI 1.16-2.86), 1.57 (95% CI 1.07-2.32), and 1.57 (95% CI 1.23-2.02), respectively, with significant differences.

### Comparison of CRKP and non-CRKP bacteremia

**Figure 3:**
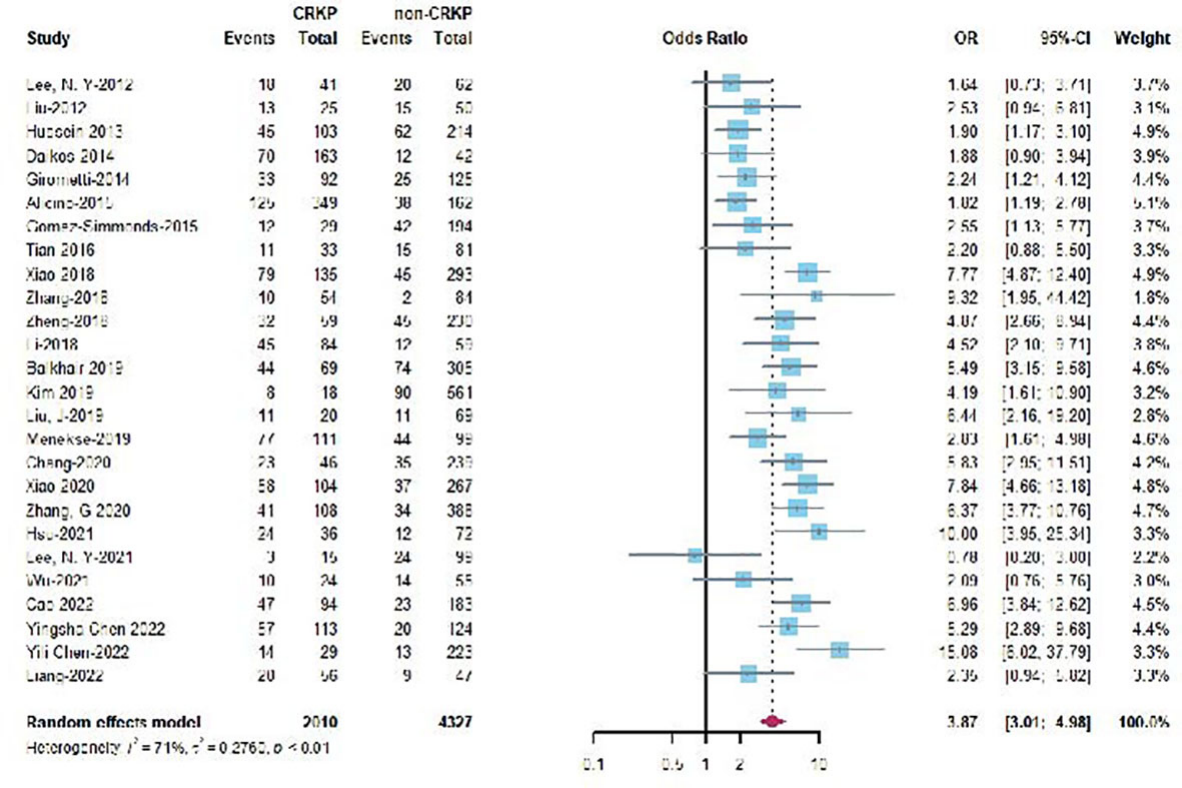
Forest plot of 30-day mortality for CRKP vs non-CRKP KPB.

A total of 26 studies assessed 30-day mortality for CRKP *vs*. non-CRKP bacteremia producing an OR of 3.87 (95% CI 3.01-3.49) (**Figure 3**). Overall, CRKP bacteremia was associated with a significant higher mortality than non-CRKP bacteremia. The forest plots of different endpoints (7-, 14-day, and in hospital) can be found in **Supplementary Materials 2** **Figures S9-S11**, and the corresponding pooled ORs at various endpoints (7-, 14-, in hospital) were 3.22 (95% CI 1.18-8.76), 5.66 (95% CI 4.31-7.42), and 4.05 (95% CI 3.38-4.85), respectively.

### Sensitivity analysis and publication bias

Sensitivity analysis was performed to evaluate the influence of an individual trial on the overall pooled effects. The Forest plots of sensitivity test for all KPB are presented in **Supplementary Materials 2** **Figures S12-S23**. In brief, almost all sensitivity analyses showed that exclusion of any individual study did not affect the overall pooled effects of mortality. In terms of publication bias, no significant asymmetry was observed in single proportion 7-day mortality, mortality (14-, 30-day and in-hospital) for ESBL *vs*. non-ESBL KPB, and mortality (7-, 14-, 30-day and in-hospital) for CRKP *vs*. non-CRKP KPB. A significant asymmetry was found in funnel plots for single proportion 14-, 30-day, and in-hospital mortality. The funnel plots and Peter’s tests are presented in **Supplementary Materials 2** **Figures S24-S35**. Trim and filled model was further used to adjust the funnel plot asymmetry in single proportion 14-, 30-day and in-hospital mortality. The adjusted funnel plots are presented in **Supplementary Materials 2** **Figures S36-S38**.

### Meta-regression and subgroup analysis of single proportion of 30-day mortality for KPB

Meta-regression showed that the potential sources of heterogeneity include the proportion of intensive care unit (ICU) patients, the proportion of HA patients, the proportion of patients with CRKP bacteremia, and the proportion of patients with ESBL-KP bacteremia (**Table 1**). Heterogeneity was not seen for factors including age, study period, study design, location, and the proportion of male, and appropriate empirical antibiotic therapy (**Table 1**). Subgroup analyses were further conducted to analyze the impact of the proportion of ICU, HA, CRKP, ESBL-KP on the 30-day mortality for KPB (**Table 2**). More than 50% of ICU, HA, CRKP, ESBL-KP were associated with a significantly higher 30-day mortality rates in patients with KPB. Partial heterogeneity was explained by these four factors

**Table 1:** Meta-regression results of single proportion of 30-day mortality for KPB.

Moderators		Number of studies	Proportion 95%-CI	Transformation	intrcpt	Test of Moderators P	heterogeneity		
							I^2 (%)^	tau^2^	p
Overall effect		101	0.28 (0.26; 0.31)	Logit	–	–	94.6	0.472	<0.001
Age (mean or median)	overall	92	0.30 (0.27; 0.33)	Logit	–	–	94.8	0.460	<0.001
	regression	–	–	–	-0.91	0.854	95.1	0.460	<0.001
Male (proportion)	overall	101	0.28 (0.26; 0.31)	Logit	–	–	94.6	0.472	<0.001
	regression	–	–	–	-1.96	0.111	95.1	0.459	<0.001
ICU (proportion)	overall	61	0.29 (0.26; 0.33)	Free-Tukey Double arcsine	–	–	94.6	0.023	<0.001
	regression	–	–		0.44	<0.001	93.2	0.016	<0.001
HA (proportion)	overall	48	0.27 (0.24; 0.30)	Logit	–	–	89.8	0.265	<0.001
	regression	–	–	–	-1.65	<0.001	88.4	0.201	<0.001
CRKP (proportion)	overall	69	0.32 (0.29; 0.36)	Logit	–	–	92.7	0.473	<0.001
	regression	–	–	–	-1.37	<0.001	91.1	0.289	<0.001
ESBL (proportion)	overall	24	0.21 (0.18; 0.25)	Logit	–	–	87.5	0.202	<0.001
	regression	–	–	–	-1.53	0.040	84.8	0.167	<0.001
Appropriate empirical antibiotic (proportion)	overall	52	0.31 (0.27; 0.35)	Free-Tukey Double arcsine	–	–	94.2	0.024	<0.001
	regression	–	–	–	0.66	0.245	94.5	0.024	<0.001
Location	overall	101	0.28 (0.26; 0.31)	Logit	–	–	94.6	0.472	<0.001
	regression	–	–	–	-0.94	0.405	94.5	0.457	<0.001
Design	overall	101	0.28 (0.26; 0.31)	Logit	–	–	94.6	0.472	<0.001
	regression	–	–	–	-1.53	0.585	94.8	0.440	<0.001
Period	overall	101	0.28 (0.26; 0.31)	Logit	–	–	94.6	0.472	<0.001
	regression	–	–	–	-1.04	0.265	94.8	0.453	<0.001

**Table 2:** Subgroup analyses on single proportion of 30-day mortality.

Moderators (proportion)	Number of studies	Proportion, 95%-CI	Transformation	P between Sub-groups	Heterogeneity		
					Tau^2^	P heterogeneity	I^2^ (%)
**ICU** (overall)	61	0.29 (0.26; 0.33)	Free-Tukey Double arcsine	< 0.001	0.023	< 0.001	94.6
<50	39	0.24 (0.21; 0.27)	–	–	0.014	< 0.001	92.7
>=50	22	0.37 (0.34; 0.46)	–	–	0.021	< 0.001	91.9
**HA** (overall)	48	0.27 (0.24; 0.30)	Logit	0.003	0.265	< 0.001	89.8
<50	13	0.21 (0.18; 0.25)	–	–	0.129	< 0.001	84.4
>=50	35	0.30 (0.26; 0.34)	–	–	0.261	< 0.001	90.1
**CRKP** (overall)	69	0.32 (0.29; 0.36)	Logit	< 0.001	0.473	< 0.001	92.7
<50	33	0.24 (0.21; 0.28)	–	–	0.243	< 0.001	89.6
>=50	36	0.41 (0.36; 0.46)	–	–	0.400	< 0.001	89.6
**ESBL** (overall)	24	0.21 (0.18; 0.25)	Logit	0.007	0.202	< 0.001	87.5
<50	18	0.19 (0.16; 0.23)	–	–	0.186	< 0.001	88.0
>=50	6	0.28 (0.23; 0.34)	–	–	0.076	< 0.001	71.0

## Discussion

In the systematic review of 157 studies on KPB, the pooled overall mortality rates were 17% at 7-day, 24% at 14-day, 29% at 30-day, 34% at 90-day, and 29% in hospital. It showed that more than 50% of ICU, HA, CRKP, ESBL-KP increased the 30-day mortality rates in patients with KPB.

KP belongs to *Enterobacteriaceae*, is one of the most life-threatening pathogens which can cause invasive infections (Zhang et al., 2022). Carbapenem-resistant Enterobacteriaceae (CRE) has been listed as “urgent threats” by the Centers for Disease Control and Prevention (CDC) (Chen et al., 2022). CRKP is one of the CRE species which the public most frequently encounter, accounting for 64-87.7% (Grundmann et al., 2017; Zhang et al., 2017; Li et al., 2021). However, with antimicrobial resistance increased annually causing a great health threat to the public, the accurate estimates of mortality rates for KPB have been insufficient. To our knowledge, this study provided the most up-to-date and comprehensive evidence of accurate estimation of KPB mortality rates. During the last decade, only one systematic review and meta-analysis (Kohler et al., 2017) evaluated the mortality rate of KPB including only 15 studies comparing CRKP *vs.* carbapenem-sensitive *Klebsiella pneumoniae* (CSKP). In the current study, the estimates of mortality rates for KPB were 17% at 7-day, 24% at 14-day, and 29% at 30-day, which were significantly higher than *E. coli* bacteremia (12.4%) reported by the systematic review (Bonten et al., 2021). The reasons for this scenario are not well-understood. However, there are several critical factors which might contribute to this disparity, for example patients with KPB are more likely to have a HA bacteremia, cardiovascular, or renal disease, and more likely to be in the ICU at the time of bacteremia diagnosis (Scheuerman et al., 2018). Additionally, to explore potential sources of heterogeneity, a meta-regression analyse was performed. Heterogeneity factors were found in the proportions of ICU patients, HA patients, and patients with CRKP or ESBL-KP bacteremia. However, when subgroup analyses were performed, few heterogeneities would be explained. It suggests that some other factors exist among these studies, such as the severity of illness, or the source of bacteremia. Due to the lack of data and different reporting scheme across studies, our analysis did not include these modulators. In addition, the results found a significantly higher 30-day mortality in patients with HA bacteremia. The same scenario happened for *Staphylococcus aureus* bacteremia reported in a systematic review and meta-analysis conducted by Bai and his colleagles (Bai et al., 2022). Previous studies (Tsay et al., 2002; Kang et al., 2006; Juan et al., 2019) revealed that nosocomial KPB was usually associated with a significantly higher mortality rate than community- acquired KPB. The current study re-emphasized the severity of nosocomial KPB.

In the present study, a significantly higher mortality rate in patients with CRKP bacteremia compared to those with non-CRKP bacteremia was found (**Figure 3**). The OR in each endpoint calculated by this study was higher than the previous study published in 2017 (Kohler et al., 2017). Alarmingly, although countermeasures have been taken to suppress these pathogens, the mortality rate of CRKP bacteremia in patients was obviously raised with the increased of CRKP. Most recently, clinicians have faced an even greater challenge that CRKP coexists with high virulence. The scenario has led lethal outbreaks in several hospitals (Gu et al., 2018; Mohammad Ali Tabrizi et al., 2018; Zhao et al., 2019). It is concerning that these pathogens would spread rapidly if effective measurements were not applied.

The study has several limitations. First, all the included studies were observational studies, and most were retrospective. The confounding domains (age, male, severity of disease, healthcare use and so on) did not overcome or not fully overcome in most studies, therefore, most included studies were judged as serious or critical risk of bias in confounding domains producing serious or critical overall risk of bias. Second, the heterogeneities were high at most endpoints. Although a meta-regression model was used to analyze the potential sources of heterogeneity factors, due to the lack of data and different reporting scheme across studies, the study did not include some significant modulators. Third, the definitions of CRKP are somewhat variable and the microbiological breakpoints changed over time. Additionally, the definitions of HA and appropriate antibiotic therapy are somewhat different as well.

In summary, the study provides the most up-to-date and comprehensive evidence of accurate estimation if KPB mortality rate. In addition, the proportion of ICU, HA, CRKP, ESBL-KP more than 50% increased the 30-day mortality rates in patients with KPB. It is concerning that the mortality rates of patients with CRKP bacteremia may raise with the increase of CRKP. Effective countermeasures should be taken to curb this scenario.

## Author contributions

JZ designed and supervised the study. DL and XH wrote the manuscript and revised the project. YL and HY performed the literature search. HR and SL checked the data. All authors approved the manuscript.
