# Supplementary material for: Klebsiella pneumoniae bacteremia mortality: a systematic review and meta-analysis
Source: Front Cell Infect Microbiol. 2023 Apr 20;13:1157010. doi: 10.3389/fcimb.2023.1157010 (PMC10159367; doi:10.3389/fcimb.2023.1157010)
Supplement: Supplementary file 1 [file DataSheet_1.doc]

**Supplement Materies 2**

**Table of Contents**

| Figure S1 ROBINS-I tool for assessing the risk of bias in included studies | Page 2-10 |
| --- | --- |
| Figure S2 Forest plot of single proportion 7-day mortality for KPB | Page 11 |
| Figure S3 Forest plot of single proportion 14-day mortality for KPB | Page 12 |
| Figure S4 Forest plot of single proportion 90-day mortality for KPB | Page 13 |
| Figure S5 Forest plot of single proportion in-hospital mortality for KPB | Page 14 |
| Figure S6 Forest plot of 14-day mortality for ESBL vs non-ESBL KPB | Page 15 |
| Figure S7 Forest plot of 28- or 30-day mortality for ESBL vs non-ESBL KPB | Page 16 |
| Figure S8 Forest plot of in-hospital mortality for ESBL vs non-ESBL KPB | Page 17 |
| Figure S9 Forest plot of 7-day mortality for CRKP vs non-CRKP KPB | Page 18 |
| Figure S10 Forest plot of 14-day mortality for CRKP vs non-CRKP KPB | Page 19 |
| Figure S11 Forest plot of in-hospital mortality for CRKP vs non-CRKP KPB | Page 20 |
| Figure S12 Forest plot of single proportion 7-day mortality sensitivity test for KPB | Page 21 |
| Figure S13 Forest plot of single proportion 14-day mortality sensitivity test for KPB | Page 22 |
| Figure S14 Forest plot of single proportion 28- or 30-day mortality sensitivity test for KPB | Page 23-24 |
| Figure S15 Forest plot of single proportion 90-day mortality sensitivity test for KPB | Page 25 |
| Figure S16 Forest plot of single proportion in-hospital mortality sensitivity test for KPB | Page 26 |
| Figure S17 Forest plot of 14-day mortality sensitivity test for ESBL vs non-ESBL KPB | Page 27 |
| Figure S18 Forest plot of 28- or 30-day mortality sensitivity test for ESBL vs non-ESBL KPB | Page 28 |
| Figure S19 Forest plot of in-hospital mortality sensitivity test for ESBL vs non-ESBL KPB | Page 29 |
| Figure S20 Forest plot of 7-day mortality sensitivity test for CRKP vs non-CRKP KPB | Page 30 |
| Figure S21 Forest plot of 14-day mortality sensitivity test for CRKP vs non-CRKP KPB | Page 31 |
| Figure S22 Forest plot of 30-day mortality sensitivity test for CRKP vs non-CRKP KPB | Page 32 |
| Figure S23 Forest plot of in-hospital mortality sensitivity test for CRKP vs non-CRKP KPB | Page 33 |
| Figure S24 Funnel plot of single proportion 7-day mortality for KPB | Page 34 |
| Figure S25 Funnel plot of single proportion 14-day mortality for KPB | Page 35 |
| Figure S26 Funnel plot of single proportion 30-day mortality for KPB | Page 36 |
| Figure S27 Funnel plot of single proportion 90-day mortality for KPB | Page 37 |
| Figure S28 Funnel plot of single proportion in-hospital mortality for KPB | Page 38 |
| Figure S29 Funnel plot of 14-day mortality for ESBL vs non-ESBL KPB | Page 39 |
| Figure S30 Funnel plot of 28- or 30-day mortality for ESBL vs non-ESBL KPB | Page 40 |
| Figure S31 Funnel plot of in-hospital mortality for ESBL vs non-ESBL KPB | Page 41 |
| Figure S32 Funnel plot of 7-day mortality for CRKP vs non-CRKP KPB | Page 42 |
| Figure S33 Funnel plot of 14-day mortality for CRKP vs non-CRKP KPB | Page 43 |
| Figure S34 Funnel plot of 28- or 30-day mortality for CRKP vs non-CRKP KPB | Page 44 |
| Figure S35 Funnel plot of in-hospital mortality for CRKP vs non-CRKP KPB | Page 45 |
| Figure S36 Funnel plot of single proportion 14-day mortality for KPB in trim and filled model | Page 46 |
| Figure S37 Funnel plot of single proportion 28- or 30-day mortality for KPB in trim and filled model | Page 47 |
| Figure S38 Funnel plot of single proportion in-hospital mortality for KPB in trim and filled model | Page 48 |

**Figure S1 ROBINS-I tool for assessing risk of bias in included studies**

**
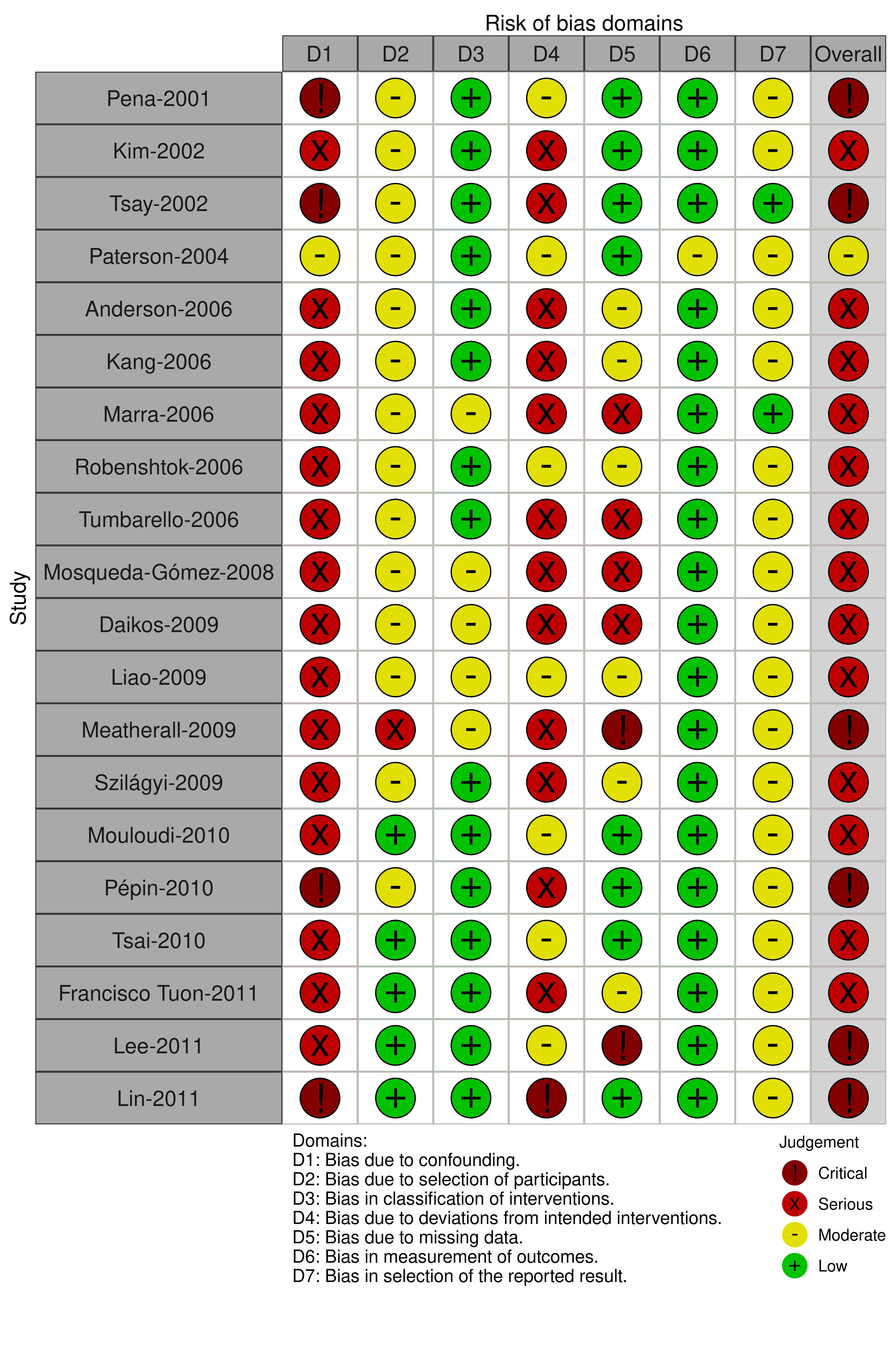
**

**Continued**

**
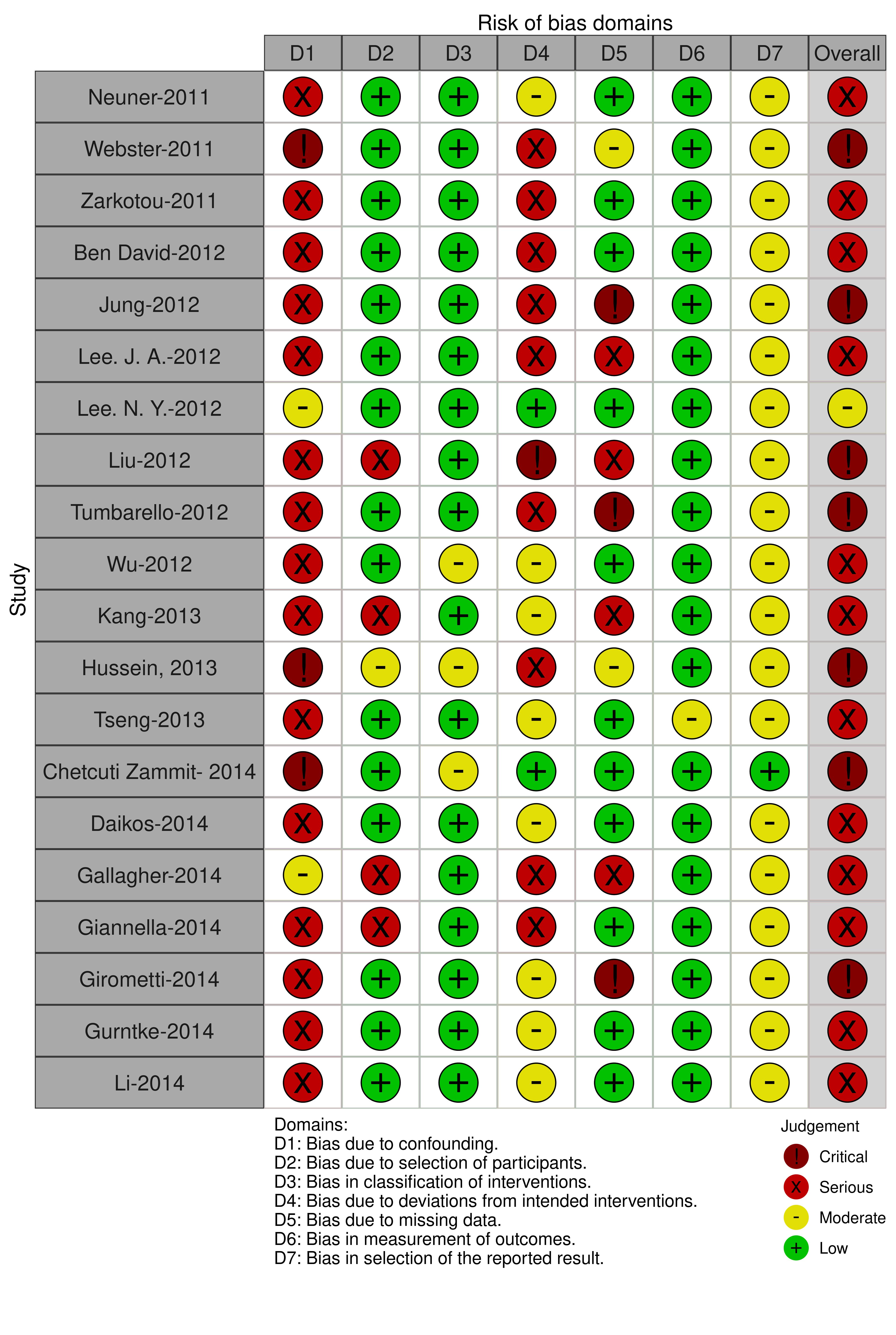
**

**Continued**

**
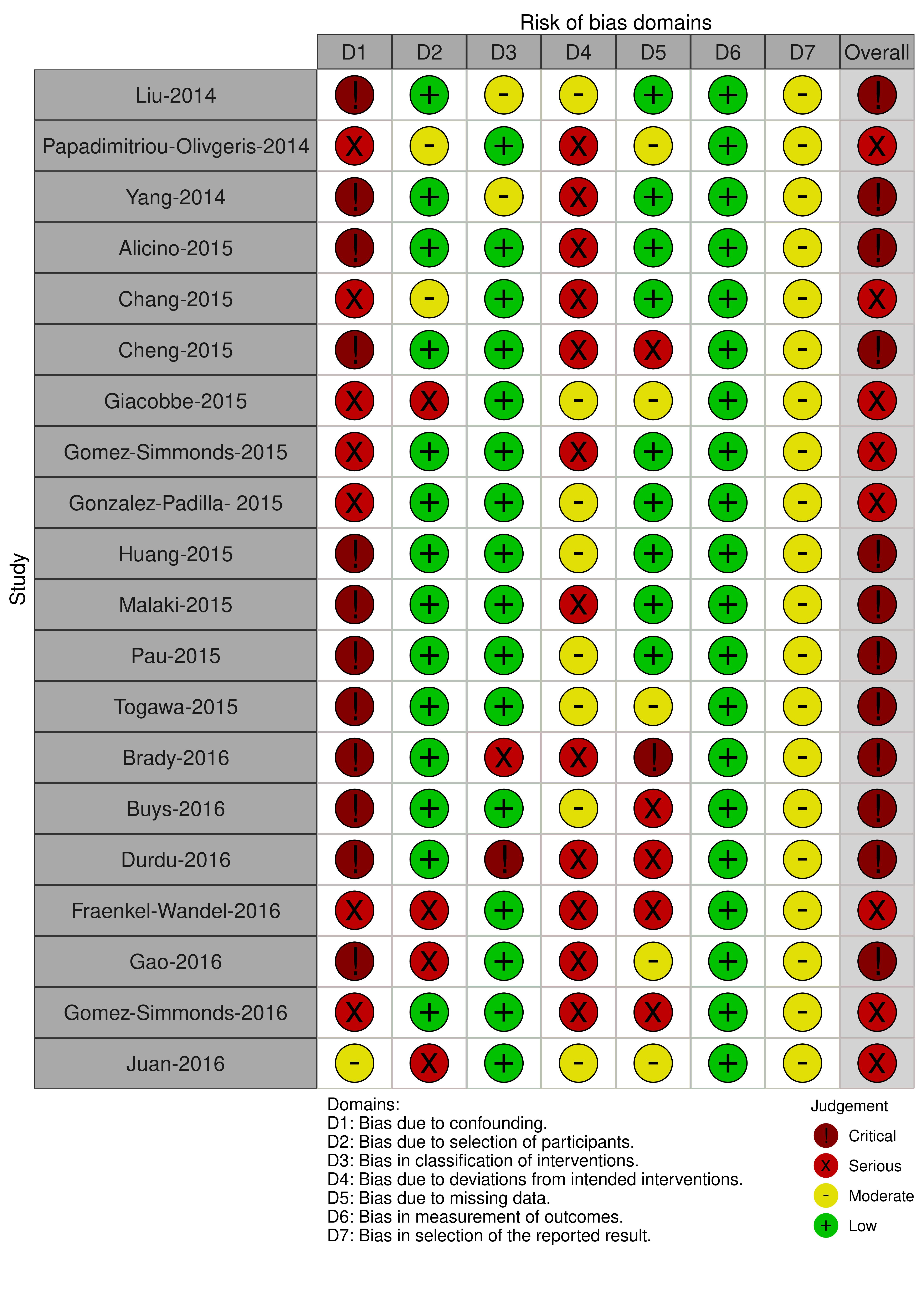
**

**Continued**


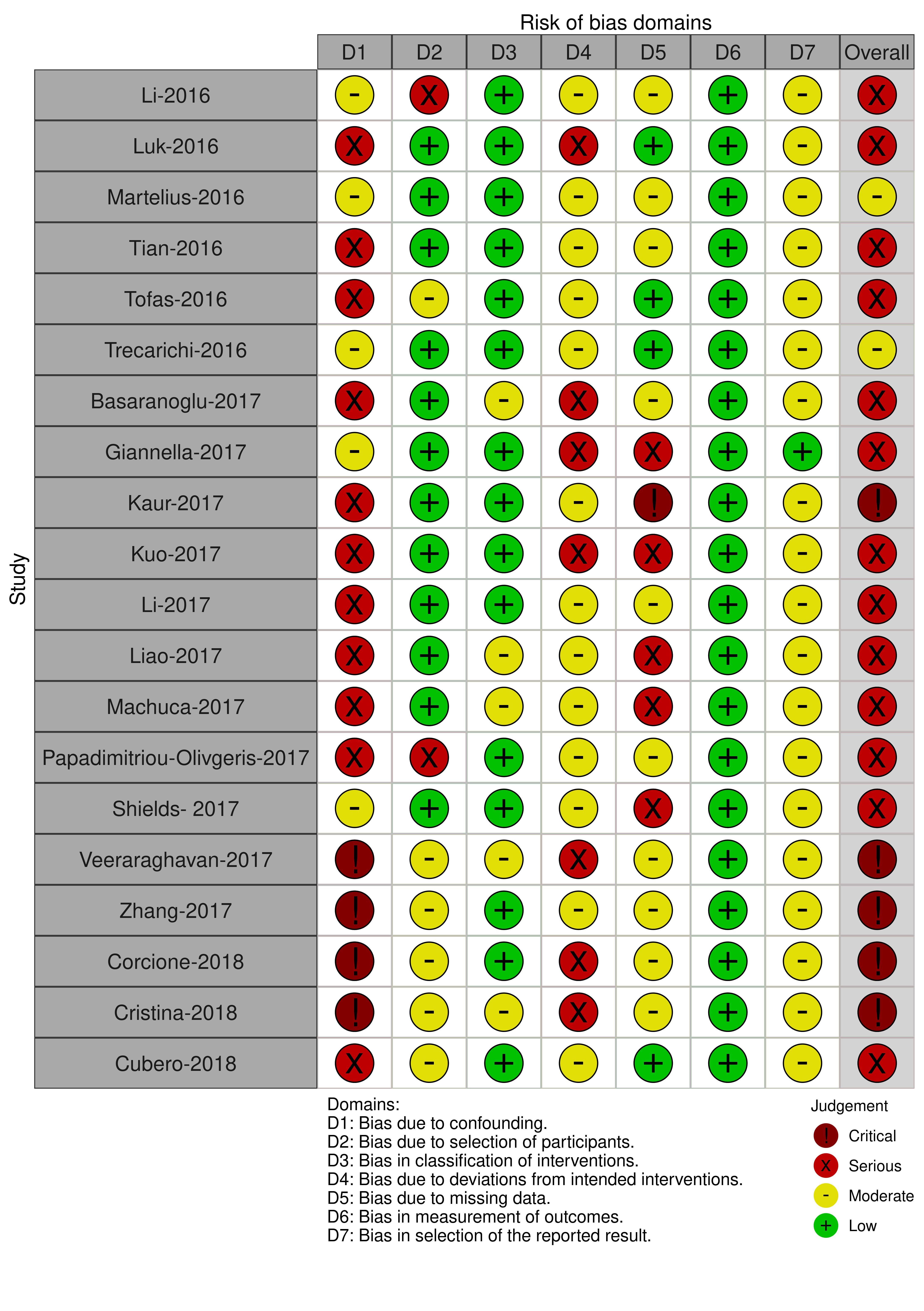


**Continued**


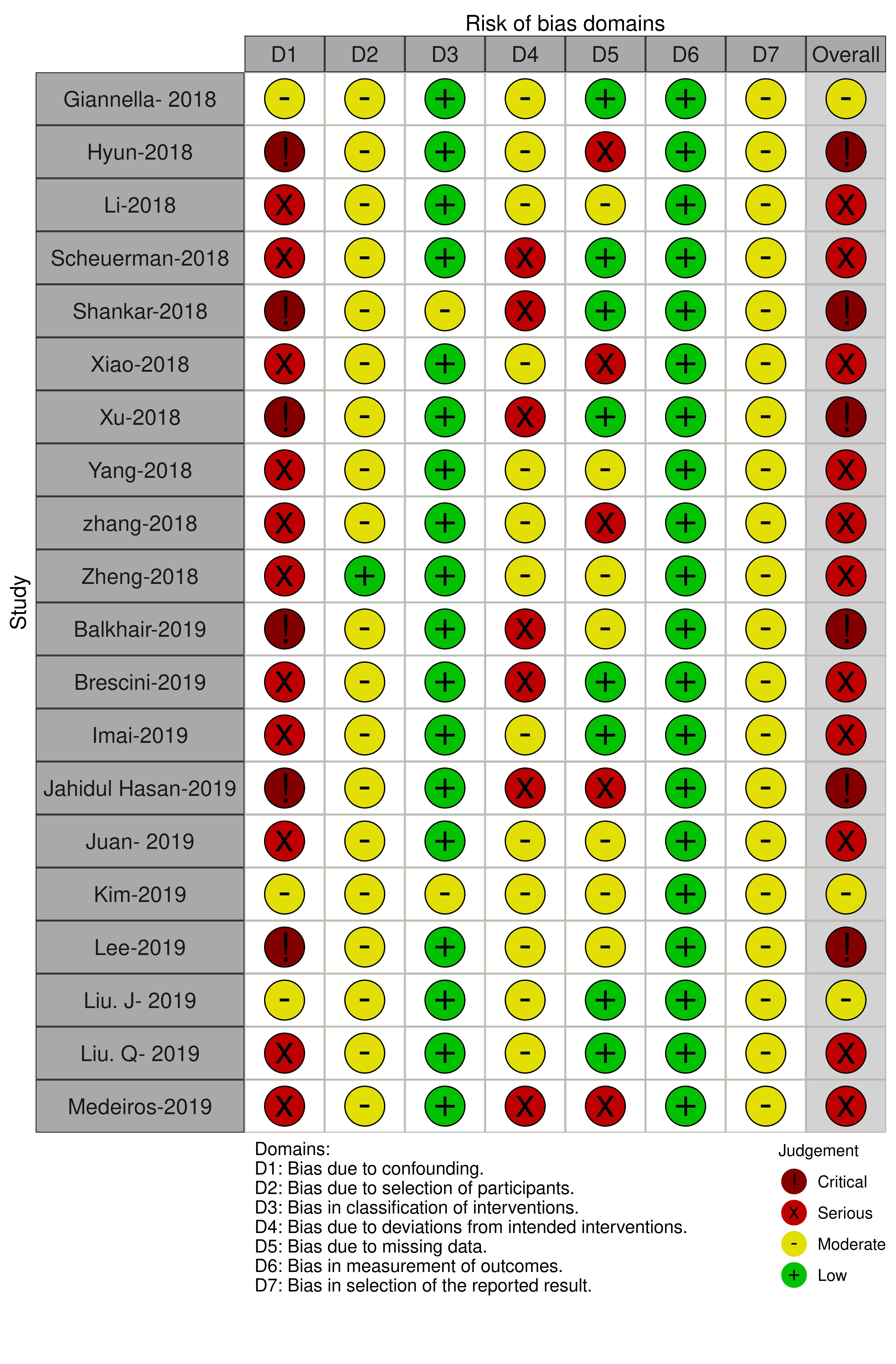


**Continued**


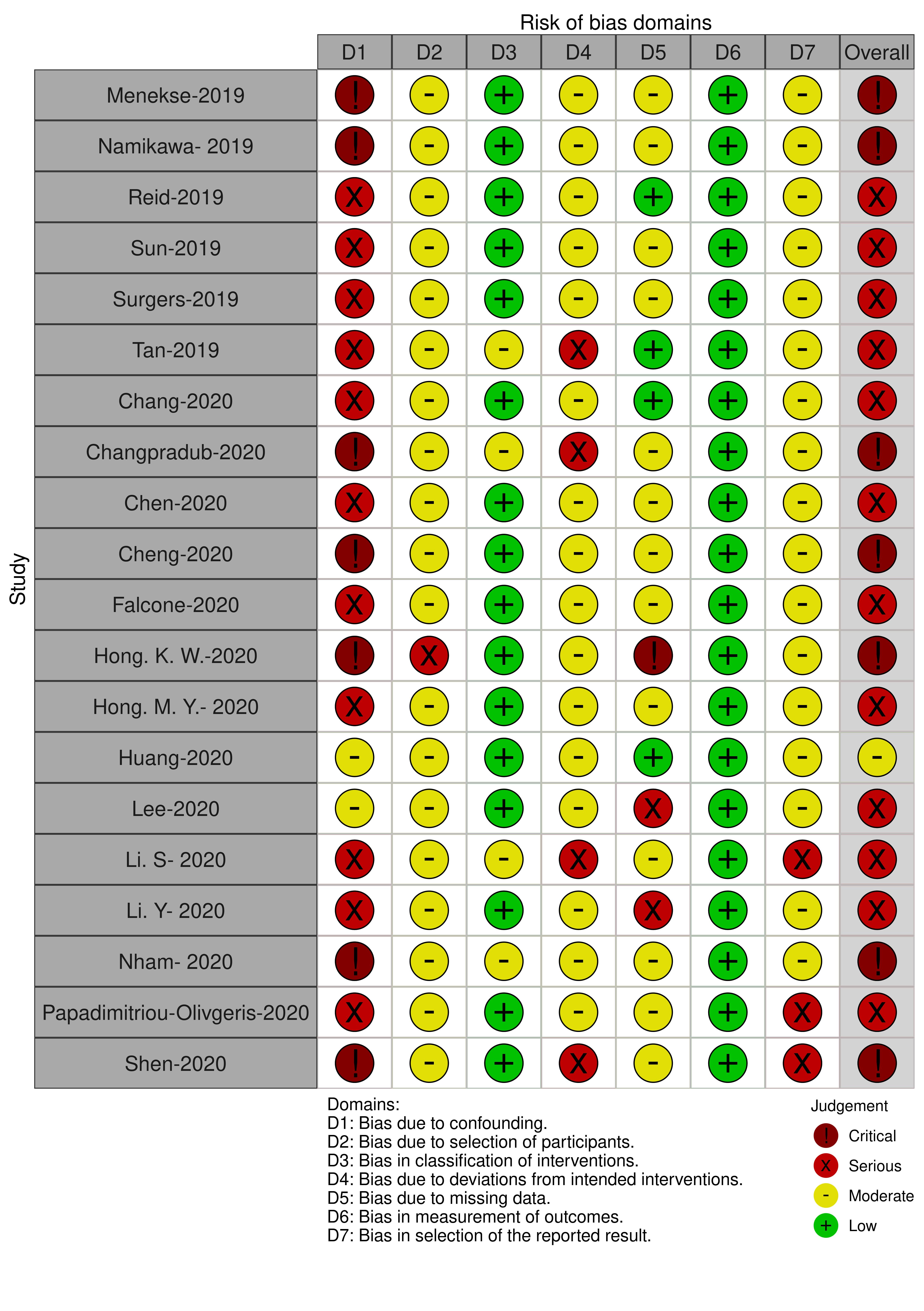


**Continued**


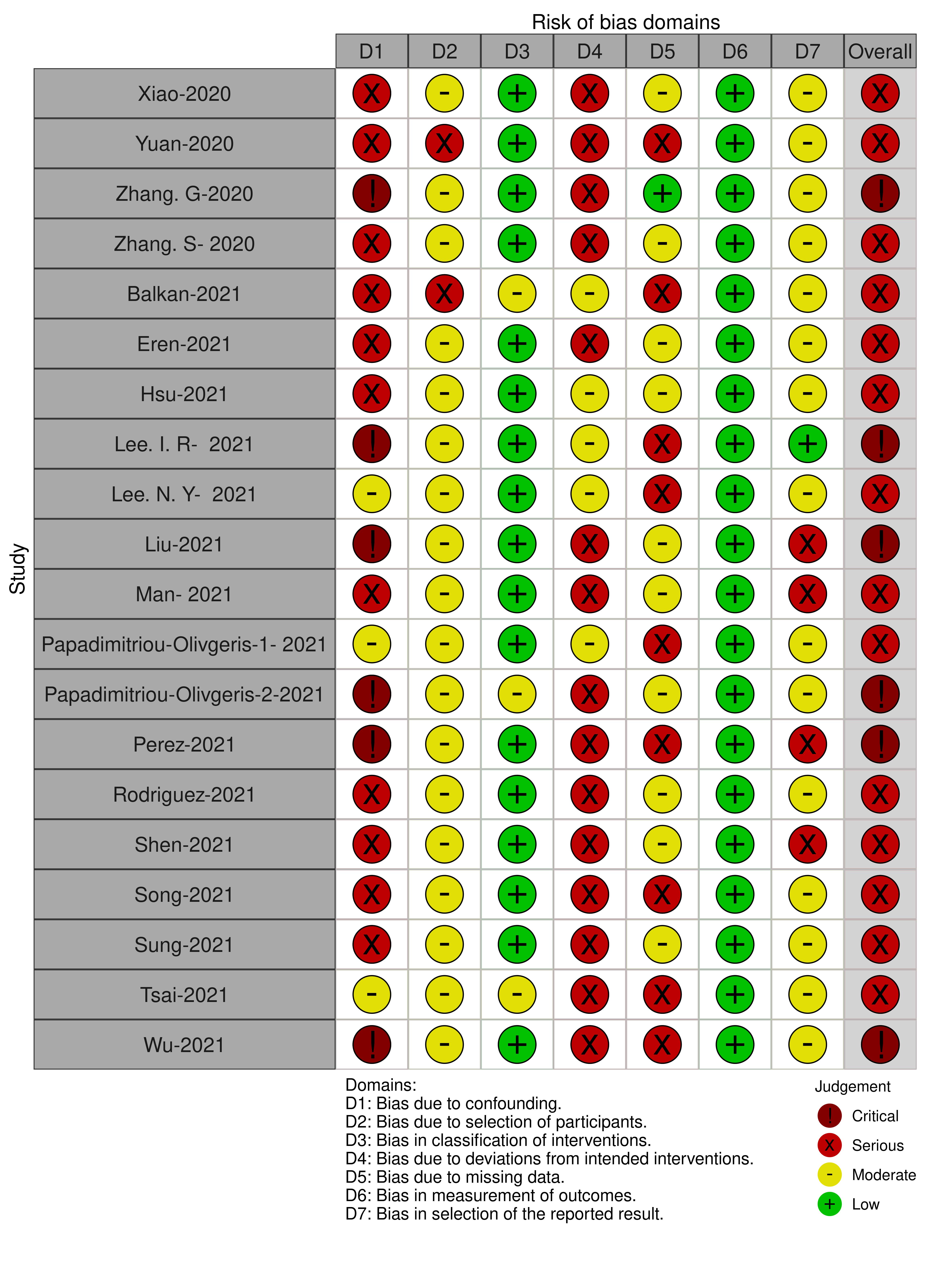


**Continued**


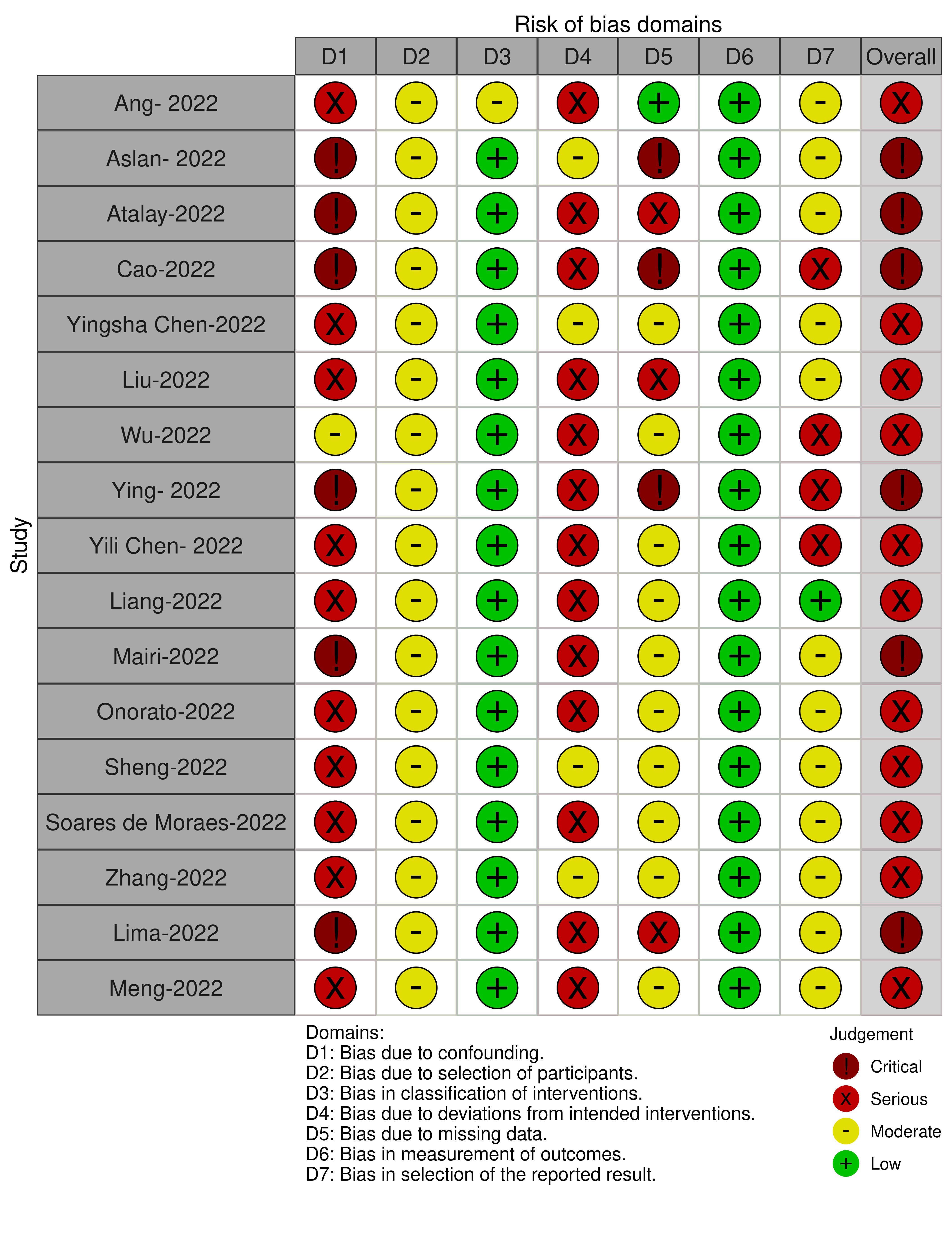


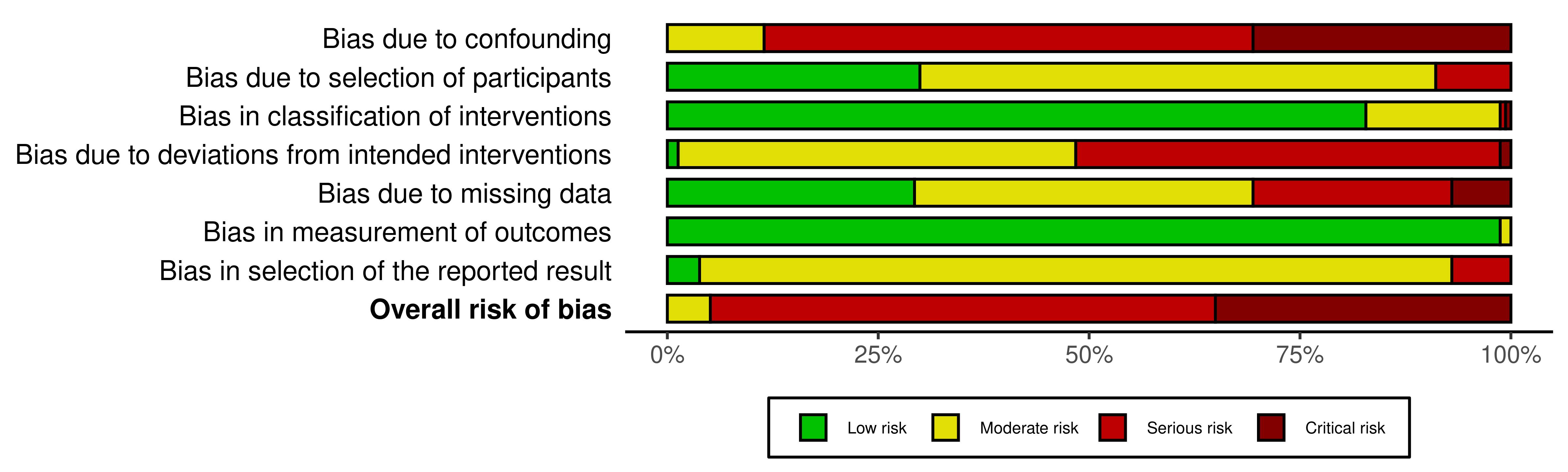


**Figure S2 Forest plot of single proportion 7-day mortality for KPB**


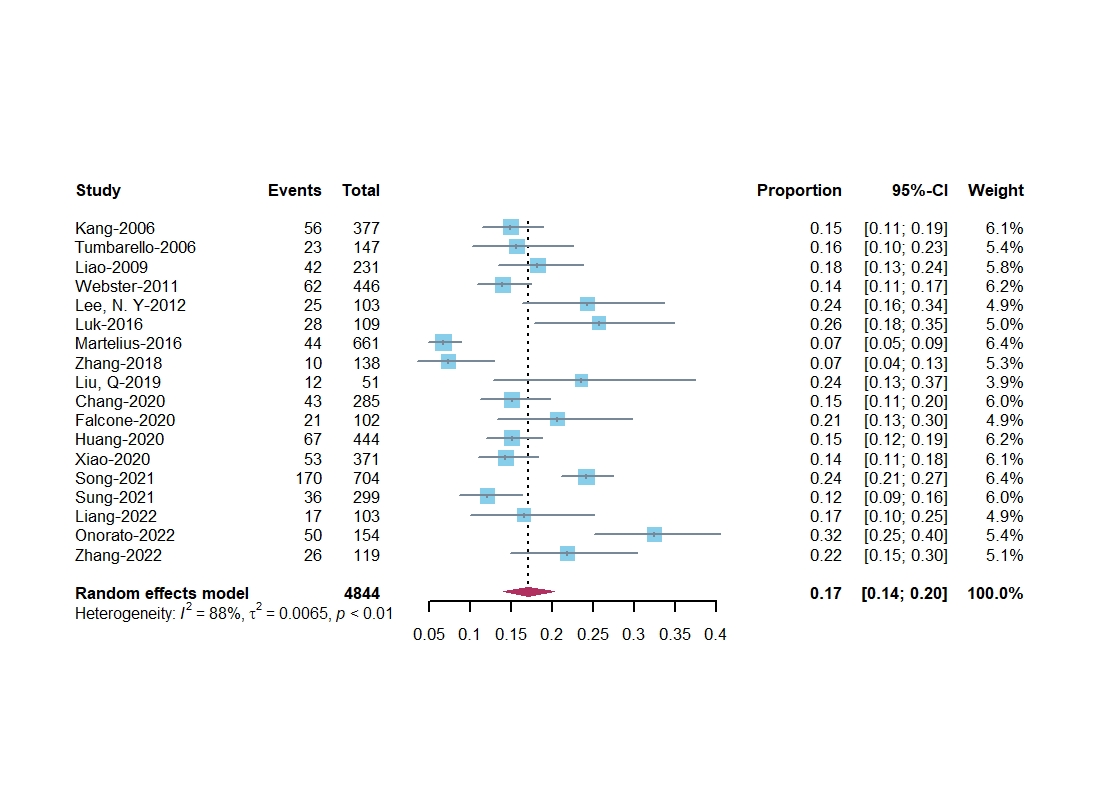


**Figure S3 Forest plot of single proportion 14-day mortality for KPB**


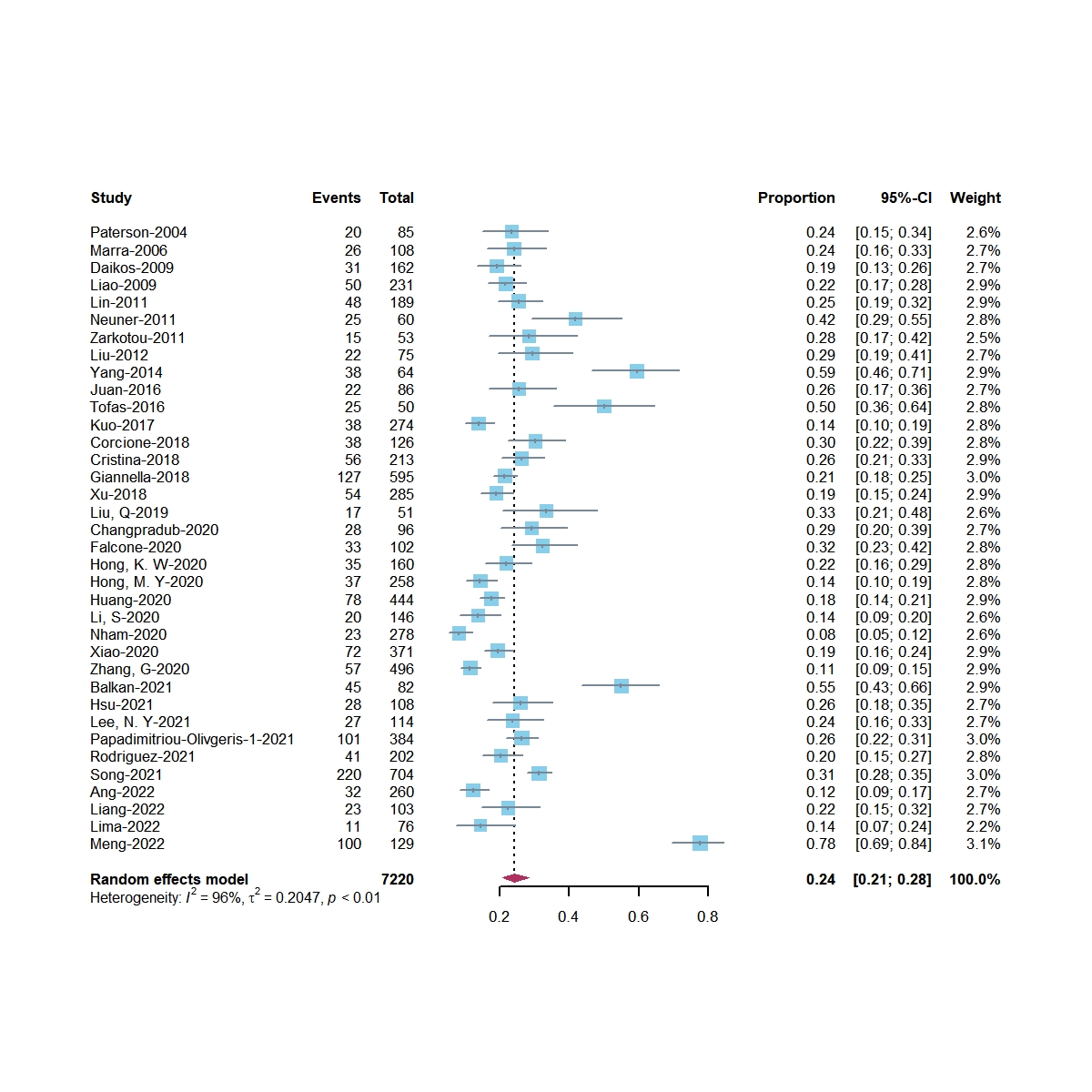


**Figure S4 Forest plot of single proportion 90-day mortality for KPB**


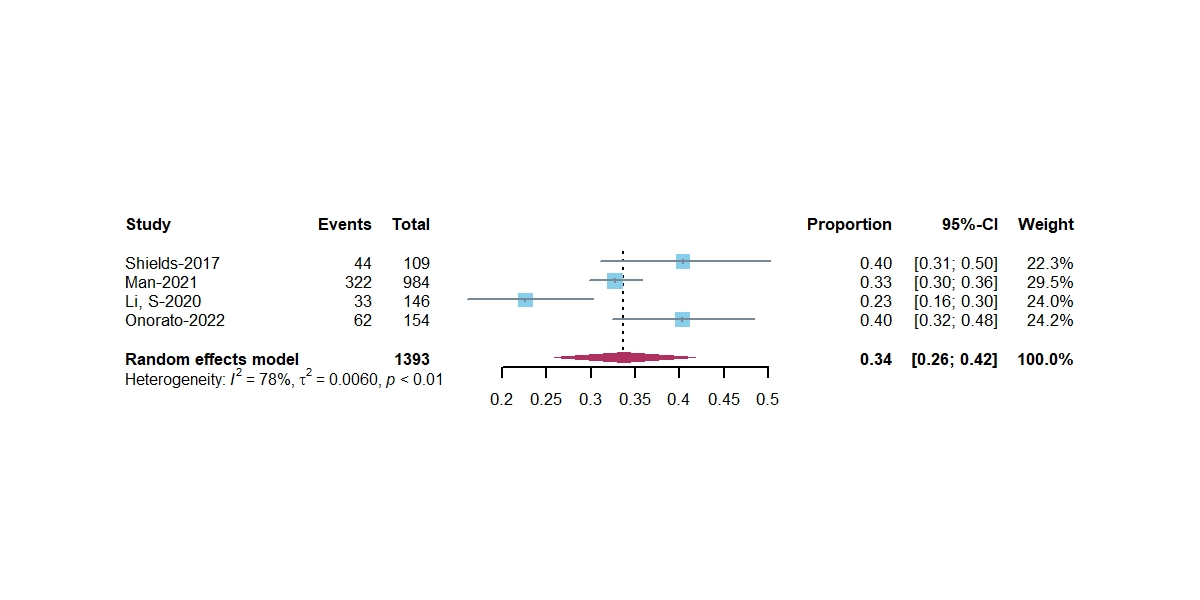


**Figure S5 Forest plot of single proportion in-hospital mortality for KPB**


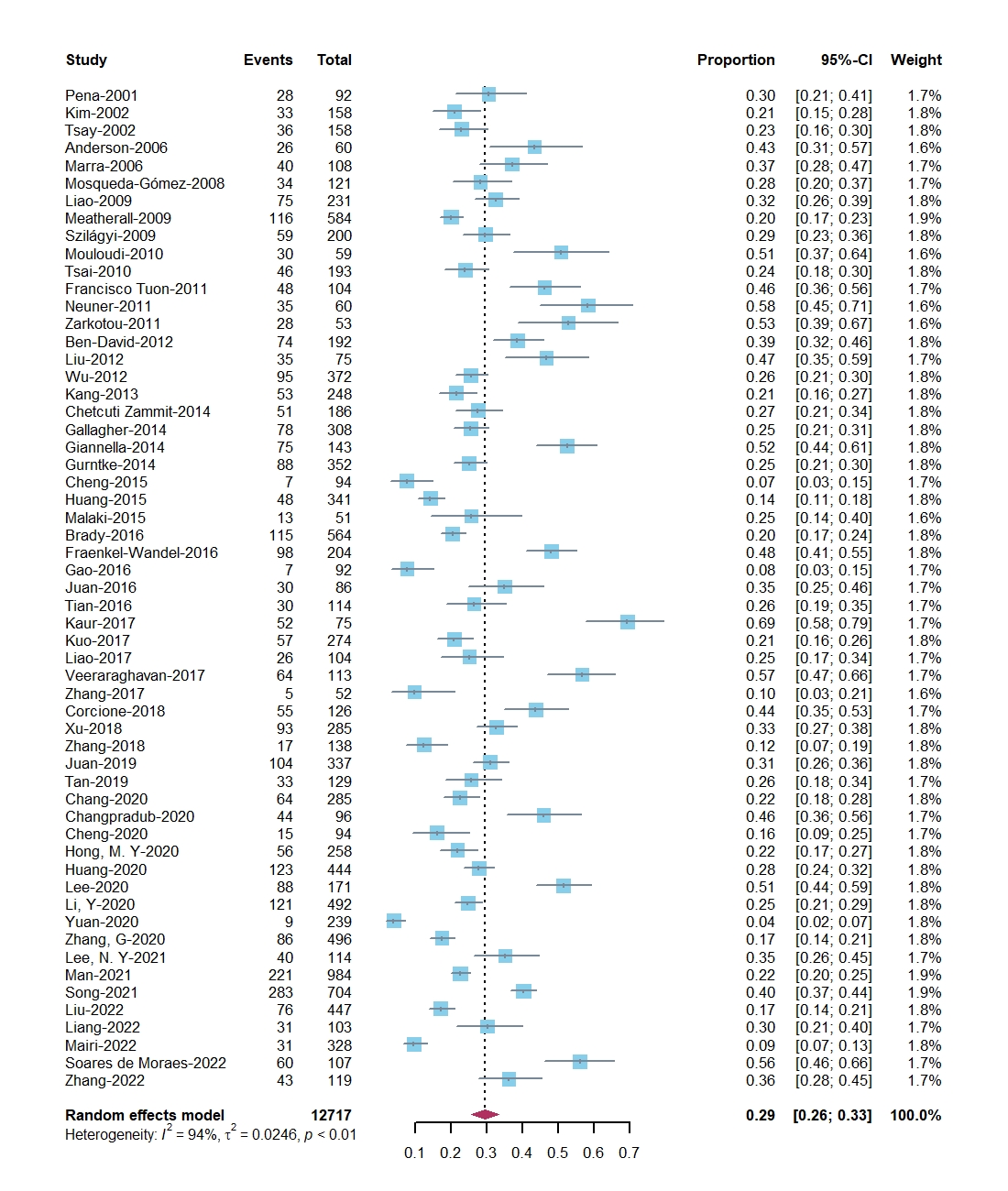


**Figure S6 Forest plot of 14-day mortality for ESBL vs non-ESBL KPB**


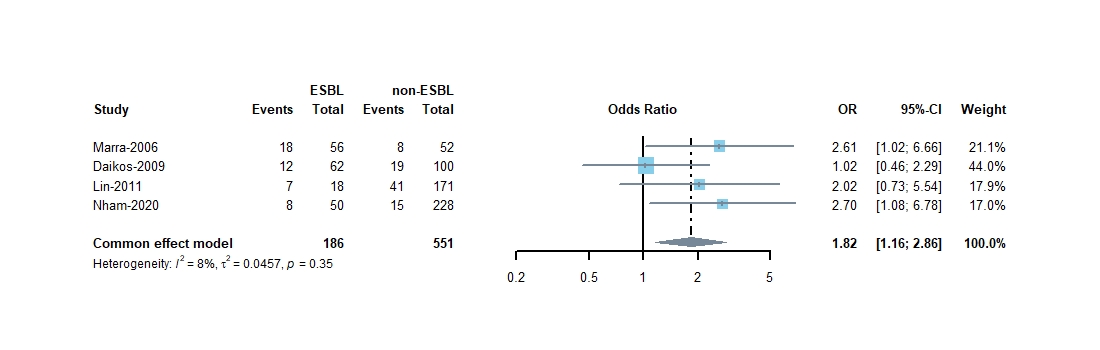


**Figure S7 Forest plot of 28- or 30-day mortality for ESBL vs non-ESBL KPB**


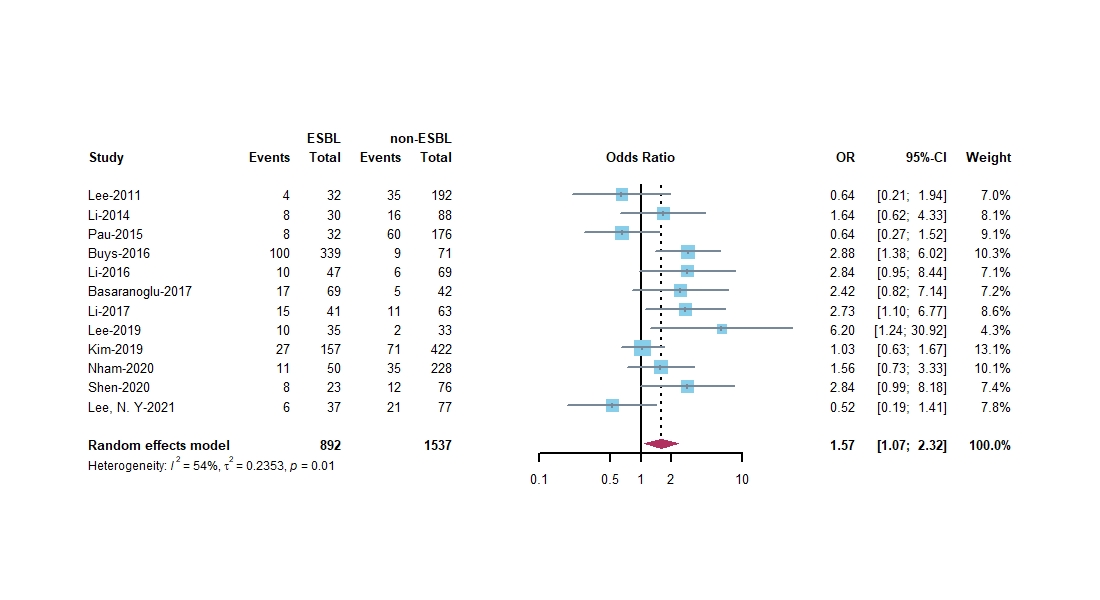


**Figure S8 Forest plot of in-hospital mortality for ESBL vs non-ESBL KPB**


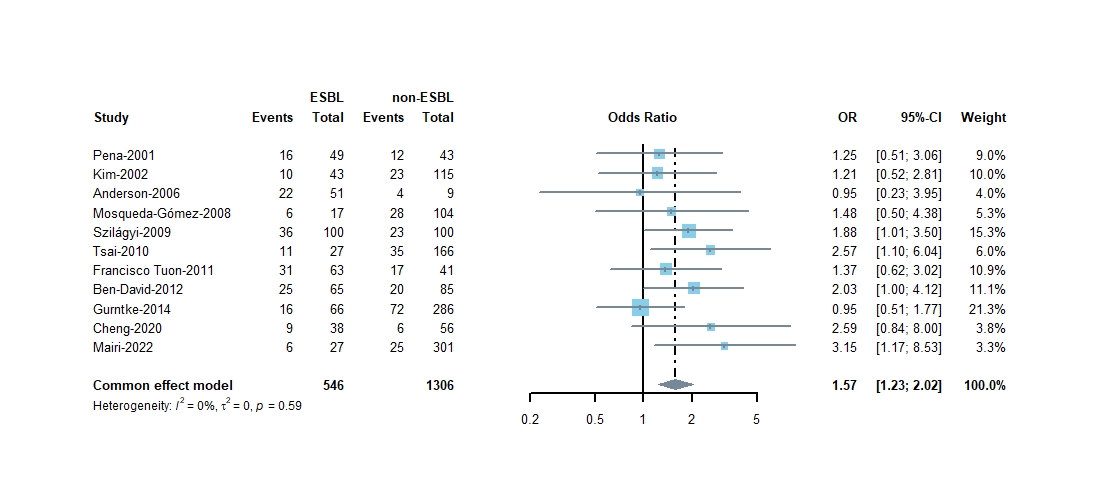


**Figure S9 Forest plot of 7-day mortality for CRKP vs non-CRKP KPB**


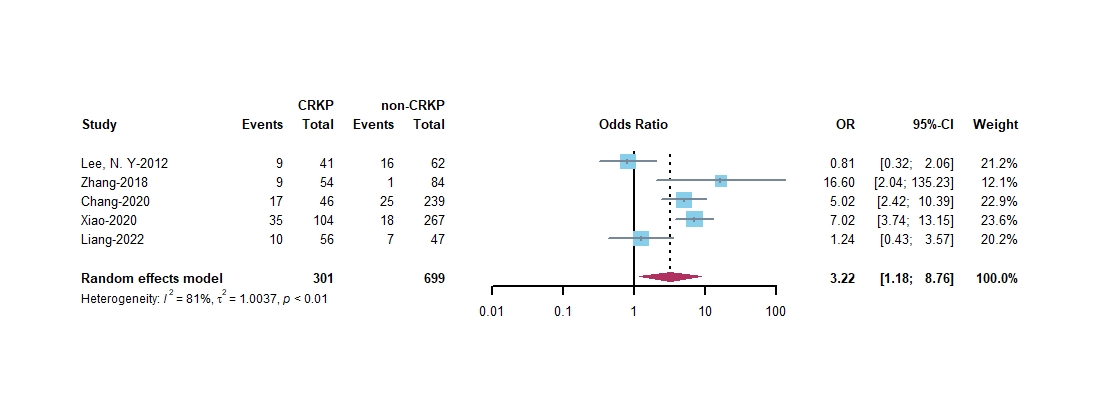


**Figure S10 Forest plot of 14-day mortality for CRKP vs non-CRKP KPB**


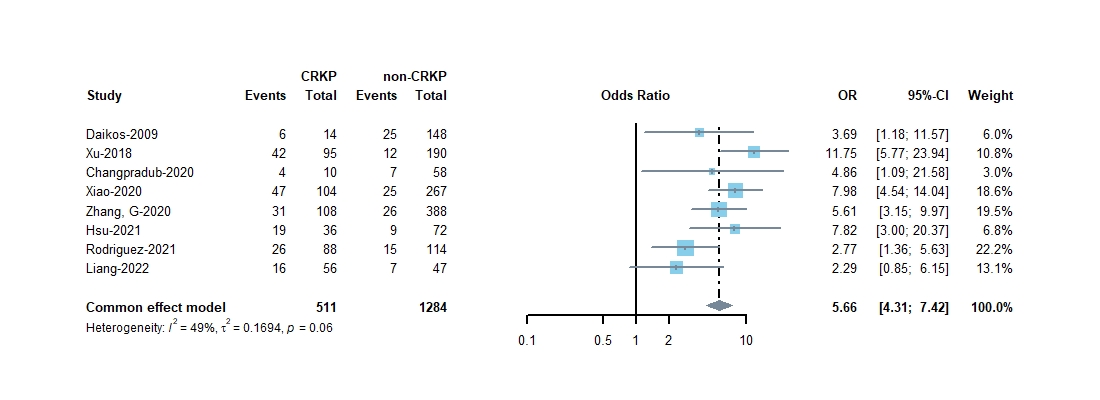


**Figure S11 Forest plot of in-hospital mortality for CRKP vs non-CRKP KPB**


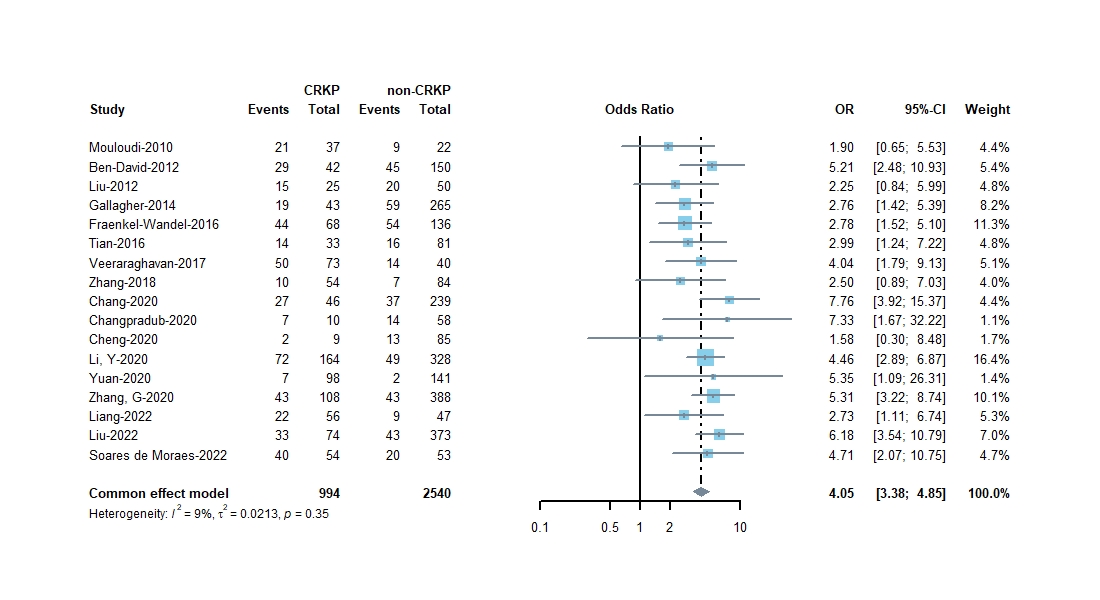


**Figure S12 Forest plot of single proportion 7-day mortality sensitivity test for KPB**

**
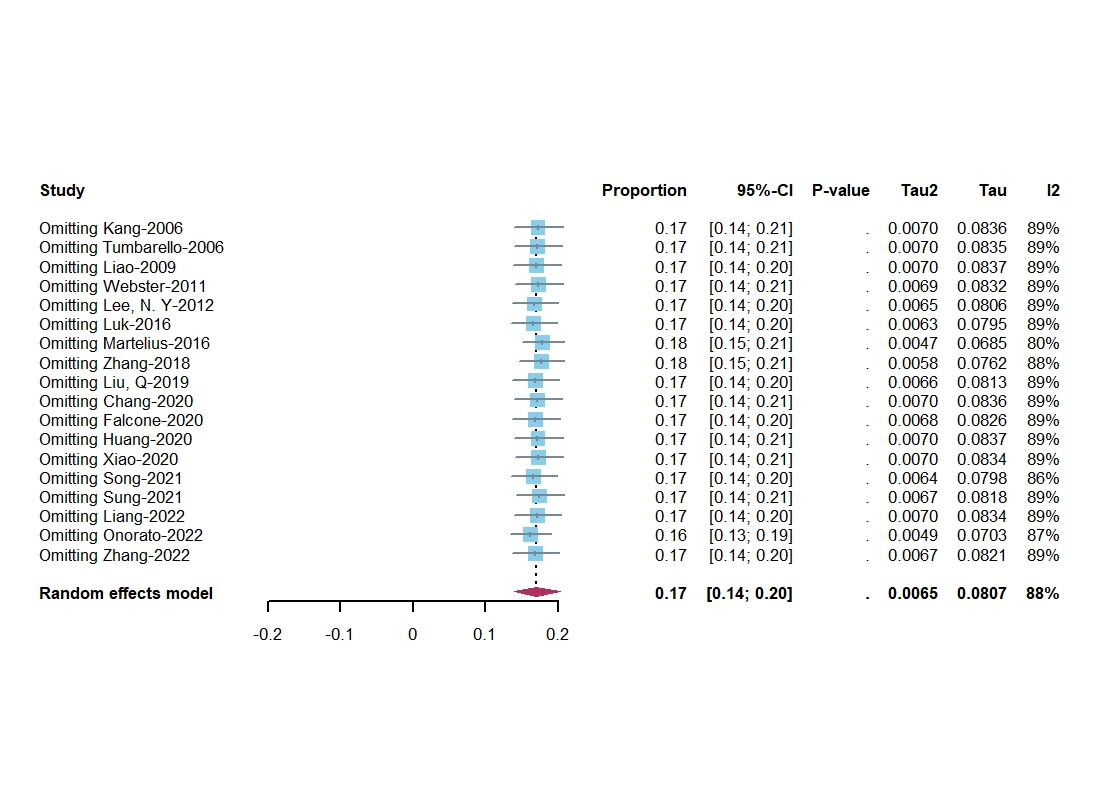
**

**Figure S13 Forest plot of single proportion 14-day mortality sensitivity test for KPB**


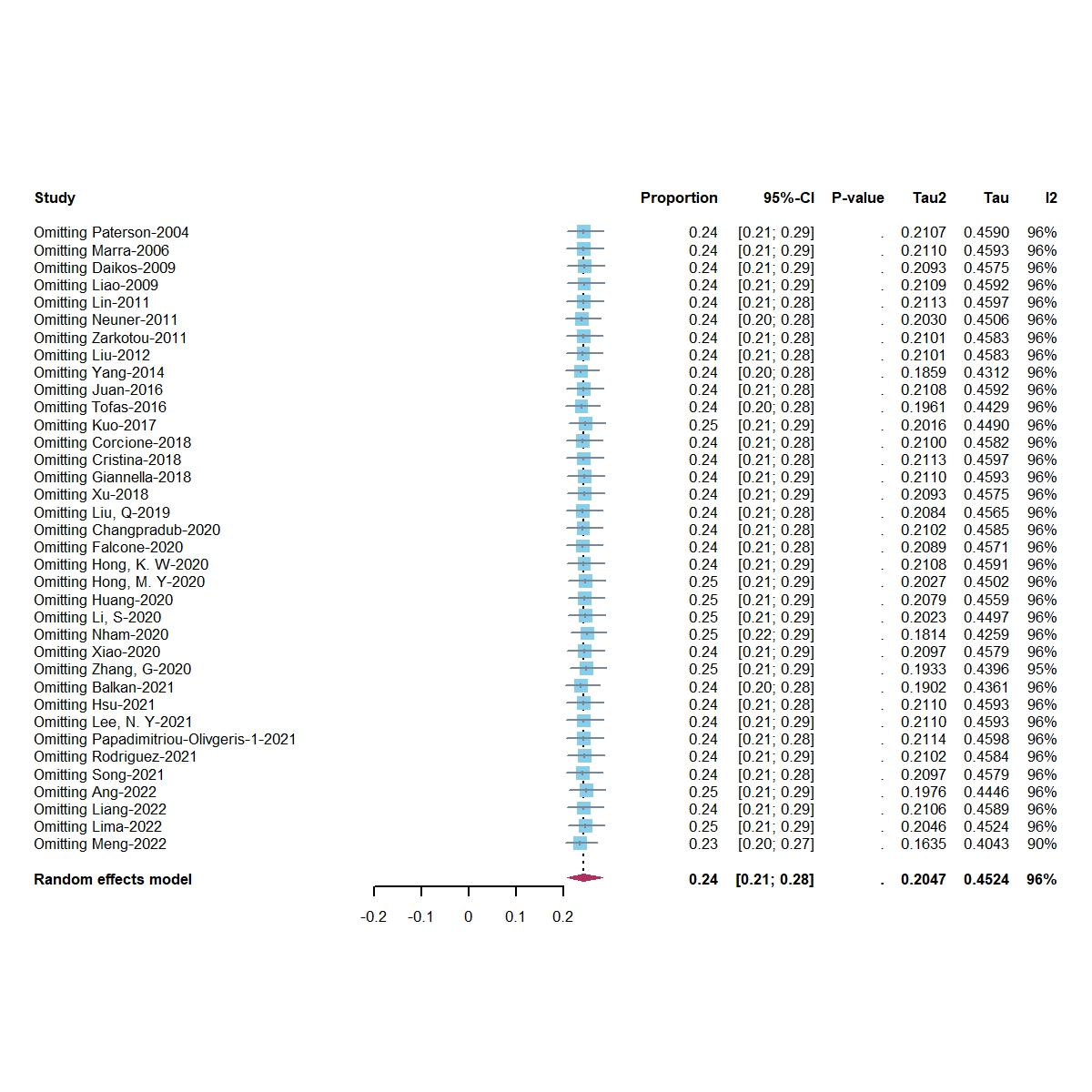


**Figure S14 Forest plot of single proportion 28- or 30-day mortality sensitivity test for KPB**


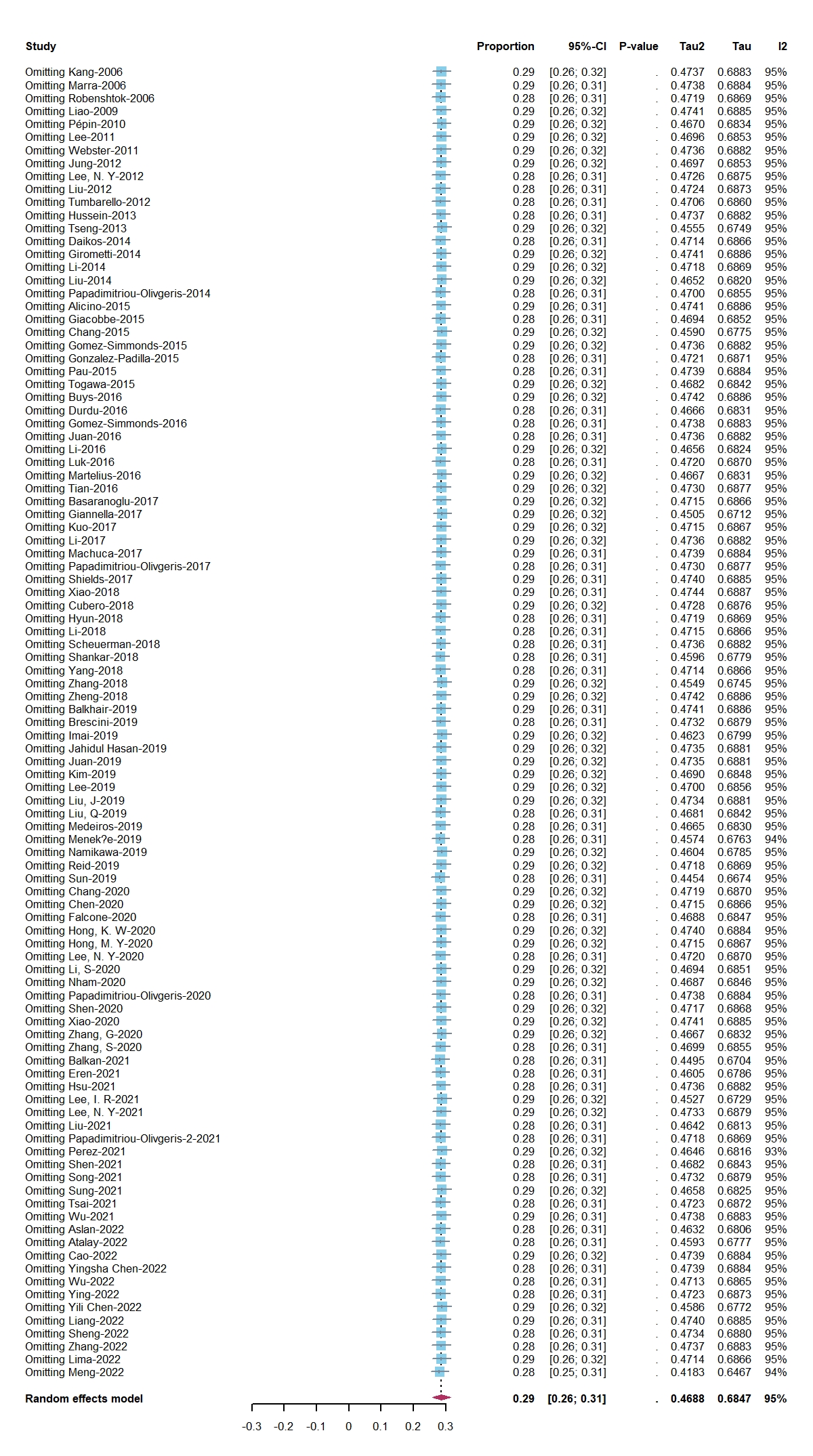


**Figure S15 Forest plot of single proportion 90-day mortality sensitivity test for KPB**


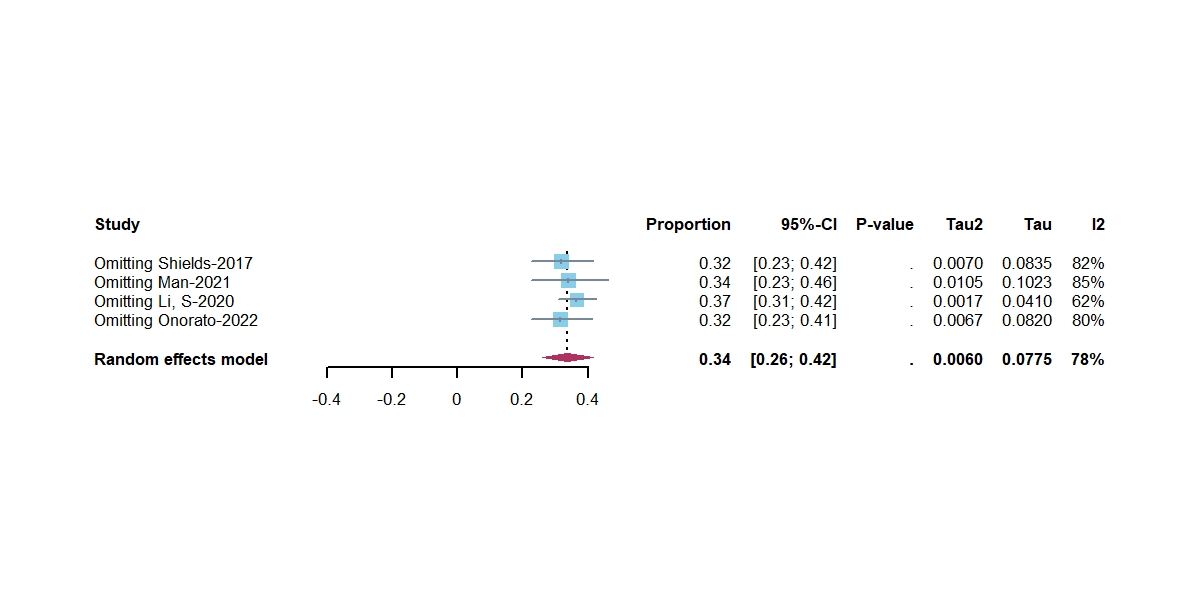


**Figure S16 Forest plot of single proportion in-hospital mortality sensitivity test for KPB**


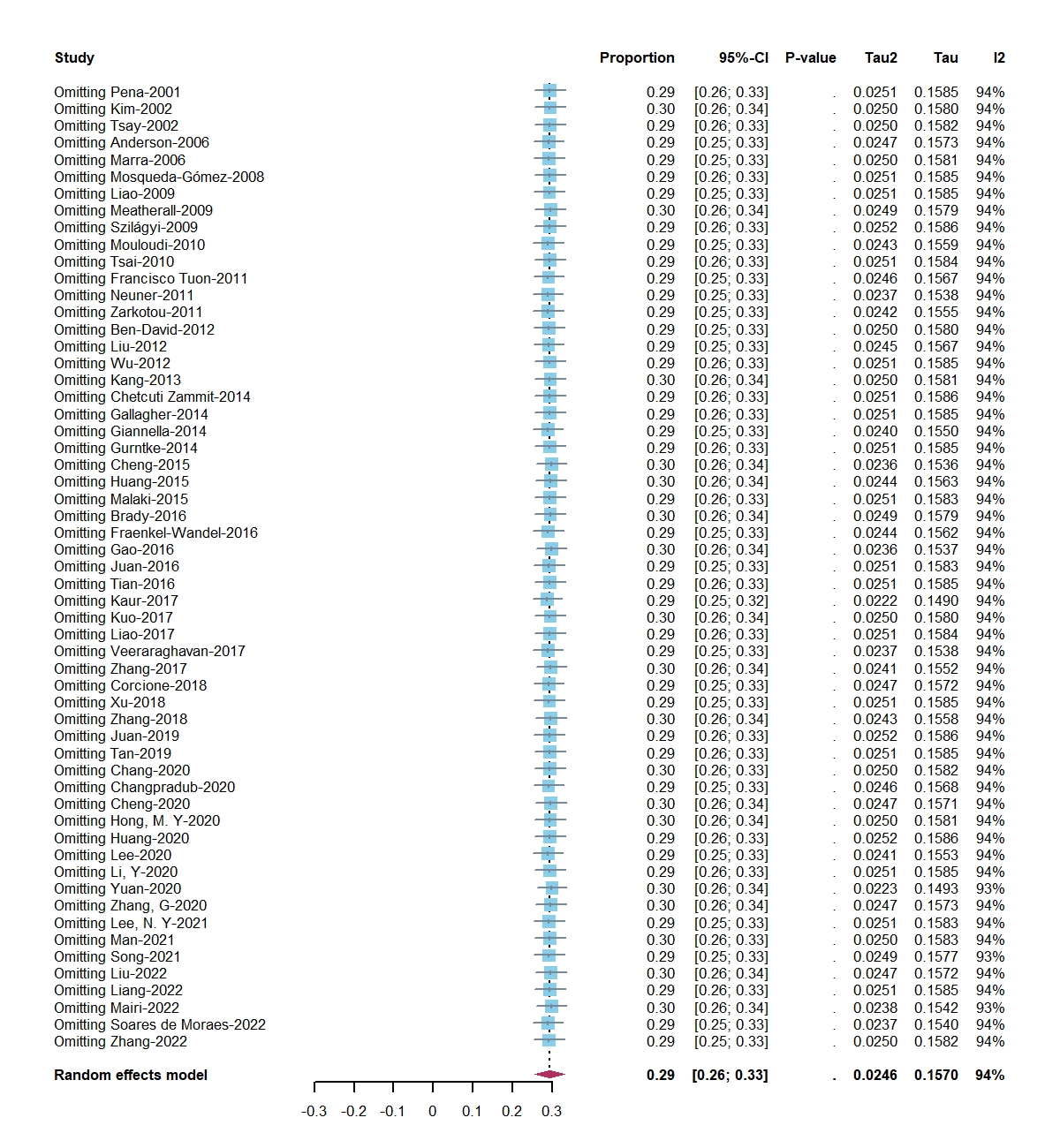


**Figure S17 Forest plot of 14-day mortality sensitivity test for ESBL vs non-ESBL KPB**


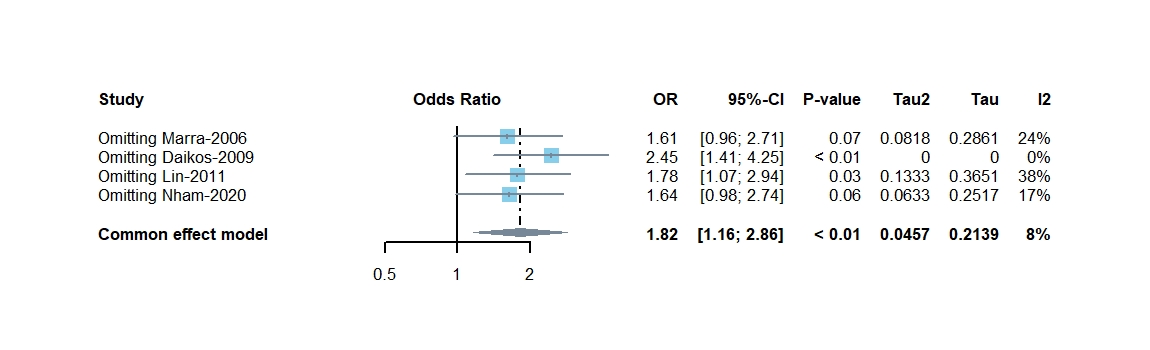


**Figure S18 Forest plot of 28- or 30-day mortality sensitivity test for ESBL vs non-ESBL KPB**


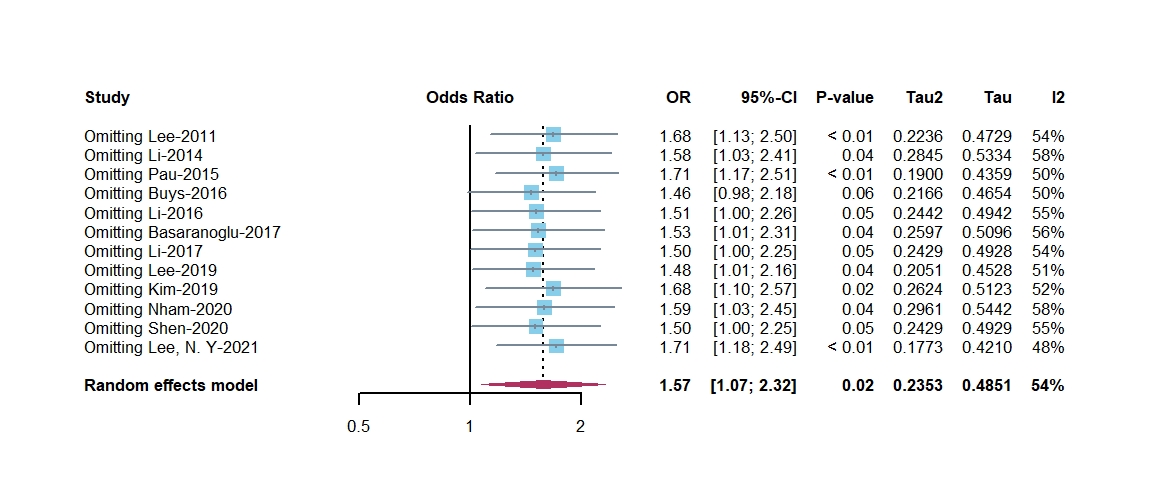


**Figure S19 Forest plot of in-hospital mortality sensitivity test for ESBL vs non-ESBL KPB**


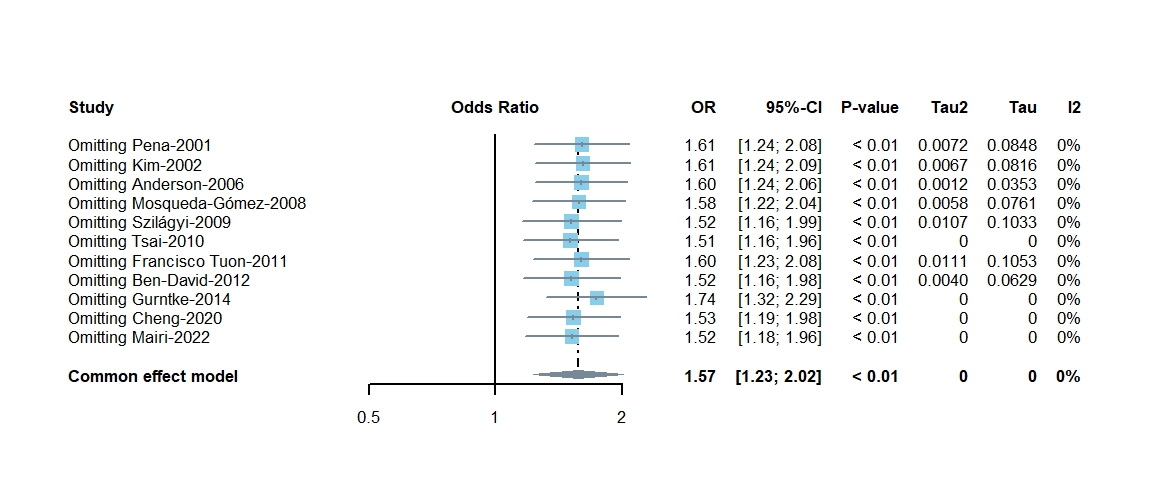


**Figure S20 Forest plot of 7-day mortality sensitivity test for CRKP vs non-CRKP KPB**


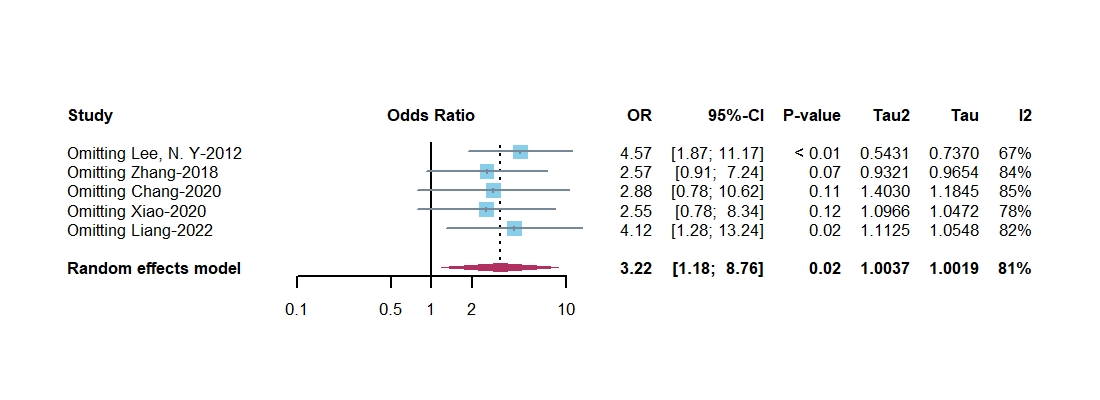


**Figure S21 Forest plot of 14-day mortality sensitivity test for CRKP vs non-CRKP KPB**


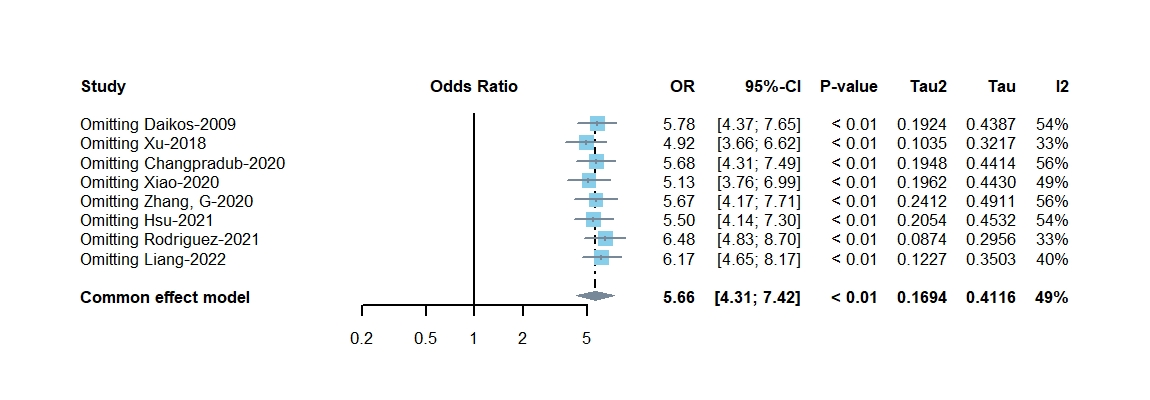


**Figure S22 Forest plot of 30-day mortality sensitivity test for CRKP vs non-CRKP KPB**


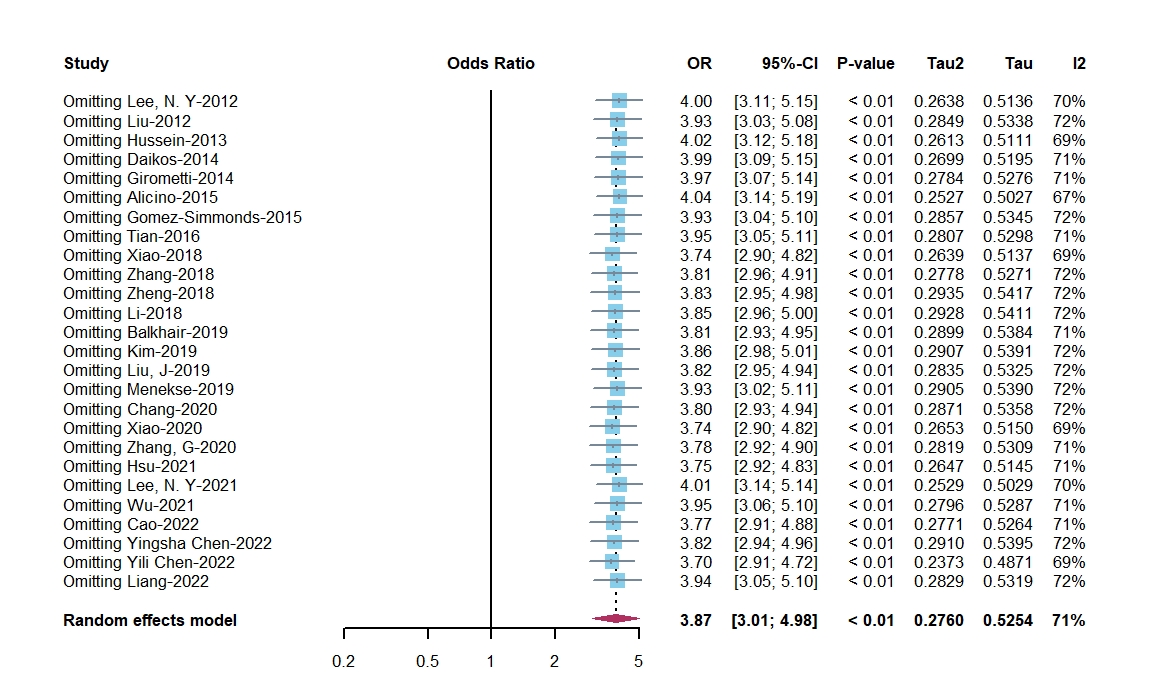


**Figure S23 Forest plot of in-hospital mortality sensitivity test for CRKP vs non-CRKP KPB**


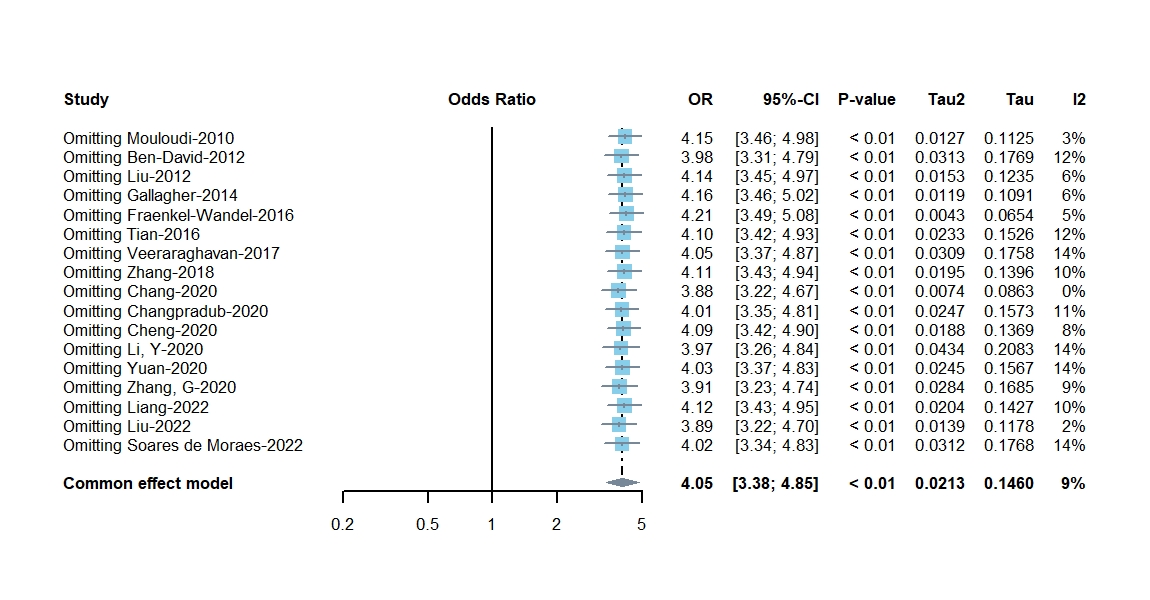


**Figure S24 Funnel plot of single proportion 7-day mortality for KPB**


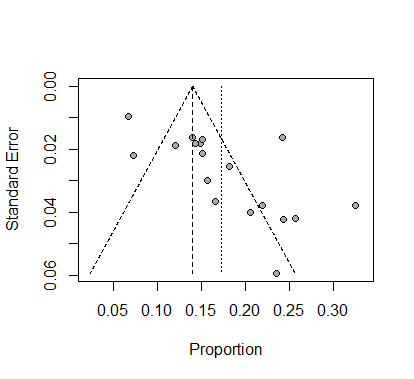


Peter’s test, linear regression test of funnel plot asymmetry; t = 1.25, df = 16, p-value = 0.2296.

**Figure S25 Funnel plot of single proportion 14-day mortality for KPB**


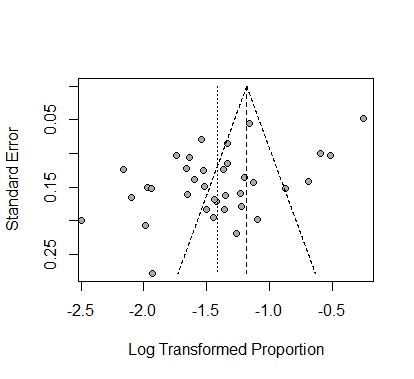


Peter’s test, linear regression test of funnel plot asymmetry; t = 2.74, df = 34, p-value = 0.0097.

**Figure S26 Funnel plot of single proportion 30-day mortality for KPB**

**
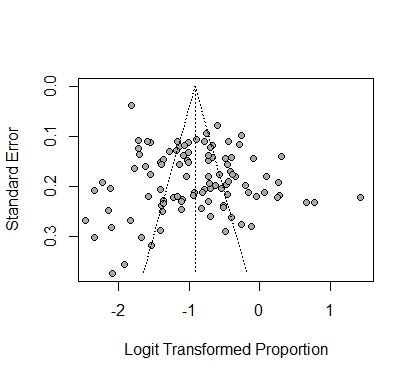
**

Peter’s test, linear regression test of funnel plot asymmetry; t = 5.54, df = 99, p-value < 0.0001.

**Figure S27 Funnel plot of single proportion 90-day mortality for KPB**


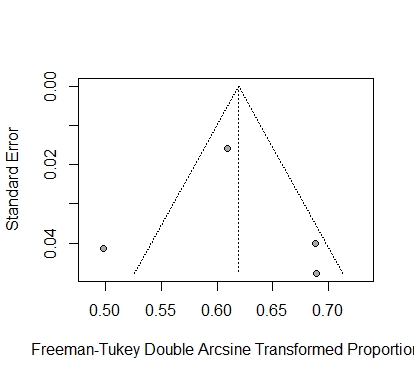


Too few studies to test for funnel plot asymmetry

**Figure S28 Funnel plot of single proportion in-hospital mortality for KPB**


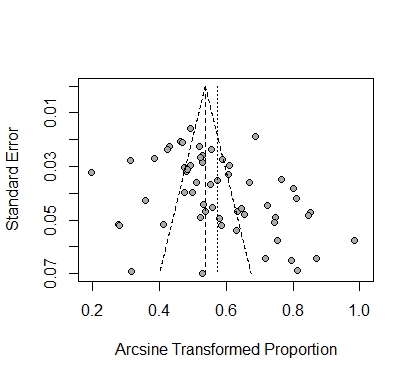


Peter’s test, linear regression test of funnel plot asymmetry; t = 3.89, df = 55, p-value = 0.0003.

**Figure S29 Funnel plot of 14-day mortality for ESBL vs non-ESBL KPB**


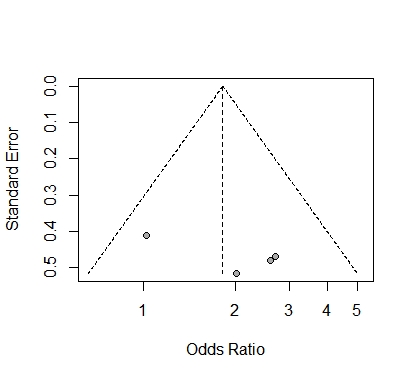


Too few studies to test for funnel plot asymmetry

**Figure S30 Funnel plot of 28- or 30-day mortality for ESBL vs non-ESBL KPB**


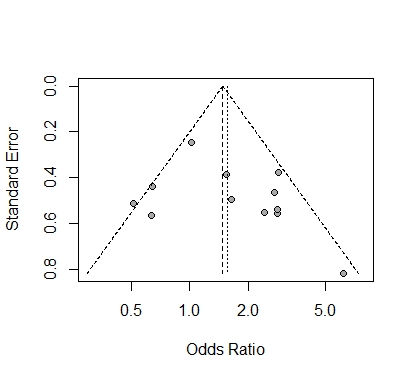


Peter’s test, linear regression test of funnel plot asymmetry; t = 0.84, df = 10, p-value = 0.4190.

**Figure S31 Funnel plot of in-hospital mortality for ESBL vs non-ESBL KPB**


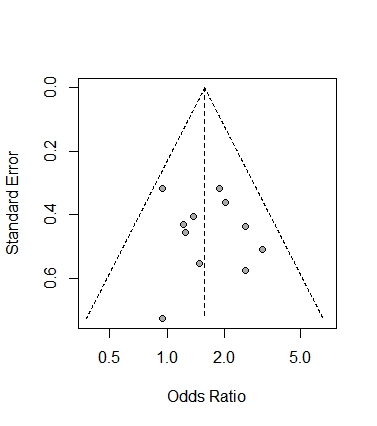


Peter’s test, linear regression test of funnel plot asymmetry; t = -0.41, df = 9, p-value = 0.6923.

**Figure S32 Funnel plot of 7-day mortality for CRKP vs non-CRKP KPB**


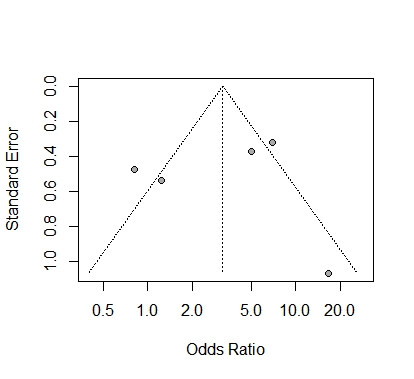


Too few studies to test for funnel plot asymmetry

**Figure S33 Funnel plot of 14-day mortality for CRKP vs non-CRKP KPB**


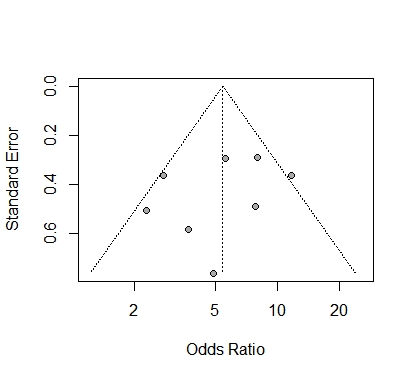


Too few studies to test for funnel plot asymmetry

**Figure S34 Funnel plot of 28- or 30-day mortality for CRKP vs non-CRKP KPB**


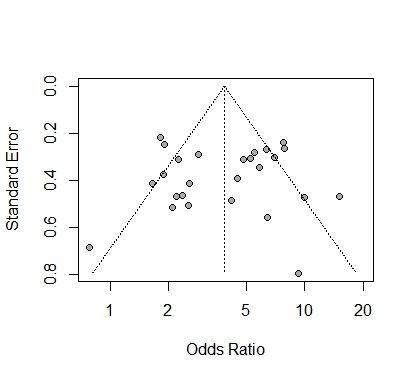


Peter’s test, linear regression test of funnel plot asymmetry; t = -1.21, df = 24, p-value = 0.2391

**Figure S35 Funnel plot of in-hospital mortality for CRKP vs non-CRKP KPB**


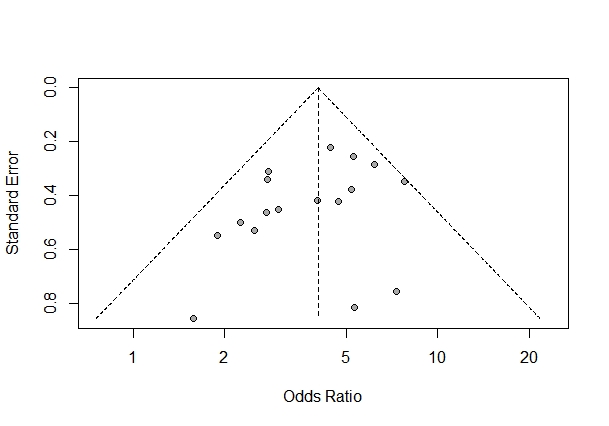


Peter’s test, linear regression test of funnel plot asymmetry; t = -2.04, df = 15, p-value = 0.0593.

**Figure S36 Funnel plot of single proportion 14-day mortality for KPB in trim and filled model**


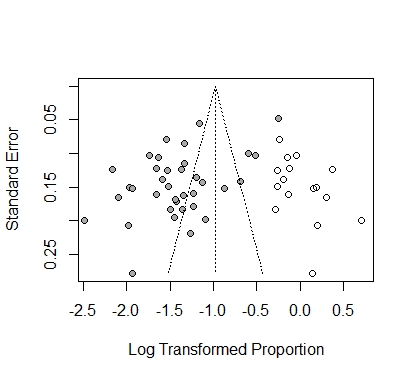


Random effects model

Adjusted proportion: 0.3760, 95%-CI [0.3034; 0.4661]

Heterogeneity:

tau^2 = 0.5999; I^2 = 97.0%; p-value < 0.001

**Figure S37 Funnel plot of single proportion 28- or 30-day mortality for KPB in trim and filled model**


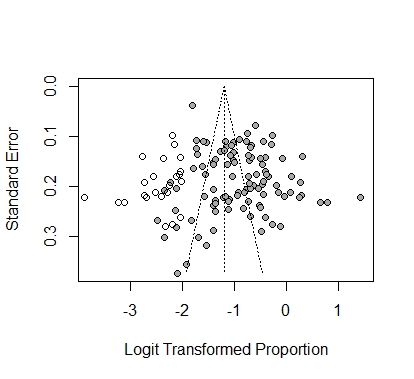


Random effects model

Adjusted proportion: 0.2316, 95%-CI [0.2046; 0.2610]

Heterogeneity:

tau^2 = 0.7717; I^2 = 96.1%; p-value < 0.001

**Figure S38 Funnel plot of single proportion in-hospital mortality for KPB in trim and filled model**


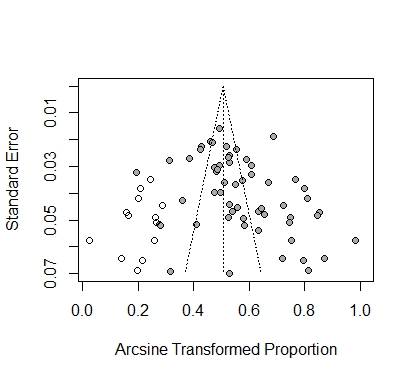


Random effects model

Adjusted proportion: 0.2349, 95%-CI [0.1950; 0.2773]

Heterogeneity:

tau^2 = 0.0411; I^2 = 95.3%; p-value < 0.0001
