# Supplementary material for: Klebsiella pneumoniae bacteremia mortality: a systematic review and meta-analysis
Source: Front Cell Infect Microbiol. 2023 Apr 20;13:1157010. doi: 10.3389/fcimb.2023.1157010 (PMC10159367; doi:10.3389/fcimb.2023.1157010)
Supplement: Supplementary file 2 [file Table_1.doc]

**Supplement Materies 1**

**Table of Contents**

| Table S1 PRISMA 2020 checklist | Page 2-4 |
| --- | --- |
| Text S1 Literature search strategy | Page 5 |
| Table S2 Select studies excluded at full-text review and reasons for exclusion | Page 6-20 |
| Table S3 Characteristics of the studies included in the meta-analysis | Page 21-38 |
| Text S2 References of all included studies | Page 39-54 |

**Table S1 PRISMA 2020 checklist**

| **Section and Topic** | **Item #** | **Checklist item** | **Location where item is reported** |
| --- | --- | --- | --- |
| **TITLE** | | |  |
| Title | 1 | Identify the report as a systematic review. | Page 1 |
| **ABSTRACT** | | |  |
| Abstract | 2 | See the PRISMA 2020 for Abstracts checklist. | Page 1-2 |
| **INTRODUCTION** | | |  |
| Rationale | 3 | Describe the rationale for the review in the context of existing knowledge. | Page 2-3 |
| Objectives | 4 | Provide an explicit statement of the objective(s) or question(s) the review addresses. | Page 2-3 |
| **METHODS** | | |  |
| Eligibility criteria | 5 | Specify the inclusion and exclusion criteria for the review and how studies were grouped for the syntheses. | Page 3-4 |
| Information sources | 6 | Specify all databases, registers, websites, organisations, reference lists and other sources searched or consulted to identify studies. Specify the date when each source was last searched or consulted. | Page 3 |
| Search strategy | 7 | Present the full search strategies for all databases, registers and websites, including any filters and limits used. | Supplementary Material Text 1 |
| Selection process | 8 | Specify the methods used to decide whether a study met the inclusion criteria of the review, including how many reviewers screened each record and each report retrieved, whether they worked independently, and if applicable, details of automation tools used in the process. | Page 4 |
| Data collection process | 9 | Specify the methods used to collect data from reports, including how many reviewers collected data from each report, whether they worked independently, any processes for obtaining or confirming data from study investigators, and if applicable, details of automation tools used in the process. | Page 4 |
| Data items | 10a | List and define all outcomes for which data were sought. Specify whether all results that were compatible with each outcome domain in each study were sought (e.g., for all measures, time points, analyses), and if not, the methods used to decide which results to collect. | Page 4-5 |
|  | 10b | List and define all other variables for which data were sought (e.g. participant and intervention characteristics, funding sources). Describe any assumptions made about any missing or unclear information. | Page 4 |
| Study risk of bias assessment | 11 | Specify the methods used to assess risk of bias in the included studies, including details of the tool(s) used, how many reviewers assessed each study and whether they worked independently, and if applicable, details of automation tools used in the process. | Page 4 |
| Effect measures | 12 | Specify for each outcome the effect measure(s) (e.g., risk ratio, mean difference) used in the synthesis or presentation of results. | Page 5 |
| Synthesis methods | 13a | Describe the processes used to decide which studies were eligible for each synthesis (e.g., tabulating the study intervention characteristics and comparing against the planned groups for each synthesis (item #5)). | Page 5 |
|  | 13b | Describe any methods required to prepare the data for presentation or synthesis, such as handling of missing summary statistics, or data conversions. | Page 5 |
|  | 13c | Describe any methods used to tabulate or visually display results of individual studies and syntheses. | Page 5 |
|  | 13d | Describe any methods used to synthesize results and provide a rationale for the choice(s). If meta-analysis was performed, describe the model(s), method(s) to identify the presence and extent of statistical heterogeneity, and software package(s) used. | Page 5 |
|  | 13e | Describe any methods used to explore possible causes of heterogeneity among study results (e.g., subgroup analysis, meta-regression). | Page 5 |
|  | 13f | Describe any sensitivity analyses conducted to assess robustness of the synthesized results. | Page 5 |
| Reporting bias assessment | 14 | Describe any methods used to assess risk of bias due to missing results in a synthesis (arising from reporting biases). | Page 5 |
| Certainty assessment | 15 | Describe any methods used to assess certainty (or confidence) in the body of evidence for an outcome. | Page 5 |
| **RESULTS** | | |  |
| Study selection | 16a | Describe the results of the search and selection process, from the number of records identified in the search to the number of studies included in the review, ideally using a flow diagram. | Figure 1 |
|  | 16b | Cite studies that might appear to meet the inclusion criteria, but which were excluded, and explain why they were excluded. | Supplementary Materials Table 2 |
| Study characteristics | 17 | Cite each included study and present its characteristics. | Supplementary Materials Text 2, Table 3 |
| Risk of bias in studies | 18 | Present assessments of risk of bias for each included study. | Supplementary Materials Figure 1 |
| Results of individual studies | 19 | For all outcomes, present, for each study: (a) summary statistics for each group (where appropriate) and (b) an effect estimate and its precision (e.g. confidence/credible interval), ideally using structured tables or plots. | Figure 2-3, Supplementary Materials Figure 2-23 |
| Results of syntheses | 20a | For each synthesis, briefly summarise the characteristics and risk of bias among contributing studies. | Page 6-7 |
|  | 20b | Present results of all statistical syntheses conducted. If meta-analysis was done, present for each the summary estimate and its precision (e.g. confidence/credible interval) and measures of statistical heterogeneity. If comparing groups, describe the direction of the effect. | Page 6-7, Table 2-3, |
|  | 20c | Present results of all investigations of possible causes of heterogeneity among study results. | Page 7 |
|  | 20d | Present results of all sensitivity analyses conducted to assess the robustness of the synthesized results. | Page 7 |
| Reporting biases | 21 | Present assessments of risk of bias due to missing results (arising from reporting biases) for each synthesis assessed. | Page 7, Supplementary Materials Figure 24-38 |
| Certainty of evidence | 22 | Present assessments of certainty (or confidence) in the body of evidence for each outcome assessed. | Not applicable |
| **DISCUSSION** | | |  |
| Discussion | 23a | Provide a general interpretation of the results in the context of other evidence. | Page 8 |
|  | 23b | Discuss any limitations of the evidence included in the review. | Page 9 |
|  | 23c | Discuss any limitations of the review processes used. | Page 9 |
|  | 23d | Discuss implications of the results for practice, policy, and future research. | Page 9 |
| **OTHER INFORMATION** | | |  |
| Registration and protocol | 24a | Provide registration information for the review, including register name and registration number, or state that the review was not registered. | Not applicable |
|  | 24b | Indicate where the review protocol can be accessed, or state that a protocol was not prepared. | Not applicable |
|  | 24c | Describe and explain any amendments to information provided at registration or in the protocol. | Not applicable |
| Support | 25 | Describe sources of financial or non-financial support for the review, and the role of the funders or sponsors in the review. | Page 10 |
| Competing interests | 26 | Declare any competing interests of review authors. | Page 9 |
| Availability of data, code and other materials | 27 | Report which of the following are publicly available and where they can be found template data collection forms; data extracted from included studies; data used for all analyses; analytic code; any other materials used in the review. | Page 10 |

**Text S1 Literature search strategy**

Search strategy:

To maximize the number of papers found, both medical subject headings and free text terms were used in the search strategy. The first search was performed until 14 April 2022. We updated searches to 18 September 2022.

**Pubmed**

((*Klebsiella pneumoniae)* AND (bacteremia or bloodstream infection)) AND (mortality or death or survival or outcome)

**Embase**

('klebsiella'/exp OR klebsiella) AND pneumoniae AND ('bacteremia'/exp OR bacteremia OR bloodstream) AND ('infection'/exp OR infection) AND ('mortality'/exp OR mortality OR 'death'/exp OR death OR 'survival'/exp OR survival OR 'outcome'/exp OR outcome)

**Web of science**

((*Klebsiella pneumoniae)* AND (bacteremia or bloodstream infection)) AND (mortality or death or survival or outcome) all fields

**Cochrane**

((*Klebsiella pneumoniae)* AND (bacteremia or bloodstream infection)) AND (mortality or death or survival or outcome)

**Table S2 Select studies excluded at full text review and reasons for exclusion (listed by publishing year)**

|  | Reference | Reason for exclusion |
| --- | --- | --- |
| 1 | García de la Torre, M., et al. (1985). "Klebsiella bacteremia: an analysis of 100 episodes." Rev Infect Dis **7**(2): 143-150 | No mortality data based on the results in the paper |
| 2 | Bonadio, W. A. (1989). "*Klebsiella pneumoniae* bacteremia in children. Fifty-seven cases in 10 years." Am J Dis Child **143**(9): 1061-1063 | Case series |
| 3 | Wang, L. S., et al. (1990). "*Klebsiella pneumoniae* bacteremia: analysis of 100 episodes." J Formos Med Assoc **89**(9): 756-763 | subgroup, including 25% *Klebsiella oxytoca* |
| 4 | Yinnon, A. M., et al. (1996). "Klebsiella bacteraemia: community versus nosocomial infection." Qjm **89**(12): 933-941 | Subgroup, include 13% *Klebsiella oxytoca* |
| 5 | Hansen, D. S., et al. (1998). "Epidemiology of Klebsiella bacteraemia: a case control study using Escherichia coli bacteraemia as control." J Hosp Infect **38**(2): 119-132 | Include 24% *Klebsiella oxytoca* |
| 6 | Oral, R., et al. (1998). "Neonatal Klebsiella pneumonia sepsis and imipenem/cilastatin." Indian J Pediatr **65**(1): 121-129. | Include cerebrospinal fluid samples |
| 7 | Ariffin, H., et al. (2000). "Ceftazidime-resistant *Klebsiella pneumoniae* bloodstream infection in children with febrile neutropenia." International journal of infectious diseases: IJID: official publication of the International Society for Infectious Diseases **4**(1): 21-25 | N=29 |
| 8 | Martinez-Aguilar, G., et al. (2001). "Outbreak of nosocomial sepsis and pneumonia in a newborn intensive care unit by multiresistant extended-spectrum beta-lactamase-producing *Klebsiella pneumoniae:* high impact on mortality." Infect Control Hosp Epidemiol **22**(11): 725-728 | N=18 |
| 9 | Yang, P. Y., et al. (2001). "*Klebsiella pneumoniae* bacteremia: community-acquired vs. nosocomial infections." Chang Gung Med J **24**(11): 688-696 | The full text was not available |
| 10 | Yoon, C. J., et al. (2001). "Extended spectrum beta-lactamase (ESBL) producing *Klebsiella pneumoniae* bacteremia: Risk factors and outcome." Clinical Infectious Diseases **33**(7): 1104-1104. | The full text was not available |
| 11 | Du, B., et al. (2002). "Extended-spectrum beta-lactamase-producing Escherichia coli and *Klebsiella pneumoniae* bloodstream infection: risk factors and clinical outcome." Intensive Care Med **28**(12): 1718-1723 | Subgroup, n=25 |
| 12 | Krontal, S., et al. (2002). "Klebsiella bacteremia in children in southern Israel (1988-1997)." Infection **30**(3): 125-131 | There were ten episodes due to *Klebsiella oxytoca*. |
| 13 | Chang, M. and B. A. Cunha (2004). "*Klebsiella pneumoniae* bacteremia associated with a Tesio hemodialysis catheter." Am J Infect Control **32**(6): 374 | Case report |
| 14 | Endimiani, A., et al. (2004). "Bacteremia due to *Klebsiella pneumoniae* isolates producing the TEM-52 extended-spectrum beta-lactamase: Treatment outcome of patients receiving imipenem or ciprofloxacin." Clinical Infectious Diseases **38**(2): 243-251 | N=35 |
| 15 | Kang, C. I., et al. (2004). "Risk factors for and clinical outcomes of bloodstream infections caused by extended-spectrum beta-lactamase-producing *Klebsiella pneumoniae.*" Infect Control Hosp Epidemiol **25**(10): 860-867 | Same data described in with Kang 2006, J Korean Med Sci **21**(5): 816-822 |
| 16 | Pai, H., et al. (2004). "Epidemiology and clinical features of bloodstream infections caused by AmpC-type-beta-lactamase-producing *Klebsiella pneumoniae.*" Antimicrob Agents Chemother **48**(10): 3720-3728 | Secondary analysis, same data described in Kang 2004 Infect Control Hosp Epidemiol **25**(10): 860-867 |
| 17 | Bhavnani, S. M., et al. (2006). "Outcomes evaluation of patients with ESBL- and non-ESBL-producing Escherichia coli and Klebsiella species as defined by CLSI reference methods: report from the SENTRY Antimicrobial Surveillance Program." Diagn Microbiol Infect Dis **54**(3): 231-236 | Subgroup, no KPB mortality data |
| 18 | Lee, C. H., et al. (2006). "Treatment of ESBL-producing *Klebsiella pneumoniae* bacteraemia with carbapenems or flomoxef: a retrospective study and laboratory analysis of the isolates." J Antimicrob Chemother **58**(5): 1074-1077 | N=27 |
| 19 | Niu, M. T., et al. (2006). "Transfusion-transmitted *Klebsiella pneumoniae* fatalities, 1995 to 2004." Transfus Med Rev **20**(2): 149-157 | Case repoart |
| 20 | Harish, B. N., et al. (2007). "Extended-spectrum beta-lactamase-producing *Klebsiella pneumoniae* from blood culture." J Med Microbiol **56**(Pt 7): 999-1000 | Mortality data was not reported |
| 21 | Vandijck, D. M., et al. (2007). "Community-acquired versus nosocomial Klebsiella pneumonia bacteremia: clinical features, treatment outcomes, and clinical implication of antimicrobial resistance." J Korean Med Sci **22**(4): 770-771 | Correspondence |
| 22 | Cordery, R. J., et al. (2008). "Evaluation of risk factors for the acquisition of bloodstream infections with extended-spectrum beta-lactamase-producing Escherichia coli and Klebsiella species in the intensive care unit; antibiotic management and clinical outcome." Journal of Hospital Infection **68**(2): 108-115 | N<50 |
| 23 | Thom, K. A., et al. (2008). "Impact of empiric antimicrobial therapy on outcomes in patients with Escherichia coli and *Klebsiella pneumoniae* bacteremia: a cohort study." BMC Infect Dis **8**: 116 | Include 28 *K. oxytoca*, 2 non-speciated *Klebsiella* isolates |
| 24 | Vasilev, K., et al. (2008). "A Phase 3, open-label, non-comparative study of tigecycline in the treatment of patients with selected serious infections due to resistant Gram-negative organisms including Enterobacter species, Acinetobacter baumannii and *Klebsiella pneumoniae.*" J Antimicrob Chemother **62 Suppl 1**: i29-40. | N=36 |
| 25 | Borer, A., et al. (2009). "Attributable mortality rate for carbapenem-resistant *Klebsiella pneumoniae* bacteremia." Infect Control Hosp Epidemiol **30**(10): 972-976 | N=32 |
| 26 | Lee, C. I., et al. (2009). "Extended-spectrum beta-lactamase-producing phenotype signifies a poor prognosis for patients with cefpodoxime-resistant Escherichia coli or *Klebsiella pneumoniae* bacteremia." J Microbiol Immunol Infect **42**(4): 303-309 | Subgroup, no KPB mortality data |
| 27 | Meatherall, B., et al. (2009). "*Klebsiella pneumoniae* bacteremia in a large Canadian region." International Journal of Antimicrobial Agents **34**: S113-S114 | Same data with Meatherall 2009 Am J Med **122**(9): 866-873 |
| 28 | Petrikkos, P., et al. (2009). "Prospective study of *Klebsiella pneumoniae* bacteremia: Risk factors and clinical significance of type VIM-1 metallo-beta-lactamases." Archives of Hellenic Medicine **26**(3): 374-383 | Same data described in Daikos 2009 Antimicrob Agents Chemother **53**(5): 1868-1873. |
| 29 | Superti, S. V., et al. (2009). "Risk factors for and mortality of extended-spectrum-beta-lactamase-producing *Klebsiella pneumoniae* and Escherichia coli nosocomial bloodstream infections." Rev Inst Med Trop Sao Paulo **51**(4): 211-216. | No KPB mortality data |
| 30 | Yang, Y. S., et al. (2009). "Community-onset bacteremic urinary tract infections caused by extended-spectrum beta-lactamase-producing Escherichia coli and *Klebsiella pneumoniae.*" Journal of Medical Sciences **29**(5): 265-268. | No KPB mortality data |
| 31 | Al-Hasan, M. N., et al. (2010). "Epidemiology and Outcome of Klebsiella Species Bloodstream Infection: A Population-Based Study." Mayo Clin Proc **85**(2): 139-144 | No KPB mortality data |
| 32 | Nguyen, M., et al. (2010). "Carbapenem-resistant *Klebsiella pneumoniae* bacteremia: factors correlated with clinical and microbiologic outcomes." Diagn Microbiol Infect Dis **67**(2): 180-184 | N=48 |
| 33 | Vardakas, K., et al. (2010). "Characteristics and outcomes of intensive care unit patients with carbapenem-resistant *Klebsiella pneumoniae* bacteraemia." Clinical Microbiology and Infection **16**: S358 | Conference abstract |
| 34 | Yang, Y. S., et al. (2010). "Impact of Extended-spectrum β-lactamase-producing Escherichia coli and *Klebsiella pneumoniae* on the outcome of community-onset bacteremic urinary tract infections." J Microbiol Immunol Infect **43**(3): 194-199 | N=23 |
| 35 | Ferrández Quirante, O., et al. (2011). "Risk factors for bloodstream infections caused by extended-spectrum β-lactamase-producing Escherichia coli and *Klebsiella pneumoniae.*" Brazilian Journal of Infectious Diseases **15**(4): 370-376 | Subgroup, no KPB mortality data |
| 36 | Hussein, K., et al. (2011). "Hospital-acquired *Klebsiella pneumoniae* bacteraemia: Risk factors for carbapenem-resistance and predictor for mortality." Clinical Microbiology and Infection **17**: S479 | Conference abstract |
| 37 | Kang, C. I., et al. (2011). "Risk factors for infection and treatment outcome of extended-spectrum β-lactamase-producing Escherichia coli and *Klebsiella pneumoniae* bacteremia in patients with hematologic malignancy." Annals of Hematology: 1-7 | Subgroup, cannot derive the number of patients who died with KPB |
| 38 | Lee, N. Y., et al. (2011). "Carbapenem therapy for bacteremia due to extended-spectrum β-lactamase-producing Escherichia coli or *Klebsiella pneumoniae.*" Diagn Microbiol Infect Dis **70**(1): 150-153 | Subgroup, cannot derive the number of patients who died with KPB |
| 39 | Liao, C. H., et al. (2011). "*Klebsiella pneumoniae* bacteremia and capsular serotypes, Taiwan." Emerg Infect Dis **17**(6): 1113-1115 | Same data described in Liao, 2009 Antimicrob Agents Chemother **53**(5): 1868-1873 |
| 40 | Orfanidou, M., et al. (2011). "Clinical and laboratory investigation of KPC-2 *Klebsiella pneumoniae* bacteraemia in patients of a haematology ward." Clinical Microbiology and Infection **17**: S811 | N=12 |
| 41 | Ortega, M., et al. (2011). "Cefotaxime resistance and outcome of Klebsiella spp bloodstream infection." European Journal of Clinical Microbiology & Infectious Diseases **30**(12): 1599-1605 | Subgroup, cannot derive the number of patients who died with KPB |
| 42 | Quirante, O. F., et al. (2011). "Risk factors for bloodstream infections caused by extended-spectrum beta-lactamase-producing Escherichia coli and *Klebsiella pneumoniae.*" Brazilian Journal of Infectious Diseases **15**(4): 370-376 | Subgroup, cannot derive the number of patients who died with KPB |
| 43 | Wang, S. S., et al. (2011). "Clinical manifestations and prognostic factors in cancer patients with bacteremia due to extended-spectrum β-lactamase-producing Escherichia coli or *Klebsiella pneumoniae.*" J Microbiol Immunol Infect **44**(4): 282-288 | Subgroup, cannot derive the number of patients who died with KPB |
| 44 | Chopra, T., et al. (2012). "Impact of cefepime therapy on mortality among patients with bloodstream infections caused by extended-spectrum-β-lactamase-producing *Klebsiella pneumoniae* and Escherichia coli." Antimicrob Agents Chemother **56**(7): 3936-3942 | Subgroup, insufficient information to evaluate risk of bias |
| 45 | Hsueh, P. R. and W. C. Ko (2012). "Carbapenem therapy for bacteremia due to extended-spectrum β-lactamase-producing Escherichia coli or *Klebsiella pneumoniae:* Implications of Ertapenem susceptibility." International Journal of Infectious Diseases **16**: e440 | Conference Abstract |
| 46 | Lee, N. Y., et al. (2012). "Carbapenem therapy for bacteremia due to extended-spectrum-β-lactamase-producing Escherichia coli or *Klebsiella pneumoniae:* implications of ertapenem susceptibility." Antimicrob Agents Chemother **56**(6): 2888-2893. | Subgroup, cannot derive the number of patients who died with KPB |
| 47 | Maherault, A. C., et al. (2012). "Efficacy of imipenem for the treatment of bacteremia due to an OXA-48-producing *Klebsiella pneumoniae* isolate." Clin Infect Dis **54**(4): 577-578. | Correspondence, case report |
| 48 | Matsumura, Y., et al. (2012). "Cefotaxime for the detection of extended-spectrum β-lactamase or plasmid-mediated AmpC β-lactamase and clinical characteristics of cefotaxime-non-susceptible Escherichia coli and *Klebsiella pneumoniae* bacteraemia." Eur J Clin Microbiol Infect Dis **31**(8): 1931-1939. | Subgroup, cannot derive the number of patients who died with KPB |
| 49 | Medic, D., et al. (2012). "BLOODSTREAM INFECTIONS IN CHILDREN CAUSED BY EXTENDED SPECTRUM BETA-LACTAMASE-PRODUCING *KLEBSIELLA PNEUMONIAE.*" Archives of Biological Sciences **64**(4): 1339-1347. | The KPB mortality data did not reported |
| 50 | Papaioannou, V., et al. (2012). "Bloodstream infections due to carbapenem-resistant *Klebsiella pneumoniae,* Pseudomonas aeruginosa and Acinetobacter baumannii clinical isolates in a tertiary hospital: Epidemiology and outcome." Clinical Microbiology and Infection **18**: 763. | Conference abstract |
| 51 | Paramythiotou, E. E., et al. (2012). "Analysis of all bacteremia episodes caused by carbapenemase-producing klebsiella in an intensive care unit (ICU)." Intensive Care Med **38**: S82-S83 | N=23 |
| 52 | Qureshi, Z. A., et al. (2012). "Treatment outcome of bacteremia due to KPC-producing *Klebsiella pneumoniae:* superiority of combination antimicrobial regimens." Antimicrob Agents Chemother **56**(4): 2108-2113 | N=41 |
| 53 | Rose, C., et al. (2012). "A cohort study of patients with klebsiella bacteremia with carbapenem resistance compared to those with third-generation cephalosporin resistance." Crit Care Med **40**(12): 84 | Conference abstract |
| 54 | Tsaousi, S., et al. (2012). "Observational study of bloodstream infections caused by carbapenemase-producing *Klebsiella pneumoniae.*" Clinical Microbiology and Infection **18**: 97. | Conference abstract |
| 55 | Tsui, K., et al. (2012). "Laboratory identification, risk factors, and clinical outcomes of patients with bacteremia due to Escherichia coli and *Klebsiella pneumoniae* producing extended-spectrum and AmpC type β-lactamases." J Microbiol Immunol Infect **45**(3): 193-199. | Subgroup, cannot derive the number of patients who died with KPB |
| 56 | Tuon, F. F., et al. (2012). "Risk factors for KPC-producing *Klebsiella pneumoniae* bacteremia." Braz J Infect Dis **16**(5): 416-419. | Mortality not reported |
| 57 | Yang, C. C., et al. (2012). "Discrepancy between effects of carbapenems and flomoxef in treating nosocomial hemodialysis access-related bacteremia secondary to extended spectrum beta-lactamase producing *Klebsiella pneumoniae* in patients on maintenance hemodialysis." BMC Infect Dis **12**: 206. | Same data described in Yang 2014 International journal of infectious diseases: IJID: official publication of the International Society for Infectious Diseases **28**: 3-7 |
| 58 | Adrianzén, D., et al. (2013). "Mortality caused by bacteremia Escherichia coli and Klebsiella spp. Extended-spectrum beta-lactamase-producers: A retrospective cohort from a hospital in Lima, Peru." Revista Peruana de Medicina Experimental y Salud Publica **30**(1): 18-25. | Subgroup, cannot derive the number of patients who died with KPB |
| 59 | Cober, E., et al. (2013). "Impact of carbapenem resistance in *Klebsiella pneumoniae* blood stream infection in solid organ transplantation." American Journal of Transplantation **13**: 186 | Conference abstract |
| 60 | Liao, C. H., et al. (2013). "Risk factors and clinical characteristics of patients with qnr-positive *Klebsiella pneumoniae* bacteraemia." J Antimicrob Chemother **68**(12): 2907-2914. | Same data described in Liao 2009 Clinical microbiology and infection: the official publication of the European Society of Clinical Microbiology and Infectious Diseases 15(12): 1119-1125 |
| 61 | Viale, P., et al. (2013). "Predictors of mortality in multidrug-resistant *Klebsiella pneumoniae* bloodstream infections." Expert review of anti-infective therapy **11**(10): 1053-1063 | review |
| 62 | Balcı, U., et al. (2014). "Risk factors and mortality of bloodstream infections caused by extended-spectrum β-Lactamase-positive escherichia coli and *Klebsiella pneumoniae.*" Klimik Dergisi **27**(1): 15-20 | Subgroup, cannot derive the number of patients who died with KPB |
| 63 | Corcione, S., et al. (2014). "Healthcare-associated *Klebsiella pneumoniae* carbapenemase producing K. pneumoniae bloodstream infection: the time has come." Clin Infect Dis **59**(2): 321-322 | N=18 |
| 64 | Fligou F, Papadimitriou-Olivgeris M, Sklavou C, Anastassiou ED, Marangos  M, Filos K. Risk factors and predictors of mortality for KPC-producing  *Klebsiella pneumoniae* bactereamia during intensive care unit stay. Eur J  Anaesthesiology. 2013;30(suppl 51):187 | Only abstract |
| 65 | Giuliano, S., et al. (2014). "Severe community onset healthcare-associated Clostridium difficile infection complicated by carbapenemase producing *Klebsiella pneumoniae* bloodstream infection." Bmc Infectious Diseases **14**(1) | Case report |
| 66 | Ku, N. S., et al. (2014). "Risk factors for 28-day mortality in elderly patients with extended-spectrum β-lactamase (ESBL)-producing Escherichia coli and *Klebsiella pneumoniae* bacteremia." Arch Gerontol Geriatr **58**(1): 105-109 | Subgroup, insufficient information to evaluate risk of bias |
| 67 | Leistner, R., et al. (2014). "Bloodstream infection due to extended-spectrum beta-lactamase (ESBL)-positive K. pneumoniae and E. coli: an analysis of the disease burden in a large cohort." Infection **42**(6): 991-997 | Same data described in Gurntke, 2014 Journal of Infection and Chemotherapy 20(12): 817-819 |
| 68 | Lin, Y. T., et al. (2014). "Clinical and microbiological characteristics of tigecycline non-susceptible *Klebsiella pneumoniae* bacteremia in Taiwan." BMC Infect Dis **14**: 1 | N=36 |
| 69 | Mouloudi, E., et al. (2014). "Bloodstream infections caused by carbapenemase-producing *Klebsiella pneumoniae* among intensive care unit patients after orthotopic liver transplantation: risk factors for infection and impact of resistance on outcomes." Transplant Proc **46**(9): 3216-3218 | N=17 |
| 70 | Oliva, A., et al. (2014). "Synergistic activity and effectiveness of a double-carbapenem regimen in pandrug-resistant *Klebsiella pneumoniae* bloodstream infections." J Antimicrob Chemother **69**(6): 1718-1720 | Case report |
| 71 | Perianes-Diaz, M. E., et al. (2014). "Bacteremia caused by Escherichia coli and *Klebsiella pneumoniae* producing extended-spectrum betalactamases: Mortality and readmission-related factors." Med Clin (Barc) **142**(9): 381-386 | N=18 |
| 72 | Amit, S., et al. (2015). "Bloodstream infections among carriers of carbapenem-resistant *Klebsiella pneumoniae:* etiology, incidence and predictors." Clinical microbiology and infection: the official publication of the European Society of Clinical Microbiology and Infectious Diseases **21**(1): 30-34 | N=19 |
| 73 | Biehle, L. R., et al. (2015). "Outcomes and Risk Factors for Mortality among Patients Treated with Carbapenems for Klebsiella spp. Bacteremia." PLoS One **10**(11) | Include *K. oxytoca* |
| 74 | Chopra, T., et al. (2015). "Risk factors for bloodstream infection caused by extended-spectrum β-lactamase-producing Escherichia coli and *Klebsiella pneumoniae:* A focus on antimicrobials including cefepime." Am J Infect Control **43**(7): 719-723 | Subgroup, cannot derive the number of patients who died with KPB |
| 75 | Foresti, S., et al. (2015). "Tigecycline Lock Therapy for Catheter-Related Bloodstream Infection Caused by KPC-Producing *Klebsiella pneumoniae* in Two Pediatric Hematological Patients." Antimicrob Agents Chemother **59**(12): 7919-7920 | Case report |
| 76 | Han, S. B., et al. (2015). "Extended-spectrum β-lactamase-producing Escherichia coli and *Klebsiella pneumoniae* bacteremia in febrile neutropenic children." Microb Drug Resist **21**(2): 244-251 | N=32 |
| 77 | Harris, P. N., et al. (2015). "Comparable outcomes for β-lactam/β-lactamase inhibitor combinations and carbapenems in definitive treatment of bloodstream infections caused by cefotaxime-resistant Escherichia coli or *Klebsiella pneumoniae.*" Antimicrob Resist Infect Control **4**: 14. | Subgroup, cannot derive the number of patients who died with KPB |
| 78 | Kyle, J. M., et al. (2015). "Fosfomycin for Multidrug Treatment of *Klebsiella pneumoniae* Carbapenemase Bacteremia." Annals of Pharmacotherapy **49**(3): 366-367 | Case report |
| 79 | La, E. and T. L. Doan (2015). "Evaluating the appropriateness of empiric and targeted therapy in the treatment of *Klebsiella pneumoniae* bacteremia." Pharmacotherapy **35**(11): e196 | Conference abstract |
| 80 | Lo, C. L., et al. (2015). "Role of cefepime therapy for carbapenem-resistant *Klebsiella pneumoniae* bloodstream infections." Journal of Microbiology, Immunology and Infection **48**(2): S39-S40 | Conference abstract |
| 81 | Nguyen, M. L., et al. (2015). "Risk factors for and outcomes of bacteremia caused by extended-spectrum β-lactamase- producing Escherichia coli and klebsiella species at a canadian tertiary care hospital." Canadian Journal of Hospital Pharmacy **68**(2): 136-143 | Subgroup, cannot derive the number of patients who died with KPB |
| 82 | Shields, R. K., et al. (2015). "Doripenem MICs and ompK36 Porin Genotypes of Sequence Type 258, KPC-Producing *Klebsiella pneumoniae* May Predict Responses to Carbapenem-Colistin Combination Therapy among Patients with Bacteremia." Antimicrob Agents Chemother **59**(3): 1505-1509 | N=27 |
| 83 | Trecarichi, E. M., et al. (2015). "Bloodstream infections caused by *Klebsiella pneumoniae* in onco-hematological patients: Incidence and clinical impact of carbapenem resistance in a multicentre prospective survey." Blood **126**(23): 3757 | Conference abstract |
| 84 | Zhao, F., et al. (2015). "Dissemination of extensively drug-resistant and KPC-2 producing *Klebsiella pneumoniae* isolated from bloodstream infections." J Infect Dev Ctries **9**(9): 1016-1021 | Case series, N=24 |
| 85 | Baker, T., et al. (2016). "Characterization of piperacillin-tazobactam-non-susceptible, but ceftriaxone-susceptible, escherichia coli and *Klebsiella pneumoniae* bloodstream infections." Open Forum Infectious Diseases **3** | Conference abstract |
| 86 | Giacobbe, D. R., et al. (2016). "Comment on: Mortality due to blaKPC *Klebsiella pneumoniae* bacteraemia." J Antimicrob Chemother **71**(6): 1743-1744 | Comment |
| 87 | Leistner, R., et al. (2016). "E. coli bacteremia in comparison to K. pneumoniae bacteremia: Influence of pathogen species and ESBL production on 7-day mortality." Antimicrobial Resistance and Infection Control **5**(1) | Subgroup, cannot derive the number of patients who died with KPB |
| 88 | Raju, M. S., et al. (2016). "Continuous evaluation of changes in the serum proteome from early to late stages of sepsis caused by *Klebsiella pneumoniae.*" Mol Med Rep **13**(6): 4835-4844 | N=12 |
| 89 | Sakellariou, C., et al. (2016). "Sepsis Caused by Extended-Spectrum Beta-Lactamase (ESBL)-Positive K-pneumoniae and E-coli: Comparison of Severity of Sepsis, Delay of Anti-Infective Therapy and ESBL Genotype." PLoS One **11**(7) | Subgroup, insufficient information to evaluate risk of bias |
| 90 | Shields, R. K., et al. (2016). "Aminoglycosides for Treatment of Bacteremia Due to Carbapenem-Resistant *Klebsiella pneumoniae.*" Antimicrob Agents Chemother **60**(5): 3187-3192 | N=36 |
| 91 | Shimizu, A., et al. (2016). "Comparison of bacterial and clinical features of *Klebsiella pneumoniae* and klebsiella oxytoca bloodstream infections: Experience at a Tertiary Hospital In Japan." Open Forum Infectious Diseases **3** | Conference abstract |
| 92 | Wiener-Well, Y., et al. (2016). "Mortality due to blaKPC *Klebsiella pneumoniae* bacteraemia-authors' response." J Antimicrob Chemother **71**(6): 1744 | Response |
| 93 | Bi, W., et al. (2017). "Extensively Drug-Resistant *Klebsiella pneumoniae* Causing Nosocomial Bloodstream Infections in China: Molecular Investigation of Antibiotic Resistance Determinants, Informing Therapy, and Clinical Outcomes." Front Microbiol **8**: 1230 | N=35 |
| 94 | Forcina, A., et al. (2017). "Control of infectious mortality due to carbapenemase-producing *Klebsiella pneumoniae* in hematopoietic stem cell transplantation." Bone Marrow Transplant **52**(1): 114-119 | N=14 |
| 95 | Giacobbe, D. R., et al. (2017). "Previous bloodstream infections due to other pathogens as predictors of carbapenem-resistant *Klebsiella pneumoniae* bacteraemia in colonized patients: results from a retrospective multicentre study." Eur J Clin Microbiol Infect Dis **36**(4): 663-669 | N=37 |
| 96 | Giuliano, G., et al. (2017). "Predictors of mortality in KPC-KP bloodstream infection in intensive care units in Italy." Critical Care **21**(1) | N=42 |
| 97 | Joo, E. J., et al. (2017). "Impact of appropriateness of empiric therapy on outcomes in community-onset bacteremia by extended-spectrum-β-lactamase producing Escherichia coli and Klebisella pneumoniae definitively treated with carbapenems." Eur J Clin Microbiol Infect Dis **36**(11): 2093-2100 | Subgroup, cannot derive the number of patients who died with KPB |
| 98 | Kaur, A., et al. (2017). "Clinical outcome of pan-drug resistant *Klebsiella pneumoniae* (PDRKP) bloodstream infections-a single center study in India." Antimicrobial Resistance and Infection Control **6** | Same data described in Kaur, 2017 Am J Infect Control 45(11): 1289-1291 |
| 99 | Lo, C. L., et al. (2017). "Fluoroquinolone therapy for bloodstream infections caused by extended-spectrum beta-lactamase-producing Escherichia coli and *Klebsiella pneumoniae.*" J Microbiol Immunol Infect **50**(3): 355-361 | Subgroup, cannot derive the number of patients who died with KPB |
| 100 | Man, M., et al. (2017). "Clinical predictors and outcome of *Klebsiella pneumoniae* bacteremia in a regional hospital in hong kong." Critical Care **21**(1) | Conference abstract |
| 101 | Micozzi, A., et al. (2017). "Carbapenem-resistant *Klebsiella pneumoniae* in high-risk haematological patients: factors favouring spread, risk factors and outcome of carbapenem-resistant *Klebsiella pneumoniae* bacteremias." Bmc Infectious Diseases **17** | N=22 |
| 102 | Papadimitriou-Olivgeris, M., et al. (2017). "Early KPC-Producing *Klebsiella pneumoniae* Bacteremia among Intensive Care Unit Patients Non-Colonized upon Admission." Pol J Microbiol **66**(2): 251-254 | N=24 |
| 103 | Quan, J., et al. (2017). "Prevalence of mcr-1 in Escherichia coli and *Klebsiella pneumoniae* recovered from bloodstream infections in China: a multicentre longitudinal study." The Lancet Infectious Diseases **17**(4): 400-410 | Subgroup, cannot derive the number of patients who died with KPB |
| 104 | Venugopalan, V., et al. (2017). "Double carbapenem therapy (DCT) for bacteremia due to carbapenem-resistant *Klebsiella pneumoniae* (CRKP): from test tube to clinical practice." Infectious Diseases **49**(11-12): 867-870 | N=36 |
| 105 | Xiao, S. Z., et al. (2017). "The Resistance Phenotype and Molecular Epidemiology of *Klebsiella pneumoniae* in Bloodstream Infections in Shanghai, China, 2012-2015." Frontiers in Microbiology **8**: 1-8 | No KPB mortality data |
| 106 | Yu, W. L., et al. (2017). "Impacts of Hypervirulence Determinants on Clinical Features and Outcomes of Bacteremia Caused by Extended-Spectrum β-Lactamase-Producing *Klebsiella pneumoniae.*" Microb Drug Resist **23**(3): 376-383 | N=48 |
| 107 | Zheng, X., et al. (2017). "Clinical and molecular characteristics, risk factors and outcomes of Carbapenem-resistant *Klebsiella pneumoniae* bloodstream infections in the intensive care unit." Antimicrob Resist Infect Control **6**: 102 | N=48 |
| 108 | Antochevis, L. C., et al. (2018). "KPC-producing *Klebsiella pneumoniae* bloodstream isolates from Brazilian hospitals: What (still) remains active?" Journal of global antimicrobial resistance **15**: 173-177 | No KPB mortality data |
| 109 | Baker, T. M., et al. (2018). "Epidemiology of Bloodstream Infections Caused by Escherichia coli and *Klebsiella pneumoniae* That Are Piperacillin-Tazobactam-Nonsusceptible but Ceftriaxone-Susceptible." Open Forum Infect Dis **5**(12): ofy300 | Subgroup, cannot derive the number of patients who died with KPB |
| 110 | Burnham, J. P., et al. (2018). "Differences in mortality between infections due to extended-spectrum-beta-lactamase-producing *Klebsiella pneumoniae* and Escherichia coli." Infect Control Hosp Epidemiol **39**(9): 1138-1139 | Letter |
| 111 | Davido, B., et al. (2018). "Extended-Spectrum Beta-Lactamase (ESBL)-Producing Escherichia coli versus *Klebsiella pneumoniae:* Does type of germ really matter?" Infect Control Hosp Epidemiol **39**(9): 1137-1138 | Letter |
| 112 | Duani, H., et al. (2018). "A shorter period of therapy is associated with higher mortality in bloodstream infections caused by carbapenemase-producing *Klebsiella pneumoniae* in a Brazilian centre." Infectious diseases (London, England) **50**(2): 156-161 | N=31, letter |
| 113 | El Chakhtoura, N. G., et al. (2018). "Comparing predictive performance of INCREMENT scores on mortality among patients with carbapenem-non-susceptible (CNS) *Klebsiella pneumoniae* (Kp) and enterobacter cloacae complex (Ecc) bloodstream infections (BSI) in the veterans health administration (VHA)." Open Forum Infectious Diseases **5**: S715. | Conference abstract |
| 114 | El Kholy, A. and A. El Manakhly (2018). "Molecular epidemiology of carbapenem-resistant *Klebsiella pneumoniae* (CRKP) causing central line associated blood stream infections (CLABSI) in three ICU units in Egypt." Open Forum Infectious Diseases **5**: S682 | Conference abstract |
| 115 | Geng, T. T., et al. (2018). "High-dose tigecycline for the treatment of nosocomial carbapenem-resistant *Klebsiella pneumoniae* bloodstream infections: A retrospective cohort study." Medicine (Baltimore) **97**(8): e9961 | N=40 |
| 116 | Harris, P. N. A., et al. (2018). "Effect of Piperacillin-Tazobactam vs Meropenem on 30-Day Mortality for Patients With E coli or *Klebsiella pneumoniae* Bloodstream Infection and Ceftriaxone Resistance: A Randomized Clinical Trial." JAMA **320**(10): 984-994 | RCT, N=23 |
| 117 | Hayden, M. K. and S. Y. Won (2018). "Carbapenem-sparing therapy for extended-spectrum β-lactamase-producing e coli and *Klebsiella pneumoniae* bloodstream infection: The search continues." JAMA - journal of the american medical association **320**(10): 979-981 | Review |
| 118 | Heng, S. T., et al. (2018). "No association between resistance mutations, empiric antibiotic, and mortality in ceftriaxone-resistant Escherichia coli and *Klebsiella pneumoniae* bacteremia." Sci Rep **8**(1): 12785 | N=43 |
| 119 | Kanwar, A., et al. (2018). "Emergence of Resistance to Colistin During the Treatment of Bloodstream Infection Caused by *Klebsiella pneumoniae* Carbapenemase-Producing *Klebsiella pneumoniae.*" Open Forum Infect Dis **5**(4): ofy054 | Case report |
| 120 | Lee, C. C., et al. (2018). "Propensity-matched analysis of the impact of extended-spectrum beta-lactamase production on adults with community-onset Escherichia coli, klebsiella species, and Proteus mirabilis bacteremia." Journal of Microbiology Immunology and Infection **51**(4): 519-526 | Subgroup, insufficient information to evaluate risk of bias |
| 121 | Lespada, M. I., et al. (2018). "Bacteremia caused by *Klebsiella pneumoniae* carbapenemase (KPC)-producing K. pneumoniae. A retrospective study of 7 years." Rev Esp Quimioter. | N=45 |
| 122 | Man, M. Y., et al. (2018). "Impact of appropriate empirical antibiotics on clinical outcome in *Klebsiella pneumoniae* bacteraemia in the intensive care unit." Intensive Care Medicine Experimental **6** | Conference abstract |
| 123 | Papadimitriou-Olivgeris, M., et al. (2018). "Molecular epidemiology and risk factors for colistin- or tigecycline-resistant carbapenemase-producing *Klebsiella pneumoniae* bloodstream infection in critically ill patients during a 7-year period." Diagn Microbiol Infect Dis **92**(3): 235-240 | Same data described in Papadimitriou-Olivgeris, 2020 Antibiotics (Basel) **9** (11) |
| 124 | Russo, A., et al. (2018). "Comparison of Septic Shock Due to Multidrug-Resistant Acinetobacter baumannii or *Klebsiella pneumoniae* Carbapenemase-Producing K. pneumoniae in Intensive Care Unit Patients." Antimicrob Agents Chemother **62**(6) | Culture of urine or a biological sample from skin and skin structures, lung or abdomen also included |
| 125 | Sullivan, T., et al. (2018). "The Rapid Prediction of Carbapenem Resistance in Patients with *Klebsiella pneumoniae* Bacteremia Using Electronic Medical Record Data." Open Forum Infectious Diseases **5**(5) | No KPB mortality data |
| 126 | Viau, R., et al. (2018). "How do healthcare providers approach empiric β-lactam (BL) treatment of bloodstream infections (BSI) caused by gram-negative rods (gnrs)? analysis of escherichia coli and *Klebsiella pneumoniae* BSI from the veterans health administration (VHA)." Open Forum Infectious Diseases **5**: S311 | Conference abstract |
| 127 | Wang, Y., et al. (2018). "Epidemiology of carbapenem-resistant *Klebsiella pneumoniae* bloodstream infections after renal transplantation from donation after cardiac death in a Chinese hospital: a case series analysis." Antimicrob Resist Infect Control **7**: 66 | Case report |
| 128 | Wilson, B., et al. (2018). "Using the desirability of outcome ranking for management of antimicrobial therapy (DOOR-MAT) to assess antibiotic therapy guided by rapid molecular diagnostics (RMD) in bloodstream infection (BSI) caused by escherichia coli and *Klebsiella pneumoniae.*" Open Forum Infectious Diseases **5**: S60. | Conference abstract |
| 129 | Yang, C. Y., et al. (2018). "Etiology of community-onset monomicrobial bacteremic pneumonia and its clinical presentation and outcome: Klebsiella and Pseudomonas matters." J Infect Chemother **24**(1): 53-58 | *Klebsiella* species |
| 130 | Aslan, A. T., et al. (2019). "Comparison of 30-day crude mortality rates in patients with bloodstream infections (bsis) caused by colistin susceptible-(ColS-CRKp) vs. colistin and carbapenem-resistant *Klebsiella pneumoniae* (ColR-CRKp)." Open Forum Infectious Diseases **6**: S779-S780 | Conference abstract |
| 131 | Bayraktar, B., et al. (2019). "Trend in Antibiotic Resistance of Extended-Spectrum Beta-Lactamase-Producing Escherichia Coli and *Klebsiella pneumoniae* Bloodstream Infections." Medical Bulletin of Sisli Etfal Hospital **53**(1): 70-75 | Subgroup, cannot derive the number of patients who died with KPB |
| 132 | Ben-Chetrit, E., et al. (2019). "Associated factors and clinical outcomes of bloodstream infection due to extended-spectrum β-lactamase-producing Escherichia coli and *Klebsiella pneumoniae* during febrile neutropenia." Int J Antimicrob Agents **53**(4): 423-428 | Subgroup, cannot derive the number of patients who died with KPB |
| 133 | Blot, K., et al. (2019). "Increasing burden of Escherichia coli, *Klebsiella pneumoniae,* and Enterococcus faecium in hospital-acquired bloodstream infections (2000-2014): A national dynamic cohort study." Infect Control Hosp Epidemiol **40**(6): 705-709 | Subgroup, cannot derive the number of patients who died with KPB |
| 134 | Del Prete, R., et al. (2019). "Trends in *Klebsiella pneumoniae* strains isolated from the bloodstream in a teaching hospital in southern Italy." Infez Med **27**(1): 17-25 | No KPB mortality data |
| 135 | Dini, S., et al. (2019). "Geriatric frailty and clinical response to ceftazidime-avibactam therapy in blood-stream infections caused by carbapenemaseproducing *Klebsiella pneumoniae.*" European Geriatric Medicine **10**: S21 | Conference abstract |
| 136 | Fiori, B., et al. (2019). "Direct use of eazyplex (R) SuperBug CRE assay from positive blood cultures in conjunction with inpatient infectious disease consulting for timely appropriate antimicrobial therapy in Escherichia coli and *Klebsiella pneumoniae* bloodstream infections." Infect Drug Resist **12**: 1055-1062 | No KPB mortality data |
| 137 | Harris, P. N. A., et al. (2019). "MERINO Trial Investigators and the Australasian Society for Infectious Disease Clinical Research Network (ASID-CRN). Effect of piperacillin- tazobactam vs meropenem on 30-day mortality for patients with E coli or *Klebsiella pneumoniae* bloodstream infection and ceftriaxone resistance: a randomized clinical trial (vol 320, pg 984, 2018)." Jama-Journal of the American Medical Association **321**(23): 2370-2370 | Letter |
| 138 | Liang, Q., et al. (2019). "Early use of polymyxin B reduces the mortality of carbapenem-resistant *Klebsiella pneumoniae* bloodstream infection." Braz J Infect Dis **23**(1): 60-65 | N=40 |
| 139 | Lin, Y. T., et al. (2019). "Appropriate Treatment for Bloodstream Infections Due to Carbapenem-Resistant *Klebsiella pneumoniae* and Escherichia coli: A Nationwide Multicenter Study in Taiwan." Open Forum Infect Dis **6**(2): ofy336 | Subgroup, insufficient information to evaluate risk of bias |
| 140 | Machuca, I., et al. (2019). "External validation of the INCREMENT-CPE mortality score in a carbapenem-resistant *Klebsiella pneumoniae* bacteraemia cohort: the prognostic significance of colistin resistance." International Journal of Antimicrobial Agents **54**(4): 442-448 | Same data described in Machuca 2017 Antimicrob Agents Chemother 61(8) |
| 141 | MacVane, S. H., et al. (2019). "Carbapenem vs. piperacillin-tazobactam definitive therapy for patients with bloodstream infections due to ceftriaxone not susceptible escherichia coli or klebsiella species." Open Forum Infectious Diseases **6**: S785 | Conference abstract |
| 142 | Magira, E., et al. (2019). "Bloodstream klebsiella pneumonia infection and carbapenem combination treatment regimen in high-dose vasopressor-dependent septic shock." Open Forum Infectious Diseases **6**: S781. | Conference abstract |
| 143 | Namikawa, H., et al. (2019). "Clinical characteristics of bacteremia caused by hypermucoviscous *Klebsiella pneumoniae* at a tertiary hospital." Diagn Microbiol Infect Dis **95**(1): 84-88 | Same data described in Namikawa, 2019 Eur J Clin Microbiol Infect Dis **38**(12): 2291-2297 |
| 144 | Prawang, A., et al. (2019). "Treatment and clinical outcomes among infected patients with colistinresistant *Klebsiella pneumoniae* bacteremia." Open Forum Infectious Diseases **6**: S782-S783 | Conference abstract |
| 145 | Sawatwong, P., et al. (2019). "High Burden of Extended-Spectrum beta-Lactamase-Producing Escherichia coli and *Klebsiella pneumoniae* Bacteremia in Older Adults: A Seven-Year Study in Two Rural Thai Provinces." American Journal of Tropical Medicine and Hygiene **100**(4): 943-951 | No KPB mortality data |
| 146 | Sianipar, O., et al. (2019). "Mortality risk of bloodstream infection caused by either Escherichia coli or *Klebsiella pneumoniae* producing extended-spectrum β-lactamase: a prospective cohort study." BMC Res Notes **12**(1): 719 | Subgroup, cannot derive the number of patients who died with KPB |
| 147 | Umit, Z., et al. (2019). "Evaluation of clinical features, carbapenem resistance and risk factors of klebsiella species: A 4-year retrospective study in Turkey." Open Forum Infectious Diseases **6**: S777-S778 | Conference abstract |
| 148 | Youssef, D., et al. (2019). "*Klebsiella pneumoniae* and K. Oxytoca Bacteremia: Differences in Host, Source, and Antibiotic Susceptibility." Open Forum Infectious Diseases **6**: S112 | Conference abstract |
| 149 | Assimakopoulos, S. F., et al. (2020). "Predictors of mortality for KPC-producing *Klebsiella pneumoniae* bloodstream infections in adult neutropenic patients with haematological malignancies." Infectious diseases (London, England) **52**(6): 446-449 | Letter, N=39 |
| 150 | Baek, E. H., et al. (2020). "Successful control of an extended-spectrum beta-lactamase-producing *Klebsiella pneumoniae* ST307 outbreak in a neonatal intensive care unit." BMC Infect Dis **20**(1): 166 | N=6 |
| 151 | Chen, F. C., et al. (2020). "Does inappropriate initial antibiotic therapy affect in-hospital mortality of patients in the emergency department with Escherichia coli and *Klebsiella pneumoniae* bloodstream infections?" Int J Immunopathol Pharmacol **34**: 2058738420942375 | Subgroup, insufficient information to evaluate risk of bias |
| 152 | Essel, V., et al. (2020). "A multisectoral investigation of a neonatal unit outbreak of *Klebsiella pneumoniae* bacteraemia at a regional hospital in Gauteng Province, South Africa." S Afr Med J **110**(8): 783-790 | No 51 babies' mortality data, only has 12 babies' data |
| 153 | Ghaith, D. M., et al. (2020). "Genetic diversity of carbapenem-resistant *Klebsiella pneumoniae* causing neonatal sepsis in intensive care unit, Cairo, Egypt." Eur J Clin Microbiol Infect Dis **39**(3): 583-591 | N=23 |
| 154 | Ham, S. Y., et al. (2020). "Differences in clinical characteristics of third generation cephalosporin resistance and treatment outcomes in escherichia coli and *Klebsiella pneumoniae* bacteremia in patients with liver cirrhosis." Open Forum Infectious Diseases **7**(SUPPL 1): S798 | Conference abstract |
| 155 | Kochan, T. J., et al. (2020). Genomic surveillance for multidrug-resistant or hypervirulent *Klebsiella pneumoniae* among bloodstream isolates at a United States academic medical center | No KPB mortality data |
| 156 | Piccirilli, A., et al. (2020). "Molecular characterization of carbapenem-resistant *Klebsiella pneumoniae* ST14 and ST512 causing bloodstream infections in ICU and surgery wards of a tertiary university hospital of Verona (northern Italy): co-production of KPC-3, OXA-48, and CTX-M-15 beta-lactamases." Diagn Microbiol Infect Dis **96**(3) | No KPB mortality data |
| 157 | Richelsen, R., et al. (2020). "Outcome of community-onset ESBL-producing Escherichia coli and *Klebsiella pneumoniae* bacteraemia and urinary tract infection: a population-based cohort study in Denmark." Journal of Antimicrobial Chemotherapy **75**(12): 3656-3664 | Subgroup, insufficient information to evaluate risk of bias |
| 158 | Weston, G., et al. (2020). "Derivation of a Model to Guide Empiric Therapy for Carbapenem-Resistant *Klebsiella pneumoniae* Bloodstream Infection in an Endemic Area." Open Forum Infectious Diseases **7**(7) | No KPB mortality data |
| 159 | You, T., et al. (2020). "Differences in clinical characteristics of early- and late-onset neonatal sepsis caused by *Klebsiella pneumoniae.*" Int J Immunopathol Pharmacol **34**: 2058738420950586 | Not all samples are blood, partial cerebrospinal fluid culture |
| 160 | Zhang, P., et al. (2020). "Clinical Characteristics and Risk Factors for Bloodstream Infection Due to Carbapenem-Resistant *Klebsiella pneumoniae* in Patients with Hematologic Malignancies." Infect Drug Resist **13**: 3233-3242 | N=22 |
| 161 | Zhen, X. M., et al. (2020). "Clinical and Economic Impact of Third-Generation Cephalosporin-Resistant Infection or Colonization Caused by Escherichia coli and *Klebsiella pneumoniae:* A Multicenter Study in China." Int J Environ Res Public Health **17**(24) | Not all were bloodstream infections |
| 162 | Bor, M. and O. Ilhan (2021). "Carbapenem-Resistant *Klebsiella pneumoniae* Outbreak in a Neonatal Intensive Care Unit: Risk Factors for Mortality." J Trop Pediatr **67**(3) | Blood, urine, or cerebrospinal fluid cultures |
| 163 | Chen, J., et al. (2021). "Shift in the Dominant Sequence Type of Carbapenem-Resistant *Klebsiella pneumoniae* Bloodstream Infection from ST11 to ST15 at a Medical Center in Northeast China, 2015-2020." Infect Drug Resist **14**: 1855-1863 | N=42 |
| 164 | Coyne, A. J. K., et al. (2021). "Influence of Antimicrobial Stewardship and Molecular Rapid Diagnostic Tests on Antimicrobial Prescribing for Extended-Spectrum Beta-Lactamase- and Carbapenemase-Producing Escherichia coli and *Klebsiella pneumoniae* in Bloodstream Infection." Microbiology spectrum **9**(2) | Subgroup, cannot derive the number of patients who died with KPB |
| 165 | Frescas, B. E., et al. (2021). "Outcomes Associated with Empiric Cefepime or Meropenem for Bloodstream Infections Caused by Ceftriaxone-Resistant, Cefepime-Susceptible Escherichia coli and *Klebsiella pneumoniae.*" Open Forum Infectious Diseases **8**(SUPPL 1): S703 | Conference abstract |
| 166 | Gaibani, P., et al. (2021). "Epidemiology of Meropenem/Vaborbactam Resistance in KPC-Producing *Klebsiella pneumoniae* Causing Bloodstream Infections in Northern Italy, 2018." Antibiotics (Basel) **10**(5) | N=8 |
| 167 | Kim, S. H. and Y. M. Wi (2021). "Clinical characteristics of hypermucoviscous *Klebsiella pneumoniae* bacteremia with non-hepatobiliary infection." International Journal of Antimicrobial Agents **58** | Only abstract |
| 168 | Li, S., et al. (2021). "Clinical features and development of Sepsis in *Klebsiella pneumoniae* infected liver abscess patients: a retrospective analysis of 135 cases." BMC Infect Dis **21**(1): 597 | N=37 |
| 169 | Liang, T., et al. (2021). "Epidemiology, Risk Factors, and Clinical Outcomes of Bloodstream Infection due to Extended-Spectrum Beta-Lactamase-Producing Escherichia coli and *Klebsiella pneumoniae* in Hematologic Malignancy: A Retrospective Study from Central South China." Microb Drug Resist **27**(6): 800-808 | Subgroup, cannot derive the number of patients who died with KPB |
| 170 | Luo, H., et al. (2021). "Comparison of therapy with β-lactam/β-lactamase inhibitor combinations or carbapenems for bacteraemia of nonurinary source caused by ESBL-producing Escherichia coli or *Klebsiella pneumoniae.*" Annals of clinical microbiology and antimicrobials **20**(1): 63 | Subgroup, cannot derive the number of patients who died with KPB |
| 171 | Mestrovic, T. (2021). "Antimicrobial resistance in *Klebsiella pneumoniae* as an independent risk factor for bacteraemia-related mortality." Hong Kong Medical Journal **27**(5): 385-385 | Letter |
| 172 | Micozzi, A., et al. (2021). "Reduced mortality from KPC-K.pneumoniae bloodstream infection in high-risk patients with hematological malignancies colonized by KPC-K.pneumoniae." Bmc Infectious Diseases **21**(1) | N=34 |
| 173 | Perez-Palacios, P., et al. (2021). "Successful outcome after treatment with a combination of meropenem and fosfomycin for VIM-1 and CTX-M-15 producing *Klebsiella pneumoniae* bloodstream infection." Journal of Infection **83**(4): E12-E13 | Case report |
| 174 | Richelsen, R., et al. (2021). "Risk factors of community-onset extended-spectrum beta-lactamase Escherichia coli and *Klebsiella pneumoniae* bacteraemia: an 11-year population-based case-control-control study in Denmark." Clinical Microbiology and Infection **27**(6): 871-877 | Subgroup, cannot derive the number of patients who died with KPB |
| 175 | Seo, H., et al. (2021). "Risk Factors for Mortality in Patients with *Klebsiella pneumoniae* Carbapenemase-producing K. pneumoniae and Escherichia coli bacteremia." Infect Chemother **53**(3): 528-538 | Subgroup, insufficient information to evaluate risk of bias |
| 176 | Torres, I., et al. (2021). "Performance of a MALDI-TOF mass spectrometry-based method for rapid detection of third-generation oxymino-cephalosporin-resistant Escherichia coli and Klebsiella spp. from blood cultures." European Journal of Clinical Microbiology and Infectious Diseases **40**(9): 1925-1932 | Subgroup, cannot derive the number of patients who died with KPB |
| 177 | Wu, H. (2021). "The Value of Neutrophil to Lymphocyte Count Ratio for Predicting the Clinical Outcomes of Patients with Carbapenem-resistant Klebsiella pneumonia Blood Stream Infection." Open Forum Infectious Diseases **8**(SUPPL 1): S219 | Conference abstract |
| 178 | Xiao, S. Z., et al. (2021). "Drug Susceptibility and Molecular Epidemiology of *Klebsiella pneumoniae* Bloodstream Infection in ICU Patients in Shanghai, China." Frontiers in Medicine **8** | No KPB mortality data |
| 179 | Zhu, X. Y., et al. (2021). "Clinical characteristics and risk factors associated with secondary bloodstream infection in patients with intensive care unit-acquired pneumonia due to carbapenem-resistant *Klebsiella pneumoniae.*" Chinese Medical Journal **134**(14): 1735-1737 | N=18 |
| 180 | Belati, A., et al. (2022). "Meropenem/Vaborbactam Plus Aztreonam as a Possible Treatment Strategy for Bloodstream Infections Caused by Ceftazidime/Avibactam-Resistant *Klebsiella pneumoniae:* A Retrospective Case Series and Literature Review." Antibiotics **11**(3) | N=3 |
| 181 | Isler, B., et al. (2022). "Characteristics and outcomes of carbapenemase harbouring carbapenem-resistant Klebsiella spp. bloodstream infections: a multicentre prospective cohort study in an OXA-48 endemic setting." European Journal of Clinical Microbiology & Infectious Diseases | Seven were speciated as *K. variicola,* one *K. quasipneumoniae* and one *K. oxytoca.* |
| 182 | Zhang, Z., et al. (2022). "Antimicrobial Resistance Among Pathogens Causing Bloodstream Infections: A Multicenter Surveillance Report Over 20 Years (1998-2017)." Infect Drug Resist **15**: 249-260 | Subgroup, no KPB mortality data |
| 183 | Kochan, T. J., et al. (2022). "Genomic surveillance for multidrug-resistant or hypervirulent *Klebsiella pneumoniae* among United States bloodstream isolates." BMC Infect Dis **22**(1): 603 | No KPB mortality data |
| 184 | Lanzafame, M., et al. (2022). "*Klebsiella pneumoniae* resistant to carbapenems: Bloodstream infection prevalence before and during the SARS-CoV-2 pandemic in a tertiary level, North-Eastern Italian Hospital." Recenti Prog Med **113**(3): 213-215 | The full text was not available |
| 185 | McAlister, M. J., et al. (2022). "Oral β-lactams vs fluoroquinolones and trimethoprim/sulfamethoxazole for step-down therapy for Escherichia coli, Proteus mirabilis, and *Klebsiella pneumoniae* bacteremia." Am J Health Syst Pharm | Subgroup, no KPB mortality data |
| 186 | Sadaniantz, K., et al. (2022). "A STICKY SITUATION: HYPERVIRULENT KLEBSIELLA BACTEREMIA IN A DIABETIC PATIENT." Journal of General Internal Medicine **37**: S421-S422 | Conference abstract |

N, number of included patients; KPB, *Klebsiella pneumoniae* bacteraemia

**Table S3 Characteristics of individual studies**

| First author,  published year | Number of patients | Study period | Study design | Country | Sex, male (n, %) | Age(years) [mean (IQR)] | ICU (n, %) | Resistance type | HA (n, %) | Study purpose |
| --- | --- | --- | --- | --- | --- | --- | --- | --- | --- | --- |
| Pena, 2001 | 92 | 1993-1995 | PC, single center | Spain | 65 (70.7) | 60.6+-13.6 | 64 (69.6) | ESBL-KP vs non-ESBL-KP | 92 (100.0) | To describe the clinical outcome of ESBL-KPB |
| Kim, 2002 | 158 | 1999- 2001 | RC, single center | Korea | 106 (67.1) | >65, 34 (21.5) | 10 (6.3) | ESBL-KP vs non-ESBL-KP | 88 (55.7) | To identify the clinical implications of ESBL-KPB |
| Tsay, 2002 | 158 | 1997- 1999 | PC, single center | China Taiwan | 1.4:1 | 60+-19 | NA | NA | 64 (40.5) | Compare the clinical and bacteriological characteristics of KPB |
| Paterson, 2004 | 85 | 1996 -  1997 | PC, multicenter | multiple countries | 42(49.4) | >6, NA | 28 (39.4) | ESBL-KP | NA | Evaluate impact of antibiotic therapy on the treatment of ESBL-KPB |
| Anderson, 2006 | 60 | 1995- 2003 | RC, single center | North Carolina | 38(63.3) | Died: 47.9 +-28.1 | 17 (28.3) | CAZ-R | 43 (71.7) | To identify predictors of in-hospital mortality among patients with CAZ-R-KPB |
| Kang, 2006 | 377 | 1998-2002 | RC, single center | Korea | 249 (66.0) | CA: 57±13 | 14 (3.7) | NA | 186 (49.3) | compare clinical features, outcomes in KPB acquired as CA vs HA |
| Marra, 2006 | 108 | 1996-2001 | RC, single center | Brazilian | 53 (49.1) | 27.3 (range: 0-78) | 49 (45.4) | ESBL-KP vs non-ESBL-KP | 108 (100.0) | Evaluate whether ESBL-KP is associated with a high mortality |
| Robenshtok, 2006 | 90 | 2000- 2003 | RC, single center | Israel | 50 (55.6) | 70+-15 | 10 (11.1) | NA | 90 (100.0) | Assess whether AB is associated with increased mortality compared with nosocomial KPB |
| Tumbarello, 2006 | 147 | 1999- 2003 | RCC, single center | Italy | 104 (70.7) | 64 +- 16 | NA | ESBL-KP vs non-ESBL-KP | 128 (87.1) | To identify the factors associated with ESBL-KPB |
| Mosqueda-Gómez, 2008 | 121 | 1993-2002 | RCC, single center | Mexico | 57 (47.1) | median:48.1 (range 15-85) | 32 (26.4) | ESBL-KP vs SKP | 64 (52.9) | To compare the prevalence, risk factors, outcome, and molecular epidemiology in patients with ESBL-KP and SKP |
| Daikos, 2009 | 162 | 2004-2006 | PC, multicenter | Greece | 103 (63.6) | 60.3(range, 17-97) | NA | VIM-1 vs non-VIM-1 | 162 (100.0) | Evaluate the importance of VIM production on outcome of patients with KPB |
| Liao, 2009 | 231 | 2007 | PC, single center | China Taiwan | 150 (64.9) | 61.7 ± 15.8 | 32 (13.9) | NA | NA | To investigate the association between time to positivity in patients with KPB |
| Meatherall, 2009 | 584 | 2000-2007 | RC, multicenter | Canada | 370 (63.4) | Median: 68.9 (53.0-79.3) | NA | NA | 418 (71.6) | To describe the incidence of risk factors for, and outcomes associated with KPB |
| Szilágyi, 2009 | 200 | 2005-2008 | RC, multicenter | Hungary | 134 (67.0) | ESBL: median: 63 (48-74) | 141 (70.5) | ESBL-KP vs non-ESBL-KP | NA | Investigate risk factors for and outcomes  of ESBL and non-ESBL-KPB |
| Mouloudi, 2010 | 59 | 2007-2008 | nested case-control，single center | Greece | 45 (76.3) | median: 53 (range, 15-81) | 59 (100.0) | CRKP vs CSKP | NA | To determine risk factors for bloodstream infections caused by CRKP |
| Pépin, 2010 | 411 | 1997-2007 | RC, single center | Canada | 206 (50.1) | >=65, 278/411 | 116 (28.2) | NA | 153 (37.2) | To modulate the incidence of KPB |
| Tsai, 2010 | 193 | 2005-2006 | RC, single center | China Taiwan | 109 (56.5) | 63.5 (range, 20-101) | NA | NA | 46 (23.8) | Analyze the characteristics, risk factors, and outcomes of diabetic patients with CA vs HA KPB |
| Francisco Tuon, 2011 | 104 | 2006-2009 | RC, single center | Brazil | 59 (56.7) | Median, 43.0 (range, 12-86) | 72 (69.2) | ESBL-KP vs non-ESBL-KP | NA | To evaluated risk factors for mortality in patients with BSI caused by ESBL-KP |
| Lee, 2011 | 252 | 2002-2009 | case-comparator, single center | Korea | 153 (61.1) | >=65, 114/252 | NA | ESBL-KP vs non-ESBL-KP | NA | Evaluate risk factors and clinical outcomes of community-onset bacteremia caused by ESBL-KP |
| Lin, 2011 | 189 | 2007-2009 | RC, single center | China Taiwan | 132 (69.8) | >65, 103/189 | NA | NA | 189 (100.0) | Investigate the clinical characteristics of neutropenic and non-neutropenic adult cancer patients with nosocomial KPB |
| Neuner, 2011 | 51 | 2007-2009 | RC, single center | USA | 32 (62.7) | 60.4+-1.8 | 26 (51.0) | CRKP | NA | To describe the treatment and outcomes of CRKP BSI |
| Webster, 2011 | 446 | 1999-2010 | RCC, multicenter | UK | 265 (59.4) | 65 (16-102) | 59 (13.2) | NA | 191 (42.8) | To compared trends in rates of resistance in KP and E. coli |
| Zarkotou, 2011 | 53 | 2008-2010 | PCC, single center | Greece | 33 (62.3) | 63.8 +-19.9 | 38 (71.7) | KPC-KP | NA | Identify outcomes, risk factors for mortality and impact of appropriate treatment of BSIs caused by KPC-KP |
| Ben-David, 2012 | 192 | 2006 | RC, single center | Israel | 111 (57.8) | Median, 73+-27 | 63 (32.8) | SKP vs ESBL-KP vs CRKP | 171 (89.1) | To evaluate the impact of CRKP BSIs on mortality. |
| Jung, 2012 | 553 | 2003-2008 | RC, two hospitals | Korea | 348 (62.9) | 61 (range, 18-103) | NA | NA | NA | To evaluate the impact of HCA infection on mortality in patients with community-onset KP BSIs |
| Lee, J. A., 2012 | 240 | 2006-2009 | RC, single center | Korea | 149 (62.1) | >=65, 105/240 | NA | NA | NA | To delineate the clinical differences between CA and HCA KPB |
| Lee, N. Y., 2012 | 103 | 2008-2009 | RC, single center | China Taiwan | 61 (59.2) | 64.8+-18.5 | NA | ETP-S vs Revised ETP-NS | NA | To characterize KP isolates that were revised as carbapenem-nonsusceptible KP with the lowered breakpoints |
| Liu, 2012 | 75 | 2007-2009 | RCC, single center | China Taiwan | 48 (64.0) | 64.9+-16.0 | 35 (46.7) | ETP-S vs ETP-NS | NA | Investigate potential risk factors for the development of ETP-NS |
| Tumbarello, 2012 | 125 | 2010-2011 | RC, multicenter | Italy | 73 (58.4) | Nonsurvivors, 61.5+-14.3 | 53 (42.4) | KPC-KP | 125 (85.0) | To pinpoint risk factors for mortality in patients with BSIs caused by KPC-KP |
| Wu, 2012 | 372 | 2007-2010 | RC, single center | China Taiwan | 254 (68.3) | CA group, 69 +-15.8 | 75 (20.2) | NA | NA | To investigate the clinical features and outcomes of CA and HCA KPB |
| Hussein, 2013 | 317 | 2006-2008 | RCC, single center | Israel | 206 (65.0) | 62.6+-17.7 | 57 (18.0) | CRKP vs CSKP | 317 (100.0) | To identify risk factors for CRKP |
| Kang, 2013 | 248 | 2007-2011 | RCC, two hospitals | Korea | 180 (72.6) | 63.8+-14.6 | 48 (19.4) | NA | 89 (35.9) | To determine the risk factors for persistent bacteremia and to reevaluate the need for routine follow-up blood cultures in KPB |
| Tseng, 2013 | 309 | 2007-2009 | RC, single center | China Taiwan | 208 (67.5) | 70.6 +-15.1 | 58 (18.8) | NA |  | To investigate the clinical manifestations, predictive factors for  mortality of patients with community-onset KPB |
| Chetcuti Zammit,  2014 | 186 | 2007-2012 | RC, single center | Malta | 97 (52.2) | 62+-21.3 | 18 (9.7) | NA | NA | To assess underlying co-morbidities in patients diagnosed with KPB |
| Daikos, 2014 | 205 | 2009-2010 | RC, two hospitals | Greece | 118 (57.6) | median, 66 (range, 17-90) | 116 (56.6) | CRKP | 190 (92.7) | To evaluate the clinical outcome of patients with CRKP BSI |
| Gallagher, 2014 | 308 | 2005-2010 | RCCC, single center | USA | 161 (52.3) | >18 years | 69 (22.4) | 3GCRKP vs CRKP | NA | To determine the risk factors for BSIs caused by 3GCRKP and CRKP |
| Giannella, 2014 | 143 | 2012-2013 | PCC, multicenter | Italy | 84 (58.7) | Median, 65 (IQR, 52-75) | 61 (42.7) | CRKP | 143 (100.0) | To identify risk factors for CRKP BSI among carriers |
| Girometti, 2014 | 217 | 2010-2012 | RC, single center | Italy | 139 (64.1) | Non-Survivors, 68.5+-14.0 | 41 (18.9) | NA | NA | To analyze the impact of resistance on the appropriateness of empirical therapy and treatment outcomes of KPB |
| Gurntke, 2014 | 352 | 2008-2011 | RC, single center | Germany | 231 (65.6) | >65, 168/352 | 242 (68.8) | ESBL-KP vs non-ESBL KP | 215 (61.1) | To assess current distribution of ESBL genotypes and hospital mortality in cases of BSI with ESBL-KP |
| Li, 2014 | 118 | 2009-2011 | RC, single center | China | 63 | Median, 61.0 (range, 29-91) | NA | ESBL-KP and non-ESBL-KP | 118 (100.0) | To identify the risk factors for BSIs with ESBL-KP |
| Liu, 2014 | 70 | 2008-2012 | RC, single center | China | 50 | >60, 38/70 | NA | NA | 32 (45.7) | To identify clinical and molecular Characteristics of emerging hypervirulent KPB |
| Papadimitriou-Olivgeris, 2014 | 53 | 26-month period | RC, single center | Greece | 29 | 58.2+-16.0 | 53 (100.0) | KPC-KP | NA | To identify the risk factors for KPC-KP bloodstream infections in ICU  patients |
| Yang, 2014 | 64 | 2001-2009 | RC, single center | China Taiwan | 47 | 65.0+-10.1 | 32 (50.0) | ESBL-KP | 64 (100.0) | To describe clinical effectiveness between ertapenem and flomoxef for hemodialysis access-related ESBL-KP bacteremia |
| Alicino, 2015 | 329 | 2007-2014 | RC, single center | Italy | 231/349 | 68 (57-76) | NA | CRKP | NA | To describe CRKP BSI epidemiology |
| Chang, 2015 | 171 | 2008-2014 | RC, single center | China Taiwan | 113 | 60.4+-16.9 | 17 (9.9) | NA | NA | To study the impact of occult KPB |
| Cheng, 2015 | 94 | 2010-2014 | RC, single center | China | 61 | 11 months (6, 24) | 94 (100.0) | MDR-KP vs non-MDR-KP |  | To explore the risk factors for sepsis caused by MDR-KP |
| Giacobbe, 2015 | 426 | 2010-2014 | RCC, multicenter | Italy | 269 | 67 (55-77) | NA | ColR KPC-KP vs ColS KPC-KP | 386 (90.6) | To assess risk factors for to describe mortality and clinical characteristic of ColR KPC-KP BSI |
| Gomez-Simmonds, 2015 | 223 | 2012-2013 | RC, single center | USA | 127 | >65, 98/223 | 124 (55.6) | NA | NA | To compare the genetic diversity and clinical features of CRKP, cephalosporin resistant KP |
| Gonzalez-Padilla, 2015 | 50 | 2012-2013 | RC, single center | Spain | 32 | 60.5 (19-86) | 22 (44.0) | ColR KPC-KP | NA | Analyze the impact of gentamicin therapy on mortality from sepsis caused by ColR KPC-KP |
| Huang, 2015 | 341 | 2005-2008 | RC, single center | China Taiwan | 118 | 63.0+-13.2 | NA | NA | NA | Investigate the clinical features affecting mortality in patients with type2 diabetes complicated by CA KPB |
| Malaki, 2015 | 51 | NA | Cross-sectional, single center | Iran | 32 | neonates | 51 (100.0) | NA | NA | To review the incidence of risks factors for, and complications of KPB |
| Pau, 2015 | 208 | 2009-2011 | RC, single center | China Hong Kong | 108 | 68.6+-16.8 | NA | NA | 114 (54.8) | To describe patient characteristics, outcomes between patients with HCA and CA KPB |
| Togawa, 2015 | 83 | 2009-2013 | RC, single center | Japan | 43 | >65, 51/83 | NA | NA | 51 (61.4) | Precise virulence factor of KPB and its relationship with the presence of invasive syndrome |
| Brady, 2016 | 564 | 2008-2013 | RC, 58 acute hospitals | Ireland | NA | NA | NA | NA | 255 (45.2) | To describe the epidemiology and antimicrobial resistance trends of KPB |
| Buys, 2016 | 410 | 2006-2011 | retrospective cross-sectional, single center | South Africa | 212 (51.7) | 5.0 months (IQR, 2-16) | 103 (25.1) | NA | 353 (86.1) | To describe the clinical presentation of KPB, risk factors associated with ESBL-KP BSI |
| Durdu, 2016 | 190 | 2012-2016 | RC, single center | Turkey | 114 (60.0) | 62.7+-16.4 | 157 (82.6) | NA | 190 (100.0) | To detect risk factors effecting mortality rate of patients with nosocomial KPB |
| Fraenkel-Wandel, 2016 | 204 | 2016- | RCC, single center | Israel | 113 (55.4) | NA | 36 (17.6) | KPC-KP vs ESBL-KP | NA | To determine the mortality rate of patients with KPC-KP bacteremia compared with ESBL-KP bacteremia |
| Gao, 2016 | 92 | 2010-2016 | RCC, single center | China | 58 (63.0) | 62.2 +-14.1 | NA | NA | 25 (27.2) | To analyze the clinical features and antibiotic susceptibility of diabetic and non-diabetic patients with KPB |
| Gomez-Simmonds, 2016 | 141 | 2006-2013 | RC, two medical centers | USA | 86 (61.0) | 62 [IQR], 50 to 74 | 87 (61.7) | CRKP | NA | To assess the use of combination therapy for the treatment of CRKP BSI |
| Juan, 2016 | 86 | 2011-2015 | RCC, single center | China Taiwan | 60 (69.8) | 70.8+-13.9 | 11 (12.8) | TCG-S vs TCG-R | 54 (62.8) | To identify the risk factors and outcomes for adult patients with TCG-R KPB |
| Li, 2016 | 116 | 2013-2014 | RC, single center | China | 68 (58.6) | ESBL:50.1+-17.9 | NA | ESBL-KP vs none-ESBL-KP | 64 (55.2) | To evaluate the impact of ESBL on clinical outcome and medical cost in patients with KPB |
| Luk, 2016 | 109 | 2004-2008 | PC, multicenter | China Hong Kong | 65 (59.6) | 72 (62–82) | NA | PAmpC-KP group vs ESBL-KP group | 77 (70.6) | To compare the prevalence, risk factors and clinical features of bacteremia caused by pAmpC-KP |
| Martelius, 2016 | 661 | 1999-2010 | PC, multicenter | Finland | 383 (57.9) | >65,285 (43.3) | 72 (10.9) | NA | 661 (100.0) | To evaluate trends, patient characteristics and mortality of nosocomial KPB |
| Tian, 2016 | 114 | 2011-2015 | RC, multicenter | China | 74 (64.9) | 56.37+-16.36 | 26 (22.8) | CRKP vs CSKP | 98 (86.0) | To describe the epidemiological, microbiological, and clinical characteristics of KPB |
| Tofas, 2016 | 50 | 2010-2014 | RC, single center | Greece | 31 (62.0) | Median, 58 (range, 18-76) | NA | CRKP | 50 (100.0) | To describe the clinical features, treatment and outcomes of patients with HMs complicated with CPKP BSIs |
| Trecarichi, 2016 | 278 | 2010-2014 | PC, multicenter | Italy | 151 (54.3) | >55,152 | NA | CRKP vs CSKP | 249 (89.6) | To identify risk factors for mortality in patients suffering from HMs with KPB |
| Basaranoglu, 2017 | 111 | 2011-2015 | RC, single center | Turkey | 58 (52.3) | 8 months (IQR: 2–45.6) | NA | ESBL-KP vs none-ESBL-KP | 111 (100.0) | To assess risk factors for health care associated BSIs caused by ESBL-KP in children |
| Giannella, 2017 | 193 | 2010-2016 | PC, single center | Italy | 119 (61.7) | 65 (55-74) | 59 (30.6) | CRKP | NA | To assess risk factors for recurrent CR-KP BSI |
| Kaur, 2017 | 75 | 2011-2015 | RC, single center | India | 52 (69.3) | NA | 60 (80.0) | ColR KP and CRKP | NA | To evaluate clinical outcomes of patients with ColR CRKP BSI |
| Kuo, 2017 | 274 | 2008-2013 | RC, single center | China Taiwan | 95 (34.7) | 66.9+-14.3 | NA | NA | NA | To compare the clinical characteristics, frequencies of abscess occurrences, and clinical outcomes of the adults with community-onset KPB |
| Li, 2017 | 104 | 2011-2015 | RC, single center | China | 65 (62.5) | median, 56.5 (range 15-96) | 22 (21.2) | NA | 48 (46.2) | To determine the risk factors and predictors of mortality caused by KPB |
| Liao, 2017 | 104 | 2012-2014 | RC, single center | China | 79 (76.0) | 67.2+-15.7 | 87 (83.7) | CRKP | 84 (80.8) | To analyze the effect of combinational therapy of fosfomycin and carbapenemase on mortality from sepsis due to CRKP |
| Machuca, 2017 | 104 | 2012-2016 | PC, single center | Spain | 57 (54.8) | Non-survivors, 63.5 (47-72.25) | NA | ColR and high-level meropenem-resistant KPC-KP | 97 (93.3) | To examine the variables associated with mortality due to ColR KPC-KP bacteremia with high-level carbapenem resistance |
| Papadimitriou-Olivgeris, 2017 | 139 | 2012-2015 | RCC, single center | Greece | 106 (76.3) | 56.7+-18.0 | 139 (100.0) | CRKP | NA | To study the risk factors and predictors of mortality of CPKP bacteraemia among critically ill patients |
| Shields, 2017 | 109 | 2009-2017 | RC, single center | USA | 62 (56.9) | Median, 61 (range, 25-91) | 55 (50.5) | CRKP | NA | To compare outcomes of patients treated with ceftazidime-avibactam versus comparators for CRKP BSI |
| Veeraraghavan, 2017 | 113 | 2015-2016 | RC, single center | India | NA | NA | NA | NA | NA | To study the molecular characterization of the enzymatic mechanisms of resistance to β-lactam antibiotics in KPB |
| Zhang, 2017 | 52 | 2011-2014 | RC, single center | China | 35 (67.3) | NDM-1 group: 4.7 (1.4-9.2) | 5 (9.6) | NDM-1 KP vs non-NDM-1 KP | 37 (71.2) | To investigate the clinical characteristics and antibiotic resistance of the BSIs due to NDM-1 producing KP in children |
| Corcione, 2018 | 126 | 2013-2015 | RC, single center | Italy | 80 (63.5) | 62 +-16 | 68 (54.0) | CRKP | NA | To describe the epidemiology and compare risk factors for and mortality in patients with *C. difficile* infection, candidemia and BSIs caused by CPKP or ESBL-E. coli |
| Cristina, 2018 | 213 | 2013-2014 | RC, multicenter | Italy | 139 (65.3) | 72 (61+-78) | 100 (46.9) | CRKP | NA | To assesse the differences in epidemiology, and mortality of CRKP BSI |
| Cubero, 2018 | 348 | 2007-2009 | PC, single center | Spain | 210 (60.3) | 63.7+-15.8 | NA | NA | 223 (64.1) | To analyze the clinical and molecular epidemiology of KPB in adult patients |
| Giannella, 2018 | 595 | 2010-2015 | RC, multicenter | Italy | 366 (61.5) | NA | 225 (37.8) | CRKP | 531 (89.2) | To evaluate the impact of high-dose carbapenem-based combination therapy on clinical outcome in patients with CRKP BSI |
| Hyun, 2018 | 344 | 2004-2005, 2012-2013 | RC, single center | Korea | 202 (58.7) | 68 +-14 | 119 (34.6) | NA | 92 (26.7) | To find out the trends of KPB |
| Li, 2018 | 143 | 2015-2016 | RC, single center | China | 97 (67.8) | 54.1+-17.1 | 59 (41.3) | NA |  | To analyze the risk factors, molecular characteristics and patient mortality of hvKP induced BSI. |
| Scheuerman, 2018 | 222 | 2004-2013 | RC, multicenter | 12 countries | 134 (60.4) | 70 (59-79) | 47 (21.2) | ESBL-KP | 139(62.6) | To compare the epidemiology, clinical characteristics, and mortality of patients with BSIs caused by ESBL-E. coli versus ESBL-KP |
| Shankar, 2018 | 86 | 2014-2015 | RC, single center | India | 56 (65.1) | Median: 37.5 (range, 0.002-80) | NA | CRKP | 79 (91.9) | To characterize carbapenem-resistant hypermucoviscous KP strains isolated  from bacteremia |
| Xiao, 2018 | 428 | 2013-2015 | RC, single center | China | 299 (69.9) | CnKP group, 59.1+-15.4 | 139 (56.0) | CRKP vs CSKP | 387 (90.4) | To identify the risk factors for patients with CRKP bacteremia |
| Xu, 2018 | 285 | 2013-2015 | RC, single center | China | 199 (69.8) | 56.8 +-15.9 | 69 (24.2) | NA | 206 (72.3) | To investigate the prevalence of KPC and virulence factors in KP isolated from patients with BSI |
| Yang, 2018 | 60 | 2011-2016 | RC, single center | China | 41 (68.3) | 66.6+-17.9 | NA | CRKP | NA | To explore the clinical characteristics, risk factors of mortality and antimicrobial therapy of CRKP BSI |
| zhang, 2018 | 138 | 2011-2014 | RCC, single center | China | 80 (58.0) | 24.8 months (1.1-101.6) | 20 (14.5) | CRKP vs CSKP | 114 (82.6) | To evaluate risk factors associated with CRKP BSIs and mortality of KPB among pediatric patients |
| Zheng, 2018 | 289 | 2014-2016 | RC, single center | China | 194 (67.1) | CRKP group, 67.0+-16.7 | 75 (26.0) | CRKP vs CSKP | 167 (57.8) | To identify risk factors, outcomes and genotypes that were correlated with CRKP BSIs |
| Balkhair, 2019 | 375 | 2007-2016 | RC, single center | Oman | 218 (58.1) | Mean, 52.1 | NA | CRKP vs CSKP | NA | To examine the burden of carbapenem and colistin resistance in KPB |
| Brescini, 2019 | 112 | 2011-2015 | RC, single center | Italy | 80 (71.4) | 68 (55-76) | 45 (40.2) | KPC-KP | 104 (92.9) | To evaluate the clinical and epidemiological characteristics of KPC-KP |
| Imai, 2019 | 78 | 2014-2017 | RC, two centres | Japan | 53 (67.9) | median, 74 | NA | NA | 41 (52.6) | To clarify differences in the clinical impact and bacterial characteristics of BSIs associated with KP, *K. variicola,* and *K. quasi pneumoniae* |
| Jahidul Hasan, 2019 | 101 | 2017-2018 | PCS, single center | Bangladesh | 55 (54.5) | Colistin group, median, 56 | 101 (100.0) | NA | NA | To observe the therapeutic effectiveness as well as clinical outcomes of the polymyxin B in comparison to colistin in the treatment of CRKP BSI |
| Juan, 2019 | 337 | 2015 | RC, single center | China Taiwan | 202 (59.9) | 69.9+-15.3 | NA | NA | 165 (49.0) | To compare the clinical characteristics, distribution of capsular types, and antimicrobial resistance of KPB among CA, HCA, and HA. |
| Kim,2019 | 579 | 2016-2017 | PC, multicenter | Korea | 338 (58.4) | 71.0 (60.0-79.3) | 102 (17.6) | NA | NA | To investigate the risk factors affecting clinical prognosis of KP BSI patients |
| Lee, 2019 | 68 | 2012-2013 | RC, multicenter | Korea | 36 (52.9) | Death group, 58.3+-22.3 | 26 (38.2) | NA | 43 (63.2) | To investigate the variability in the rate of persister formation against meropenem in KPB patients |
| Liu, J., 2019 | 89 | 2014-2018 | RC, single center | China | 46 (51.7) | Median, 24 (range, 1-71) | NA | NA | 82 (92.1) | To assess the risk factors for mortality among patients with KPB |
| Liu, Q., 2019 | 51 | 2012-2016 | RC, single center | China | 30 (58.8) | 51.8 +-13.9 | NA | NA | NA | To evaluate the clinical outcomes of polymicrobial bacteremia involving KP |
| Medeiros, 2019 | 82 | 2015-2016 | RC, single center | Brazil | 53 (64.6) | 57.6+-1  7 | 50 (61.0) | KPC-KP | NA | To evaluate risk factors for 30-day mortality in monomicrobial KPC-KP BSI |
| Menekşe, 2019 | 210 | 2011-2017 | RC, single center | Turkey | 116 (55.2) | 30-day mortality, 61+-15 | 182 (86.7) | CRKP | NA | To describe the effect of colistin resistance on fatality among patients with KP |
| Namikawa, 2019 | 129 | 2012-2018 | RCC, single center | Japan | 80 (62.0) | Non-survivors, 66.2 +- 11.1 | NA | NA | 34 (26.4) | To investigate clinical variables and virulence factors associated with the 30-day mortality of KPB |
| Reid, 2019 | 113 | 2010-2017 | RC, single center | Canada | 62 (54.9) | 72.4 (62.4-81.3) | NA | NA | 23 (20.4) | To determine the incidence and risk factors for acquiring KPB |
| Sun, 2019 | 86 | 2017-2018 | RC, single center | China | 62 (72.1) | 57.3+-14.5 | NA | CRKP | NA | To evaluate the efficacy and safety of different antimicrobial regimens in patients with CRKP BSI |
| Surgers, 2019 | 69 | 2012-2013 | RCS, | France | 68, (98.6) | 64 (56-80) | NA | ESBL-KP | 56 (81.2) | To genetically characterize two *Enterobacteriaceae* most frequently established with carrying ESBL genes, *E. coli* and KP |
| Tan, 2019 | 129 | 2010-2011 | RC, single center | Singapore | 73 (56.6) | 69 (range, 22-100) | NA | NA | NA | To determine if any microbiological marker was associated with primary liver abscess. |
| Chang, 2020 | 285 | 2014-2018 | RC, single center | China | 184 (64.6) | 62 (50, 74) | 59 (20.7) | CRKP vs CSKP | NA | To pinpoint the risk factors of infection and mortality in patients with CRKP and KPB |
| Changpradub, 2020 | 96 | 2016-2018 | RC, single center | Thailand | 62 (64.6) | median: 66 years | 41 (42.7) | Non-CRKP vs CRKP vs ColR KP | NA | To investigate the situation of ColR KP problem |
| Chen, 2020 | 150 | 2014-2016 | RC, single center | China Taiwan | 92 (61.3) | >=65,62/150 | NA | NA | 88 (58.7) | To better understand the correlation of clinical presentation and microbiological characteristics of KPB |
| Cheng, 2020 | 94 | 2014-2019 | RC, single center | China | 54 (57.4) | 1.67 (0.41-9.08) | 24 (25.5) | NA | 43 (45.7) | To assess the risk factors of in-hospital mortality and septic shock incidence in children  with KPB |
| Falcone, 2020 | 102 | 2015-2018 | RC, two hospitals | Italy | 65 (63.7) | 64 (53-74) | 102 (100.0) | KPC-KP | 89 (87.3) | To assess the relationship between the time to appropriate antibiotic therapy in ICU patients with KPC-KP BSIs |
| Hong, K. W.,2020 | 160 | 2008-2017 | RC, single center | Korea | 111 (69.4) | 71 (58.5, 81.0) | 5 (3.1) | NA | 39 (24.4) | To compare the clinical characteristics and outcomes of patients with *Raoultella*  *spp.* and KP BSI |
| Hong, M. Y., 2020 | 258 | 2012-2015 | RC, single center | China Taiwan | 169 (65.5) | >=65,152/258 | NA | NA | NA | To identify patients with community-onset KPB who are at risk for abscess occurrences |
| Huang, 2020 | 444 | 2007, 2017 | PC, single center | China Taiwan | 274 (61.7) | 64.0 +-15.4 | 48 (10.8) | NA | NA | To understand the epidemiological changes of patients with KPB |
| Lee, 2020 | 171 | 2010-2015 | RC, single center | China Taiwan | 109 (63.7) | 72(57-82) | NA | CRKP | NA | To assess the clinical outcome, predictors of mortality, of carbapenems for none-carbapenemase-producing CRKP bacteremia |
| Li, S., 2020 | 146 | 2009-2019 | RC, single center | China | 89 (61.0) | 2.7 months (1.0, 10.9) | 103 (70.5) | NA | NA | To identify prognostic predictors of mortality in pediatric patients with KPB |
| Li, Y.,2020 | 492 | 2014-2019 | RCC, single center | China | 305 (62.0) |  | 174 (35.4) | CRKP vs CSKP | NA | To identify the prevalence and risk factors for infection and mortality of CRKP BSI |
| Nham, 2020 | 278 | 2010-2012 | RC, single center | Korea | 166 (59.7) | ESBL, 55.8 | 75 (27.0) | ESBL-KP vs non-ESBL-KP | NA | To evaluate possible risk factors and outcomes of ESBL-KP bacteremia in cancer patients |
| Papadimitriou-Olivgeris, 2020 | 302 | 2010-2019 | RC, single center | Greece | 215 (71.2) | Non-Survivors, 61.6 +-16.3 | 302 (100.0) | CRKP | NA | To evaluate the impact of the tigecycline’s MIC in the outcome of patients with CRKP bacteraemia |
| Shen, 2020 | 99 | 2014-2019 | RC, single center | China | 69 (69.7) | 66.9+-13.9 | NA |  | 45 (45.5) | To investigate related factors that affect the clinical outcomes of adult patients with KPB |
| Xiao, 2020 | 371 | 2013-2015 | RC, single center | China | 262 (70.6) | CRKP, 61.9+-15.2 | 117(31.5) | CRKP vs CSKP | 335 (90.3) | To identify the risk factors for the development of CRKP infection |
| Yuan, 2020 | 239 | 2017-2018 | RCC, single center | China | 164 (68.6) | CRKP, 55±17 | 126 (52.7) | CRKP vs CSKP | NA | To determine the risk factors for CRKP, and the outcomes of CRKP |
| Zhang, G.,2020 | 496 | 2013-2019 | RC, single center | China | 281 (56.7) | 57 (39.0-67.5) | 240 (48.4) | CRKP vs CSKP | NA | To compare the epidemiology of CRKP and CSKP BSI |
| Zhang, S.,2020 | 297 | 2014-2019 | RC, single center | China | 202 (68.0) | 55 +-16 | 127 (42.8) | SKP vs MDR-KP vs XDR-KP | 162 (54.5) | To evaluate the epidemiological characteristics of patients with KP |
| Balkan, 2021 | 82 | 2014-2018 | RCC, single center | Turkey | 50 (61.0) | 61.5+-17.6 | NA | ColR CRKP vs ColS CRKP | NA | To compare the outcomes of patients with BSI due to ColR CRKP versus ColS CRKP |
| Eren, 2021 | 82 | 2013-2017 | RC, single center | Turkey | 48 (58.5) | Median, 54.5 years | 46 (56.1) | CRKP | NA | To assess risk factors for the mortality due to CRKP BSI |
| Hsu, 2021 | 108 | 2017-2019 | PCC, single center | China Taiwan | 69 (63.9) | CRKP, 66.84 (58.74-75.39) | NA | CRKP vs CSKP | NA | To focus on the analysis of the factors associated with CRKP with or without KPC monomicrobial BSI |
| Lee, I. R., 2021 | 284 | 2016 | RC, multicenter | Singapore | 172 (60.6) | 70 (IQR 61-80) | 28 (9.9) | NA | NA | To investigate the outcomes in KPB patients treated with cefazolin versus ceftriaxone |
| Lee, N. Y., 2021 | 114 | 2010-2015 | RC, single center | China Taiwan | 76 (66.7) | 69 (IQR, 52-78) | NA | CRKP | NA | To explore the clinical potential of cefepime therapy for CRKP BSI |
| Liu, 2021 | 89 | 2014-2017 | RC, single center | China Taiwan | 53 (59.6) | 75.6 (63.8-83.7) | NA | CRKP | NA | To identify the appropriate antimicrobial regimens and risk factors of mortality for CRKP BSI |
| Man, 2021 | 984 | 2009-2017 | RC, single center | China Hong Kong | 581 (59.0) | 75 (63-83) | 205 (20.8) | NA | 220 (22.4) | To evaluate the impact of appropriate empirical antibiotics on outcomes in patients with KPB |
| Papadimitriou-Olivgeris-1，2021 | 384 | 2010-2019 | RC, single center | Greece | 276 (71.9) | 55.7 +-17.3 | 384 (100.0) | KPC-KP | NA | To validate the INCREMENT-CPE score in patients with CPKP BSI |
| Papadimitriou-Olivgeris-2，2021 | 115 | 2010-2019 | RC, single center | Greece | 75 (65.2) | Non-Survivors, 62.1+-15.4 | 115 (100.0) | PDR-KP | NA | To describe the clinical characteristics, therapeutic management, and clinical outcome of BSI due to PDR KP |
| Perez, 2021 | 5712 | 2008-2018 | RC, multicenter | USA | NA | 70+-12 | NA | NA | NA | To demonstrate the use of The Desirability of Outcome Ranking Approach as an approach to evaluate antibiotic treatment in patients with *E. coli* and KP |
| Rodriguez, 2021 | 202 | 2017-2018 | RC, single center | Spain | 135 (66.8) | >70, 86/202 | NA | CRKP vs CSKP | 122 (60.4) | To evaluate the risk factors associated with mortality for patients with bacteremia due to OXA-48 carbapenemase-producing KP |
| Shen, 2021 | 89 | 2018 | RC, single center | China | 65 (73.0) | 55.2+-18.1 | 48 (53.9) | CRKP | NA | To identify risk factors of death due to CRKP |
| Song, 2021 | 704 | 2013-2018 | RC, single center | China | 508 (72.2) | 60.3+-15.3 | 388 (55.1) | NA | NA | To investigate the differences of clinical characteristics, risk factors, and outcomes between polymicrobial KPB and monomicrobial KPB |
| Sung, 2021 | 299 | 2010-2016 | RC, single center | Korea | 170 (56.9) | 69.5+-14.1 | 37 (12.4) | NA | NA | To compare antimicrobial resistance and clinical features in community-onset *E. coli* and KP BSI |
| Tsai, 2021 | 203 | 2010-2016 | RC, single center | China Taiwan | 129 (63.5) | 71 (IQR, 58-80) | NA | CRKP | NA | To assess the clinical outcome, predictors of mortality in patients with CRKP BSI |
| Wu, 2021 | 79 | 2016-2019 | RC, single center | China | 48 (60.8) | 67.0 (59.0-77.5) | NA | NA | NA | To evaluate the clinical variables and genetic backgrounds associated with mortality in patients with KP BSI |
| Aslan, 2022 | 124 | 2014-2018 | RC, single center | Turkey | 66 (53.2) | 62.1+-18.0 | 70 (56.5) | CRKP | 115 (92.7) | To describe the clinical characteristics of BSI due to CRKP in an OXA-48-endemic country |
| Cao, 2022 | 277 | 2016-2020 | RC, single center | China | NA | CRKP, 51.5 (37.0-64.0) | NA | NA | 171 (61.7) | To identify risk factors for hospital acquired CRKP BSI |
| Yingsha Chen, 2022 | 252 | 2015-2020 | RC, single center | China | 176 (69.8) | Mean, 52.7 | 43 (17.1) | CRKP vs CSKP | NA | To establish nomograms to predict the probability of CRKP BSI |
| Liu, 2022 | 447 | 2018-2020 | RC, single center | China | 287 (64.2) | 62+-13.3 | 122 (27.3) | CRKP vs CSKP | NA | To assess the distribution of resistance and sequence types in patients with CRKP BSI |
| Wu, 2022 | 134 | 2017-2020 | RC, single center | China | 92 (68.7) | 66 (55, 73) | 72 (53.7) | CRKP | NA | To evaluate neutrophil-to-lymphocyte ratio as a predictor of the prognosis of these patients with CRKP BSI |
| Ying, 2022 | 72 | 2016-2020 | RC, single center | China | 60 (83.3) | 63.9+-14.2 | NA | CRKP | 72 (100.0) | To explore the relationship between CT-quantified body composition, subcutaneous adipose tissue, and skeletal muscle in patients with CRKP BSI |
| Ang, 2022 | 260 | 2016-2019 | RC, single center | Malaysia | 139 (53.5) | Death group, 68+-12 | 17 (6.5) | NA | 5 (1.9) | To determine the poor prognostic factors and predictors of mortality from KPB |
| Atalay, 2022 | 111 | 2018-2019 | RC, single center | Turkey | 69 (62.2) | 57.61+-19.98 | NA | CRKP | NA | To determine the effect of dual carbapenem therapy on mortality in patients with CRKP bacteremia |
| Yili Chen, 2022 | 252 | 2011-2020 | RC, single center | China | 166 (65.9) | 60-70, 138/252 (54.8) | 48 (19.0) | CRKP vs CSKP | NA | To assess risk factors associated with CRKP BSI and mortality among elderly patients |
| Liang, 2022 | 103 | 2010-2018 | RC, multicenter | China | 73 (70.9) | CRKP, 27 (IQR, 0-61) | 37 (35.9) | CRKP vs CSKP | 84 (81.6) | To compare antimicrobial resistance, virulence, and risk factors between CRKP and CSKP from patients with BSI |
| Mairi, 2022 | 328 | 2019- 2020 | PC, single center | Algeria | 207 (63.1) | Age at admission <=24 hours, 308/328 | 328 (100.0) | NA | NA | To characterize by whole genome sequencing a collection of ESBL-Kp strains isolated from neonatal BSI |
| Onorato, 2022 | 154 | 2020 | RC, single center | Italy | 97 (63.0) | 60.8+-18.9 | 78 (50.6) | KPC/OXA-48 vs non-carbapenemase detected | 136 (88.3) | To evaluate the clinical and microbiological characteristics of patients with BSI due to CRE |
| Sheng, 2022 | 66 | 2019-2020 | RC, single center | China | 39 (59.1) | 63 (IQR, 50-74) | 20 (30.3) | NA | 18 (27.3) | To investigate the clinical and molecular characteristics of patients with KPB |
| Soares de Moraes, 2022 | 107 | 2015-2018 | RC, single center | Brazil | 65 (60.7) | 52.0+-22.1 | 60 (56.1) | ESBL-KP vs CRKP | NA | To evaluate the risk factors, mortality and molecularly characterize KPC-KP BSI |
| Zhang, 2022 | 119 | 2019-2021 | RC, single center | China | 83 (69.7) | 51.6+-16.9 | 47 (39.5) | NA | NA | To look at the correlation of the type VI secretion system in KP pathogenicity and antibiotic resistance |
| Lima, 2022 | 76 | 2015-2019 | RC, single center | Spain | 53 (69.7) | Median, 61 (IQR, 46-76) | NA | CRKP | 1 (0.1) | To analyze clinical success of CAZ-AVI compared with best available therapy in patients with OXA-48 type CRKP BSI |
| Meng, 2022 | 129 | 2018-2021 | RC, single center | China | 85 (65.9) | 46.1+-13.8 | 93 (72.1) | CRKP | NA | To identify risk factors for mortality and outcomes in HMs patients with CRKP BSI |

AB, Acinetobacter baumannii; BSI, bloodstream infections; CA, community-acquired; CAZ-AVI, Ceftazidime-Avibactam; CAZ-R, Ceftazidime- resistant; ColR, colistin-resistant; ColS, colistin-susceptible; CRKP, carbapenem-resistant *Klebsiella pneumoniae*; CRE, carbapenem-resistant *Enterobacteriaceae*; CSKP, carbapenem-susceptible *Klebsiella pneumoniae*; E. coli, Escherichia coli; ESBL, extended-spectrum β-Lactamase-positive;ETP-NS, ertapenem non-susceptible *Klebsiella pneumoniae*; ETP-S, ertapenem susceptible *Klebsiella pneumoniae*; 3GCRKP, third generation-cephalosporin-resistant *Klebsiella pneumoniae*; HA, hospital-acquired; HCA, healthcare-associated; HMs, hematological malignancies; hvKP, hypervirulent *Klebsiella pneumoniae*; ICU, intensive care unit; KP, *Klebsiella pneumoniae*; KPB, *Klebsiella pneumoniae* bacteremia; KPC, *Klebsiella pneumoniae* carbapenemase; MDR, multidrug-resistant; MIC, minimum inhibitory concentration; NA, not available; NDM, New Delhi Metallo-β-lactamase; PC, prospective cohort; PCC, prospective case-control; PCS, prospective cross-sectional; PDR, pandrug-resistant; RC, retrospective cohort; RCC, retrospective case-control; SKP, susceptible *Klebsiella pneumoniae ;* TCG-S, tigecycline susceptible; TCG-R, tigecycline resistant.

**Text S2 References of all included studies ^1-157^**.

1. Pena, C.; Pujol, M.; Ardanuy, C.; Ricart, A.; Pallares, R.; Linares, J.; Ariza, J.; Gudiol, F., An outbreak of hospital-acquired *Klebsiella pneumoniae*  bacteraemia, including strains producing extended-spectrum beta-lactamase. *Journal of Hospital Infection* **2001,** *47* (1), 53-59.

2. Kim, B. N.; Woo, J. H.; Kim, M. N.; Ryu, J.; Kim, Y. S., Clinical implications of extended-spectrum beta-lactamase-producing *Klebsiella pneumoniae*  bacteraemia. *The Journal of hospital infection* **2002,** *52* (2), 99-106.

3. Tsay, R. W.; Siu, L. K.; Fung, C. P.; Chang, F. Y., Characteristics of bacteremia between community-acquired and nosocomial *Klebsiella pneumoniae*  infection: risk factor for mortality and the impact of capsular serotypes as a herald for community-acquired infection. *Arch Intern Med* **2002,** *162* (9), 1021-7.

4. Paterson, D. L.; Ko, W. C.; Von Gottberg, A.; Mohapatra, S.; Casellas, J. M.; Goossens, H.; Mulazimoglu, L.; Trenholme, G.; Klugman, K. P.; Bonomo, R. A.; Rice, L. B.; Wagener, M. M.; McCormack, J. G.; Yu, V. L., Antibiotic therapy for *Klebsiella pneumoniae*  bacteremia: implications of production of extended-spectrum beta-lactamases. *Clin Infect Dis* **2004,** *39* (1), 31-7.

5. Anderson, D. J.; Engemann, J. J.; Harrell, L. J.; Carmeli, Y.; Reller, L. B.; Kaye, K. S., Predictors of mortality in patients with bloodstream infection due to ceftazidime-resistant *Klebsiella pneumoniae* . *Antimicrobial agents and chemotherapy* **2006,** *50* (5), 1715-20.

6. Kang, C. I.; Kim, S. H.; Bang, J. W.; Kim, H. B.; Kim, N. J.; Kim, E. C.; Oh, M. D.; Choe, K. W., Community-acquired versus nosocomial *Klebsiella pneumoniae*  bacteremia: clinical features, treatment outcomes, and clinical implication of antimicrobial resistance. *J Korean Med Sci* **2006,** *21* (5), 816-22.

7. Marra, A. R.; Wey, S. B.; Castelo, A.; Gales, A. C.; Cal, R. G.; Filho, J. R.; Edmond, M. B.; Pereira, C. A., Nosocomial bloodstream infections caused by *Klebsiella pneumoniae* : impact of extended-spectrum beta-lactamase (ESBL) production on clinical outcome in a hospital with high ESBL prevalence. *BMC Infect Dis* **2006,** *6*, 24.

8. Robenshtok, E.; Paul, M.; Leibovici, L.; Fraser, A.; Pitlik, S.; Ostfeld, I.; Samra, Z.; Perez, S.; Lev, B.; Weinberger, M., The significance of Acinetobacter baumannii bacteraemia compared with *Klebsiella pneumoniae*  bacteraemia: risk factors and outcomes. *The Journal of hospital infection* **2006,** *64* (3), 282-7.

9. Tumbarello, M.; Spanu, T.; Sanguinetti, M.; Citton, R.; Montuori, E.; Leone, F.; Fadda, G.; Cauda, R., Bloodstream infections caused by extended-spectrum-beta-lactamase-producing *Klebsiella pneumoniae* : risk factors, molecular epidemiology, and clinical outcome. *Antimicrobial agents and chemotherapy* **2006,** *50* (2), 498-504.

10. Mosqueda-Gómez, J. L.; Montaño-Loza, A.; Rolón, A. L.; Cervantes, C.; Bobadilla-del-Valle, J. M.; Silva-Sánchez, J.; Garza-Ramos, U.; Villasís-Keever, A.; Galindo-Fraga, A.; Palacios, G. M.; Ponce-de-León, A.; Sifuentes-Osornio, J., Molecular epidemiology and risk factors of bloodstream infections caused by extended-spectrum beta-lactamase-producing *Klebsiella pneumoniae*  A case-control study. *Int J Infect Dis* **2008,** *12* (6), 653-9.

11. Daikos, G. L.; Petrikkos, P.; Psichogiou, M.; Kosmidis, C.; Vryonis, E.; Skoutelis, A.; Georgousi, K.; Tzouvelekis, L. S.; Tassios, P. T.; Bamia, C.; Petrikkos, G., Prospective observational study of the impact of VIM-1 metallo-beta-lactamase on the outcome of patients with *Klebsiella pneumoniae*  bloodstream infections. *Antimicrobial agents and chemotherapy* **2009,** *53* (5), 1868-73.

12. Liao, C. H.; Lai, C. C.; Hsu, M. S.; Huang, Y. T.; Chu, F. Y.; Hsu, H. S.; Hsueh, P. R., Correlation between time to positivity of blood cultures with clinical presentation and outcomes in patients with *Klebsiella pneumoniae*  bacteraemia: prospective cohort study. *Clin Microbiol Infect* **2009,** *15* (12), 1119-25.

13. Meatherall, B. L.; Gregson, D.; Ross, T.; Pitout, J. D.; Laupland, K. B., Incidence, risk factors, and outcomes of *Klebsiella pneumoniae*  bacteremia. *Am J Med* **2009,** *122* (9), 866-73.

14. Szilágyi, E.; Füzi, M.; Böröcz, K.; Kurcz, A.; Tóth, A.; Nagy, K., Risk factors and outcomes for bloodstream infections with extended-spectrum beta -lactamase-producing *Klebsiella pneumoniae*  ; Findings of the nosocomial surveillance system in Hungary. *Acta microbiologica et immunologica Hungarica* **2009,** *56* (3), 251-62.

15. Mouloudi, E.; Protonotariou, E.; Zagorianou, A.; Iosifidis, E.; Karapanagiotou, A.; Giasnetsova, T.; Tsioka, A.; Roilides, E.; Sofianou, D.; Gritsi-Gerogianni, N., Bloodstream infections caused by metallo-β-lactamase/*Klebsiella pneumoniae*  carbapenemase-producing K. pneumoniae among intensive care unit patients in Greece: risk factors for infection and impact of type of resistance on outcomes. *Infection control and hospital epidemiology* **2010,** *31* (12), 1250-6.

16. Pépin, J.; Yared, N.; Alarie, I.; Lanthier, L.; Vanasse, A.; Tessier, P.; Deveau, J.; Chagnon, M. N.; Comeau, R.; Cotton, P.; Libby, S. J.; Valiquette, L., *Klebsiella pneumoniae*  bacteraemia in a region of Canada. *Clin Microbiol Infect* **2010,** *16* (2), 141-146.

17. Tsai, S. S.; Huang, J. C.; Chen, S. T.; Sun, J. H.; Wang, C. C.; Lin, S. F.; Hsu, B. R.; Lin, J. D.; Huang, S. Y.; Huang, Y. Y., Characteristics of *Klebsiella pneumoniae*  bacteremia in community-acquired and nosocomial infections in diabetic patients. *Chang Gung medical journal* **2010,** *33* (5), 532-9.

18. Francisco Tuon, F.; Kruger, M.; Terreri, M.; Penteado-Filho, S. R.; Gortz, L., Klebsiella ESBL bacteremia-mortality and risk factors. *Brazilian Journal of Infectious Diseases* **2011,** *15* (6), 594-598.

19. Lee, J. A.; Kang, C. I.; Joo, E. J.; Ha, Y. E.; Kang, S. J.; Park, S. Y.; Chung, D. R.; Peck, K. R.; Ko, K. S.; Lee, N. Y.; Song, J. H., Epidemiology and clinical features of community-onset bacteremia caused by extended-spectrum β-lactamase-producing *Klebsiella pneumoniae* . *Microb Drug Resist* **2011,** *17* (2), 267-73.

20. Lin, Y. T.; Liu, C. J.; Fung, C. P.; Tzeng, C. H., Nosocomial *Klebsiella pneumoniae*  bacteraemia in adult cancer patients--characteristics of neutropenic and non-neutropenic patients. *Scandinavian journal of infectious diseases* **2011,** *43* (8), 603-8.

21. Neuner, E. A.; Yeh, J. Y.; Hall, G. S.; Sekeres, J.; Endimiani, A.; Bonomo, R. A.; Shrestha, N. K.; Fraser, T. G.; van Duin, D., Treatment and outcomes in carbapenem-resistant *Klebsiella pneumoniae*  bloodstream infections. *Diagnostic microbiology and infectious disease* **2011,** *69* (4), 357-62.

22. Webster, D. P.; Young, B. C.; Morton, R.; Collyer, D.; Batchelor, B.; Turton, J. F.; Maharjan, S.; Livermore, D. M.; Bejon, P.; Cookson, B. D.; Bowler, I., Impact of a clonal outbreak of extended-spectrum beta-lactamase-producing *Klebsiella pneumoniae*  in the development and evolution of bloodstream infections by K. pneumoniae and Escherichia coli: an 11 year experience in Oxfordshire, UK. *Journal of Antimicrobial Chemotherapy* **2011,** *66* (9), 2126-2135.

23. Zarkotou, O.; Pournaras, S.; Tselioti, P.; Dragoumanos, V.; Pitiriga, V.; Ranellou, K.; Prekates, A.; Themeli-Digalaki, K.; Tsakris, A., Predictors of mortality in patients with bloodstream infections caused by KPC-producing *Klebsiella pneumoniae*  and impact of appropriate antimicrobial treatment. *Clin Microbiol Infect* **2011,** *17* (12), 1798-803.

24. Ben-David, D.; Kordevani, R.; Keller, N.; Tal, I.; Marzel, A.; Gal-Mor, O.; Maor, Y.; Rahav, G., Outcome of carbapenem resistant *Klebsiella pneumoniae*  bloodstream infections. *Clin Microbiol Infect* **2012,** *18* (1), 54-60.

25. Jung, Y.; Lee, M. J.; Sin, H. Y.; Kim, N. H.; Hwang, J. H.; Park, J.; Choe, P. G.; Park, W. B.; Kim, E. S.; Park, S. W.; Park, K. U.; Kim, H. B.; Kim, N. J.; Kim, E. C.; Song, K. H.; Oh, M. D., Differences in characteristics between healthcare-associated and community-acquired infection in community-onset *Klebsiella pneumoniae*  bloodstream infection in Korea. *BMC Infect Dis* **2012,** *12*, 239.

26. Lee, N. Y.; Wu, J. J.; Lin, S. H.; Ko, W. C.; Tsai, L. H.; Yan, J., Characterization of carbapenem-nonsusceptible *Klebsiella pneumoniae*  bloodstream isolates at a Taiwanese hospital: clinical impacts of lowered breakpoints for carbapenems. *European Journal of Clinical Microbiology & Infectious Diseases* **2012,** *31* (8), 1941-1950.

27. Liu, S. W.; Chang, H. J.; Chia, J. H.; Kuo, A. J.; Wu, T. L.; Lee, M. H., Outcomes and characteristics of ertapenem-nonsusceptible *Klebsiella pneumoniae*  bacteremia at a university hospital in Northern Taiwan: a matched case-control study. *Journal of microbiology, immunology, and infection = Wei mian yu gan ran za zhi* **2012,** *45* (2), 113-9.

28. Tumbarello, M.; Viale, P.; Viscoli, C.; Trecarichi, E. M.; Tumietto, F.; Marchese, A.; Spanu, T.; Ambretti, S.; Ginocchio, F.; Cristini, F.; Losito, A. R.; Tedeschi, S.; Cauda, R.; Bassetti, M., Predictors of mortality in bloodstream infections caused by *Klebsiella pneumoniae*  carbapenemase-producing K. pneumoniae: importance of combination therapy. *Clin Infect Dis* **2012,** *55* (7), 943-50.

29. Wu, H. S.; Wang, F. D.; Tseng, C. P.; Wu, T. H.; Lin, Y. T.; Fung, C. P., Characteristics of healthcare-associated and community-acquired *Klebsiella pneumoniae*  bacteremia in Taiwan. *J Infect* **2012,** *64* (2), 162-8.

30. Hussein, K.; Raz-Pasteur, A.; Finkelstein, R.; Neuberger, A.; Shachor-Meyouhas, Y.; Oren, I.; Kassis, I., Impact of carbapenem resistance on the outcome of patients' hospital-acquired bacteraemia caused by *Klebsiella pneumoniae* . *The Journal of hospital infection* **2013,** *83* (4), 307-13.

31. Kang, C. K.; Kim, E. S.; Song, K. H.; Kim, H. B.; Kim, T. S.; Kim, N. H.; Kim, C. J.; Choe, P. G.; Bang, J. H.; Park, W. B.; Park, K. U.; Park, S. W.; Kim, N. J.; Kim, E. C.; Oh, M. D., Can a routine follow-up blood culture be justified in *Klebsiella pneumoniae*  bacteremia? a retrospective case-control study. *Bmc Infectious Diseases* **2013,** *13*.

32. Tseng, C. P.; Wu, H. S.; Wu, T. H.; Lin, Y. T.; Fung, C. P., Clinical characteristics and outcome of patients with community-onset *Klebsiella pneumoniae*  bacteremia requiring intensive care. *Journal of microbiology, immunology, and infection = Wei mian yu gan ran za zhi* **2013,** *46* (3), 217-223.

33. Chetcuti Zammit, S.; Azzopardi, N.; Sant, J., Mortality risk score for *Klebsiella pneumoniae*  bacteraemia. *European journal of internal medicine* **2014,** *25* (6), 571-6.

34. Daikos, G. L.; Tsaousi, S.; Tzouvelekis, L. S.; Anyfantis, I.; Psichogiou, M.; Argyropoulou, A.; Stefanou, I.; Sypsa, V.; Miriagou, V.; Nepka, M.; Georgiadou, S.; Markogiannakis, A.; Goukos, D.; Skoutelis, A., Carbapenemase-producing *Klebsiella pneumoniae*  bloodstream infections: lowering mortality by antibiotic combination schemes and the role of carbapenems. *Antimicrobial agents and chemotherapy* **2014,** *58* (4), 2322-8.

35. Gallagher, J. C.; Kuriakose, S.; Haynes, K.; Axelrod, P., Case-Case-Control Study of Patients with Carbapenem-Resistant and Third-Generation-Cephalosporin-Resistant *Klebsiella pneumoniae*  Bloodstream Infections. *Antimicrobial agents and chemotherapy* **2014,** *58* (10), 5732-5735.

36. Giannella, M.; Trecarichi, E. M.; De Rosa, F. G.; Del Bono, V.; Bassetti, M.; Lewis, R. E.; Losito, A. R.; Corcione, S.; Saffioti, C.; Bartoletti, M.; Maiuro, G.; Cardellino, C. S.; Tedeschi, S.; Cauda, R.; Viscoli, C.; Viale, P.; Tumbarello, M., Risk factors for carbapenem-resistant *Klebsiella pneumoniae*  bloodstream infection among rectal carriers: a prospective observational multicentre study. *Clinical Microbiology and Infection* **2014,** *20* (12), 1357-1362.

37. Girometti, N.; Lewis, R. E.; Giannella, M.; Ambretti, S.; Bartoletti, M.; Tedeschi, S.; Tumietto, F.; Cristini, F.; Trapani, F.; Gaibani, P.; Viale, P., *Klebsiella pneumoniae*  bloodstream infection: epidemiology and impact of inappropriate empirical therapy. *Medicine* **2014,** *93* (17), 298-309.

38. Gurntke, S.; Kohler, C.; Steinmetz, I.; Pfeifer, Y.; Eller, C.; Gastmeier, P.; Schwab, F.; Leistner, R., Molecular epidemiology of extended-spectrum beta-lactamase (ESBL)-positive *Klebsiella pneumoniae*  from bloodstream infections and risk factors for mortality. *Journal of Infection and Chemotherapy* **2014,** *20* (12), 817-819.

39. Li, D.; Chen, Y.; Zhang, W.; Zheng, S.; Zhang, Q.; Bai, C.; Zhang, P., Risk factors for hospital-acquired bloodstream infections caused by extended-spectrum β-lactamase *Klebsiella pneumoniae*  among cancer patients. *Irish journal of medical science* **2014,** *183* (3), 463-9.

40. Liu, Y. M.; Bin, B. B.; Zhang, Y. Y.; Zhang, W.; Shen, H.; Li, H.; Cao, B., Clinical and Molecular Characteristics of Emerging Hypervirulent *Klebsiella pneumoniae*  Bloodstream Infections in Mainland China. *Antimicrobial agents and chemotherapy* **2014,** *58* (9), 5379-5385.

41. Papadimitriou-Olivgeris, M.; Marangos, M.; Christofidou, M.; Fligou, F.; Bartzavali, C.; Panteli, E. S.; Vamvakopoulou, S.; Filos, K. S.; Anastassiou, E. D., Risk factors for infection and predictors of mortality among patients with KPC-producing *Klebsiella pneumoniae*  bloodstream infections in the intensive care unit. *Scandinavian journal of infectious diseases* **2014,** *46* (9), 642-8.

42. Yang, C. C.; Wu, C. H.; Lee, C. T.; Liu, H. T.; Chen, J. B.; Chiu, C. H.; Chen, C. H.; Chuang, F. R., Nosocomial extended-spectrum beta-lactamase-producing *Klebsiella pneumoniae*  bacteremia in hemodialysis patients and the implications for antibiotic therapy. *Int J Infect Dis* **2014,** *28*, 3-7.

43. Alicino, C.; Giacobbe, D. R.; Orsi, A.; Tassinari, F.; Trucchi, C.; Sarteschi, G.; Copello, F.; Del Bono, V.; Viscoli, C.; Icardi, G., Trends in the annual incidence of carbapenem-resistant *Klebsiella pneumoniae*  bloodstream infections: a 8-year retrospective study in a large teaching hospital in northern Italy. *BMC Infect Dis* **2015,** *15*, 415.

44. Chang, E. K.; Kao, K. L.; Tsai, M. S.; Yang, C. J.; Huang, Y. T.; Liu, C. Y.; Liao, C. H., Occult *Klebsiella pneumoniae*  bacteremia at emergency department: A single center experience. *Journal of microbiology, immunology, and infection = Wei mian yu gan ran za zhi* **2015,** *48* (6), 684-91.

45. Cheng, C.; Lin, Y. X.; Li, J. J.; Zhang, Z. J., Risk factors for multidrug-resistant *Klebsiella pneumoniae*  sepsis in children. *Zhongguo dang dai er ke za zhi = Chinese journal of contemporary pediatrics* **2015,** *17* (9), 932-6.

46. Giacobbe, D. R.; Del Bono, V.; Trecarichi, E. M.; De Rosa, F. G.; Giannella, M.; Bassetti, M.; Bartoloni, A.; Losito, A. R.; Corcione, S.; Bartoletti, M.; Mantengoli, E.; Saffioti, C.; Pagani, N.; Tedeschi, S.; Spanu, T.; Rossolini, G. M.; Marchese, A.; Ambretti, S.; Cauda, R.; Viale, P.; Viscoli, C.; Tumbarello, M., Risk factors for bloodstream infections due to colistin-resistant KPC-producing *Klebsiella pneumoniae* : results from a multicenter case-control-control study. *Clin Microbiol Infect* **2015,** *21* (12), 1106.e1-8.

47. Gomez-Simmonds, A.; Greenman, M.; Sullivan, S. B.; Tanner, J. P.; Sowash, M. G.; Whittier, S.; Uhlemann, A. C., Population Structure of *Klebsiella pneumoniae*  Causing Bloodstream Infections at a New York City Tertiary Care Hospital: Diversification of Multidrug-Resistant Isolates. *J Clin Microbiol* **2015,** *53* (7), 2060-7.

48. Gonzalez-Padilla, M.; Torre-Cisneros, J.; Rivera-Espinar, F.; Pontes-Moreno, A.; López-Cerero, L.; Pascual, A.; Natera, C.; Rodríguez, M.; Salcedo, I.; Rodríguez-López, F.; Rivero, A.; Rodríguez-Baño, J., Gentamicin therapy for sepsis due to carbapenem-resistant and colistin-resistant *Klebsiella pneumoniae* . *J Antimicrob Chemother* **2015,** *70* (3), 905-13.

49. Huang, C. H.; Tsai, J. S.; Chen, I. W.; Hsu, B. R.; Huang, M. J.; Huang, Y. Y., Risk factors for in-hospital mortality in patients with type 2 diabetes complicated by community-acquired *Klebsiella pneumoniae*  bacteremia. *Journal of the Formosan Medical Association = Taiwan yi zhi* **2015,** *114* (10), 916-22.

50. Malaki, M., *Klebsiella pneumoniae*  Bacteremia Presenting on a Neonatal Intensive Care Unit during the First Week of Life. *Journal of Pediatric Infectious Diseases* **2015,** *10* (1), 12-15.

51. Pau, C. K.; Ma, F. F.; Ip, M.; You, J. H., Characteristics and outcomes of *Klebsiella pneumoniae*  bacteraemia in Hong Kong. *Infect Dis (Lond)* **2015,** *47* (5), 283-8.

52. Togawa, A.; Toh, H.; Onozawa, K.; Yoshimura, M.; Tokushige, C.; Shimono, N.; Takata, T.; Tamura, K., Influence of the bacterial phenotypes on the clinical manifestations in *Klebsiella pneumoniae*  bacteremia patients: A retrospective cohort study. *Journal of infection and chemotherapy : official journal of the Japan Society of Chemotherapy* **2015,** *21* (7), 531-7.

53. Brady, M.; Cunney, R.; Murchan, S.; Oza, A.; Burns, K., *Klebsiella pneumoniae*  bloodstream infection, antimicrobial resistance and consumption trends in Ireland: 2008 to 2013. *Eur J Clin Microbiol Infect Dis* **2016,** *35* (11), 1777-1785.

54. Buys, H.; Muloiwa, R.; Bamford, C.; Eley, B., *Klebsiella pneumoniae*  bloodstream infections at a South African children's hospital 2006-2011, a cross-sectional study. *BMC Infect Dis* **2016,** *16* (1), 570.

55. Durdu, B.; Hakyemez, I. N.; Bolukcu, S.; Okay, G.; Gultepe, B.; Aslan, T., Mortality markers in nosocomial *Klebsiella pneumoniae*  bloodstream infection. *SpringerPlus* **2016,** *5* (1), 1892.

56. Fraenkel-Wandel, Y.; Raveh-Brawer, D.; Wiener-Well, Y.; Yinnon, A. M.; Assous, M. V., Mortality due to blaKPC *Klebsiella pneumoniae*  bacteraemia. *J Antimicrob Chemother* **2016,** *71* (4), 1083-7.

57. Gao, Z. F.; Cui, W.; Lei, J. E.; Ma, C., Clinical study of Klebsiella bacteraemia in type 2 diabetic patients. *Journal of Xi'an Jiaotong University (Medical Sciences)* **2016,** *37* (6), 835-840.

58. Gomez-Simmonds, A.; Nelson, B.; Eiras, D. P.; Loo, A.; Jenkins, S. G.; Whittier, S.; Calfee, D. P.; Satlin, M. J.; Kubin, C. J.; Furuya, E. Y., Combination Regimens for Treatment of Carbapenem-Resistant *Klebsiella pneumoniae*  Bloodstream Infections. *Antimicrobial agents and chemotherapy* **2016,** *60* (6), 3601-7.

59. Juan, C. H.; Huang, Y. W.; Lin, Y. T.; Yang, T. C.; Wang, F. D., Risk Factors, Outcomes, and Mechanisms of Tigecycline-Nonsusceptible *Klebsiella pneumoniae*  Bacteremia. *Antimicrobial agents and chemotherapy* **2016,** *60* (12), 7357-7363.

60. Li, J.; Tang, C.; Wang, H. J. S.; Hi, K.; Xiao, S.; Deng, A.; Huang, Y.; Xia, Z., Impact of extended-spectrum [&beta;-lactamase on clinical outcome and medical cost in patients with bloodstream infection due to Klebsiella pneumonia. *National Medical Journal of China* **2016,** *96* (24), 1903-1906.

61. Luk, S.; Wong, W. K.; Ho, A. Y.; Yu, K. C.; To, W. K.; Ng, T. K., Clinical features and molecular epidemiology of plasmid-mediated DHA-type AmpC β-lactamase-producing *Klebsiella pneumoniae*  blood culture isolates, Hong Kong. *J Glob Antimicrob Resist* **2016,** *7*, 37-42.

62. Martelius, T.; Jalava, J.; Kärki, T.; Möttönen, T.; Ollgren, J.; Lyytikäinen, O., Nosocomial bloodstream infections caused by Escherichia coli and *Klebsiella pneumoniae*  resistant to third-generation cephalosporins, Finland, 1999-2013: Trends, patient characteristics and mortality. *Infect Dis (Lond)* **2016,** *48* (3), 229-34.

63. Tian, L.; Tan, R.; Chen, Y.; Sun, J.; Liu, J.; Qu, H.; Wang, X., Epidemiology of *Klebsiella pneumoniae*  bloodstream infections in a teaching hospital: factors related to the carbapenem resistance and patient mortality. *Antimicrob Resist Infect Control* **2016,** *5*, 48.

64. Tofas, P.; Skiada, A.; Angelopoulou, M.; Sipsas, N.; Pavlopoulou, I.; Tsaousi, S.; Pagoni, M.; Kotsopoulou, M.; Perlorentzou, S.; Antoniadou, A.; Pirounaki, M.; Skoutelis, A.; Daikos, G. L., Carbapenemase-producing *Klebsiella pneumoniae*  bloodstream infections in neutropenic patients with haematological malignancies or aplastic anaemia: Analysis of 50 cases. *Int J Antimicrob Agents* **2016,** *47* (4), 335-9.

65. Trecarichi, E. M.; Pagano, L.; Martino, B.; Candoni, A.; Di Blasi, R.; Nadali, G.; Fianchi, L.; Delia, M.; Sica, S.; Perriello, V.; Busca, A.; Aversa, F.; Fanci, R.; Melillo, L.; Lessi, F.; Del Principe, M. I.; Cattaneo, C.; Tumbarello, M., Bloodstream infections caused by *Klebsiella pneumoniae*  in onco-hematological patients: clinical impact of carbapenem resistance in a multicentre prospective survey. *American journal of hematology* **2016,** *91* (11), 1076-1081.

66. Basaranoglu, S. T.; Ozsurekci, Y.; Aykac, K.; Oncel, E. K.; Bicakcigil, A.; Sancak, B.; Cengiz, A. B.; Kara, A.; Ceyhan, M., A comparison of blood stream infections with extended spectrum beta-lactamase-producing and non-producing *Klebsiella pneumoniae*  in pediatric patients. *Italian journal of pediatrics* **2017,** *43*.

67. Giannella, M.; Graziano, E.; Marconi, L.; Girometti, N.; Bartoletti, M.; Tedeschi, S.; Tumietto, F.; Cristini, F.; Ambretti, S.; Berlingeri, A.; Lewis, R. E.; Viale, P., Risk factors for recurrent carbapenem resistant *Klebsiella pneumoniae*  bloodstream infection: a prospective cohort study. *Eur J Clin Microbiol Infect Dis* **2017,** *36* (10), 1965-1970.

68. Kaur, A.; Gandra, S.; Gupta, P.; Mehta, Y.; Laxminarayan, R.; Sengupta, S., Clinical outcome of dual colistin- and carbapenem-resistant *Klebsiella pneumoniae*  bloodstream infections: A single-center retrospective study of 75 cases in India. *Am J Infect Control* **2017,** *45* (11), 1289-1291.

69. Kuo, T. H.; Yang, C. Y.; Lee, C. H.; Hsieh, C. C.; Ko, W. C.; Lee, C. C., Propensity score matched analysis comparing the clinical outcome of *Klebsiella pneumoniae*  and Escherichia coli causing community-onset monomicrobial bacteremia. *Medicine* **2017,** *96* (26), e7075.

70. Li, L.; Huang, H., Risk factors of mortality in bloodstream infections caused by Klebsiella pneumonia. *Medicine (United States)* **2017,** *96* (35).

71. Liao, Y.; Hu, G. H.; Xu, Y. F.; Che, J. P.; Luo, M.; Zhang, H. M.; Peng, B.; Yao, X. D.; Zheng, J. H.; Liu, M., Retrospective analysis of fosfomycin combinational therapy for sepsis caused by carbapenem-resistant *Klebsiella pneumoniae* . *Experimental and therapeutic medicine* **2017,** *13* (3), 1003-1010.

72. Machuca, I.; Gutierrez-Gutierrez, B.; Gracia-Ahufinger, I.; Espinar, F. R.; Cano, A.; Guzman-Puche, J.; Perez-Nadales, E.; Natera, C.; Rodriguez, M.; Leon, R.; Caston, J. J.; Rodriguez-Lopez, F.; Rodriguez-Bano, J.; Torre-Cisneros, J., Mortality Associated with Bacteremia Due to Colistin-Resistant *Klebsiella pneumoniae*  with High-Level Meropenem Resistance: Importance of Combination Therapy without Colistin and Carbapenems. *Antimicrobial agents and chemotherapy* **2017,** *61* (8).

73. Papadimitriou-Olivgeris, M.; Fligou, F.; Bartzavali, C.; Zotou, A.; Spyropoulou, A.; Koutsileou, K.; Vamvakopoulou, S.; Sioulas, N.; Karamouzos, V.; Anastassiou, E. D.; Spiliopoulou, I.; Christofidou, M.; Marangos, M., Carbapenemase-producing *Klebsiella pneumoniae*  bloodstream infection in critically ill patients: risk factors and predictors of mortality. *Eur J Clin Microbiol Infect Dis* **2017,** *36* (7), 1125-1131.

74. Shields, R. K.; Nguyen, M. H.; Chen, L.; Press, E. G.; Potoski, B. A.; Marini, R. V.; Doi, Y.; Kreiswirth, B. N.; Clancy, C. J., Ceftazidime-Avibactam Is Superior to Other Treatment Regimens against Carbapenem-Resistant *Klebsiella pneumoniae*  Bacteremia. *Antimicrobial agents and chemotherapy* **2017,** *61* (8).

75. Veeraraghavan, B.; Shankar, C.; Karunasree, S.; Kumari, S.; Ravi, R.; Ralph, R., Carbapenem resistant *Klebsiella pneumoniae*  isolated from bloodstream infection: Indian experience. *Pathogens and global health* **2017,** *111* (5), 240-246.

76. Zhang, Y.; Dong, F.; Song, W.; Guo, L.; Wang, Y.; Liu, G., Clinical characteristics and antimicrobial resistance of the bloodstream infections due to NDM-1 producing *Klebsiella pneumoniae*  in children. *Chinese Journal of Infection and Chemotherapy* **2017,** *17* (5), 516-522.

77. Corcione, S.; Angilletta, R.; Raviolo, S.; Filippini, C.; Fossati, L.; Di Perri, G.; Cavallo, R.; De Rosa, F. G., Epidemiology and risk factors for mortality in bloodstream infection by CP-Kp, ESBL-E, Candida and CDI: A single center retrospective study. *European journal of internal medicine* **2018,** *48*, 44-49.

78. Cristina, M. L.; Alicino, C.; Sartini, M.; Faccio, V.; Spagnolo, A. M.; Bono, V. D.; Cassola, G.; De Mite, A. M.; Crisalli, M. P.; Ottria, G.; Schinca, E.; Pinto, G. L.; Bottaro, L. C.; Viscoli, C.; Orsi, A.; Giacobbe, D. R.; Icardi, G., Epidemiology, management, and outcome of carbapenem-resistant *Klebsiella pneumoniae*  bloodstream infections in hospitals within the same endemic metropolitan area. *J Infect Public Health* **2018,** *11* (2), 171-177.

79. Cubero, M.; Grau, I.; Tubau, F.; Pallarés, R.; Domínguez, M.; Liñares, J.; Ardanuy, C., Molecular Epidemiology of *Klebsiella pneumoniae*  Strains Causing Bloodstream Infections in Adults. *Microb Drug Resist* **2018,** *24* (7), 949-957.

80. Giannella, M.; Trecarichi, E. M.; Giacobbe, D. R.; De Rosa, F. G.; Bassetti, M.; Bartoloni, A.; Bartoletti, M.; Losito, A. R.; Del Bono, V.; Corcione, S.; Tedeschi, S.; Raffaelli, F.; Saffioti, C.; Spanu, T.; Rossolini, G. M.; Marchese, A.; Ambretti, S.; Cauda, R.; Viscoli, C.; Lewis, R. E.; Viale, P.; Tumbarello, M., Effect of combination therapy containing a high-dose carbapenem on mortality in patients with carbapenem-resistant *Klebsiella pneumoniae*  bloodstream infection. *Int J Antimicrob Agents* **2018,** *51* (2), 244-248.

81. Hyun, M.; Noh, C. I.; Ryu, S. Y.; Kim, H. A., Changing trends in clinical characteristics and antibiotic susceptibility of *Klebsiella pneumoniae*  bacteremia. *The Korean journal of internal medicine* **2018,** *33* (3), 595-603.

82. Li, J.; Ren, J.; Wang, W.; Wang, G.; Gu, G.; Wu, X.; Wang, Y.; Huang, M.; Li, J., Risk factors and clinical outcomes of hypervirulent *Klebsiella pneumoniae*  induced bloodstream infections. *Eur J Clin Microbiol Infect Dis* **2018,** *37* (4), 679-689.

83. Scheuerman, O.; Schechner, V.; Carmeli, Y.; Gutierrez-Gutierrez, B.; Calbo, E.; Almirante, B.; Viale, P. L.; Oliver, A.; Ruiz-Garbajosa, P.; Gasch, O.; Gozalo, M.; Pitout, J.; Akova, M.; Pena, C.; Molina, J.; Hernandez-Torres, A.; Venditti, M.; Prim, N.; Origuen, J.; Bou, G.; Tacconelli, E.; Tumbarello, M.; Hamprecht, A.; Karaiskos, I.; de la Calle, C.; Perez, F.; Schwaber, M. J.; Bermejo, J.; Lowman, W.; Hsueh, P. R.; Navarro-San Francisco, C.; Bonomo, R. A.; Paterson, D. L.; Pascual, A.; Rodriguez-Bano, J.; Reipi Esgbis, I., Comparison of Predictors and Mortality Between Bloodstream Infections Caused by ESBL-Producing Escherichia coli and ESBL-Producing *Klebsiella pneumoniae* . *Infection control and hospital epidemiology* **2018,** *39* (6), 660-667.

84. Shankar, C.; Nabarro, L. E.; Anandan, S.; Ravi, R.; Babu, P.; Munusamy, E.; Jeyaseelan, V.; Rupali, P.; Verghese, V. P.; Veeraraghavan, B., Extremely High Mortality Rates in Patients with Carbapenem-resistant, Hypermucoviscous *Klebsiella pneumoniae*  Blood Stream Infections. *The Journal of the Association of Physicians of India* **2018,** *66* (12), 13-16.

85. Xiao, T.; Yu, W.; Niu, T.; Huang, C.; Xiao, Y., A retrospective, comparative analysis of risk factors and outcomes in carbapenem-susceptible and carbapenem-nonsusceptible *Klebsiella pneumoniae*  bloodstream infections: tigecycline significantly increases the mortality. *Infection and drug resistance* **2018,** *11*, 595-606.

86. Xu, M.; Fu, Y.; Kong, H.; Chen, X.; Chen, Y.; Li, L.; Yang, Q., Bloodstream infections caused by *Klebsiella pneumoniae* : prevalence of bla(KPC), virulence factors and their impacts on clinical outcome. *BMC Infect Dis* **2018,** *18* (1), 358.

87. Yang, X.; Cui, J.; Zhao, J.; Ni, W., Clinical characteristics of carbapenem-resistant *Klebsiella pneumoniae*  bloodstream infection and the risk factors of mortality. *Chinese Journal of Infection and Chemotherapy* **2018,** *18* (2), 142-149.

88. Zhang, Y.; Guo, L. Y.; Song, W. Q.; Wang, Y.; Dong, F.; Liu, G., Risk factors for carbapenem-resistant K. pneumoniae bloodstream infection and predictors of mortality in Chinese paediatric patients. *BMC Infect Dis* **2018,** *18* (1), 248.

89. Zheng, S. H.; Cao, S. J.; Xu, H.; Feng, D.; Wan, L. P.; Wang, G. J.; Xiao, X. G., Risk factors, outcomes and genotypes of carbapenem-nonsusceptible *Klebsiella pneumoniae*  bloodstream infection: a three-year retrospective study in a large tertiary hospital in Northern China. *Infect Dis (Lond)* **2018,** *50* (6), 443-451.

90. Balkhair, A.; Al-Muharrmi, Z.; Al'Adawi, B.; Al Busaidi, I.; Taher, H. B.; Al-Siyabi, T.; Al Amin, M.; Hassan, K. S., Prevalence and 30-day all-cause mortality of carbapenem-and colistin-resistant bacteraemia caused by Acinetobacter baumannii, Pseudomonas aeruginosa, and *Klebsiella pneumoniae* : Description of a decade-long trend. *Int J Infect Dis* **2019,** *85*, 10-15.

91. Brescini, L.; Morroni, G.; Valeriani, C.; Castelletti, S.; Mingoia, M.; Simoni, S.; Masucci, A.; Montalti, R.; Vivarelli, M.; Giacometti, A.; Barchiesi, F., Clinical and epidemiological characteristics of KPC-producing *Klebsiella pneumoniae*  from bloodstream infections in a tertiary referral center in Italy. *BMC Infect Dis* **2019,** *19* (1), 611.

92. Imai, K.; Ishibashi, N.; Kodana, M.; Tarumoto, N.; Sakai, J.; Kawamura, T.; Takeuchi, S.; Taji, Y.; Ebihara, Y.; Ikebuchi, K.; Murakami, T.; Maeda, T.; Mitsutake, K.; Maesaki, S., Clinical characteristics in blood stream infections caused by *Klebsiella pneumoniae* , Klebsiella variicola, and Klebsiella quasipneumoniae: a comparative study, Japan, 2014-2017. *BMC Infect Dis* **2019,** *19* (1), 946.

93. Jahidul Hasan, M.; Rabbani, R.; Bachar, S. C., Evaluation of the therapeutic effectiveness of polymyxin B versus colistin in carbapenem-resistanct *Klebsiella pneumoniae* -associated bacteremia. *Dhaka University Journal of Pharmaceutical Sciences* **2019,** *18* (2), 209-215.

94. Juan, C. H.; Chuang, C.; Chen, C. H.; Li, L.; Lin, Y. T., Clinical characteristics, antimicrobial resistance and capsular types of community-acquired, healthcare-associated, and nosocomial *Klebsiella pneumoniae*  bacteremia. *Antimicrob Resist Infect Control* **2019,** *8*, 1.

95. Kim, D.; Park, B. Y.; Choi, M. H.; Yoon, E. J.; Lee, H.; Lee, K. J.; Park, Y. S.; Shin, J. H.; Uh, Y.; Shin, K. S.; Shin, J. H.; Kim, Y. A.; Jeong, S. H., Antimicrobial resistance and virulence factors of *Klebsiella pneumoniae*  affecting 30 day mortality in patients with bloodstream infection. *J Antimicrob Chemother* **2019,** *74* (1), 190-199.

96. Lee, J. S.; Choi, J. Y.; Chung, E. S.; Peck, K. R.; Ko, K. S., Variation in the formation of persister cells against meropenem in *Klebsiella pneumoniae*  bacteremia and analysis of its clinical features. *Diagnostic microbiology and infectious disease* **2019,** *95* (3), 114853.

97. Liu, J.; Wang, H.; Huang, Z.; Tao, X.; Li, J.; Hu, Y.; Dou, Q.; Zou, M., Risk factors and outcomes for carbapenem-resistant *Klebsiella pneumoniae*  bacteremia in onco-hematological patients. *Journal of infection in developing countries* **2019,** *13* (5), 357-364.

98. Liu, Q.; Wu, J.; Wang, Z.; Wu, X.; Wang, G.; Ren, J., Polymicrobial Bacteremia Involving *Klebsiella pneumoniae*  in Patients with Complicated Intra-Abdominal Infections: Frequency, Co-Pathogens, Risk Factors, and Clinical Outcomes. *Surg Infect (Larchmt)* **2019,** *20* (4), 317-325.

99. Medeiros, G. S.; Rigatto, M. H.; Falci, D. R.; Zavascki, A. P., Combination therapy with polymyxin B for carbapenemase-producing *Klebsiella pneumoniae*  bloodstream infection. *Int J Antimicrob Agents* **2019,** *53* (2), 152-157.

100. Menekşe; Çağ, Y.; Işık, M. E.; Şahin, S.; Hacıseyitoğlu, D.; Can, F.; Ergonul, O., The effect of colistin resistance and other predictors on fatality among patients with bloodstream infections due to *Klebsiella pneumoniae*  in an OXA-48 dominant region. *International Journal of Infectious Diseases* **2019,** *86*, 208-211.

101. Namikawa, H.; Niki, M.; Niki, M.; Yamada, K.; Nakaie, K.; Sakiyama, A.; Oinuma, K. I.; Tsubouchi, T.; Tochino, Y.; Takemoto, Y.; Kaneko, Y.; Shuto, T.; Kakeya, H., Clinical and virulence factors related to the 30-day mortality of *Klebsiella pneumoniae*  bacteremia at a tertiary hospital: a case-control study. *Eur J Clin Microbiol Infect Dis* **2019,** *38* (12), 2291-2297.

102. Reid, C. B.; Steele, L.; Pasquill, K.; Parfitt, E. C.; Laupland, K. B., Occurrence and determinants of Klebsiella species bloodstream infection in the western interior of British Columbia, Canada. *BMC Infect Dis* **2019,** *19* (1), 1070.

103. Sun, W. M.; Zhou, H.; Shen, L. S.; Yang, Q.; Ma, W. J.; Zhou, J. Y., The efficacy and safety of different antimicrobial regimens in carbapenem-resistant *Klebsiella pneumoniae*  bloodstream infections. *Zhonghua nei ke za zhi* **2019,** *58* (8), 566-571.

104. Surgers, L.; Boersma, P.; Girard, P. M.; Homor, A.; Geneste, D.; Arlet, G.; Decre, D.; Boyd, A., Molecular epidemiology of ESBL-producing E. coli and K. pneumoniae: establishing virulence clusters. *Infection and drug resistance* **2019,** *12*, 119-127.

105. Tan, T. Y.; Ong, M.; Cheng, Y.; Ng, L. S. Y., Hypermucoviscosity, rmpA, and aerobactin are associated with community-acquired *Klebsiella pneumoniae*  bacteremic isolates causing liver abscess in Singapore. *Journal of microbiology, immunology, and infection = Wei mian yu gan ran za zhi* **2019,** *52* (1), 30-34.

106. Chang, H.; Wei, J.; Zhou, W.; Yan, X.; Cao, X.; Zuo, L.; Chen, S.; Yao, K.; Huang, R.; Chen, Y.; Wu, C., Risk factors and mortality for patients with Bloodstream infections of *Klebsiella pneumoniae*  during 2014-2018: Clinical impact of carbapenem resistance in a large tertiary hospital of China. *J Infect Public Health* **2020,** *13* (5), 784-790.

107. Changpradub, D.; Prawang, A.; Santimaleeworagun, W.; Thunyaharn, S.; Puttilerpong, C., TREATMENT AND CLINICAL OUTCOME OF COLISTIN-RESISTANT *KLEBSIELLA PNEUMONIAE*  BACTEREMIA PATIENTS. *Southeast Asian Journal of Tropical Medicine and Public Health* **2020,** *51* (3), 263-269.

108. Chen, C. L.; Hou, P. C.; Wang, Y. T.; Lee, H. Y.; Zhou, Y. L.; Wu, T. S.; Janapatla, R. P.; Yang, C. C., The High mortality and antimicrobial resistance of *Klebsiella pneumoniae*  bacteremia in northern Taiwan. *Journal of infection in developing countries* **2020,** *14* (4), 373-379.

109. Cheng, J.; Zhang, G.; Li, Q.; Xu, H.; Yu, Q.; Yi, Q.; Luo, S.; Li, Y.; Tian, X.; Chen, D.; Luo, Z., Time to positivity of *Klebsiella pneumoniae*  in blood culture as prognostic indicator for pediatric bloodstream infections. *Eur J Pediatr* **2020,** *179* (11), 1689-1698.

110. Falcone, M.; Bassetti, M.; Tiseo, G.; Giordano, C.; Nencini, E.; Russo, A.; Graziano, E.; Tagliaferri, E.; Leonildi, A.; Barnini, S.; Farcomeni, A.; Menichetti, F., Time to appropriate antibiotic therapy is a predictor of outcome in patients with bloodstream infection caused by KPC-producing *Klebsiella pneumoniae* . *Critical care (London, England)* **2020,** *24* (1), 29.

111. Hong, K. W.; Cheon, Y. H.; Moon, K.; Hong, S. I.; Ryu, B. H.; Cho, O. H.; Bae, I. G., Comparison of the clinical characteristics and outcomes of bloodstream infections caused by Raoultella species and *Klebsiella pneumoniae* . *Infect Dis (Lond)* **2020,** *52* (7), 489-497.

112. Hong, M. Y.; Hsieh, C. C.; Yang, C. Y.; Lee, C. H.; Ko, W. C.; Lee, C. C., A Simple Scoring Algorithm That Predicts Abscesses in Adults with Community-Onset *Klebsiella pneumoniae*  Bacteremia: Hypermucoviscosity Matters. *Infection and drug resistance* **2020,** *13*, 1045-1055.

113. Huang, Y. T.; Chen, C. S.; Chen, H. A.; Hsu, H. S.; Liang, M. H.; Chang, M. H.; Liao, C. H., *Klebsiella pneumoniae*  bacteremia revisited: Comparison between 2007 and 2017 prospective cohorts at a medical center in Taiwan. *J Infect* **2020,** *81* (5), 753-757.

114. Lee, N. Y.; Tsai, C. S.; Syue, L. S.; Chen, P. L.; Li, C. W.; Li, M. C.; Ko, W. C., Treatment Outcome of Bacteremia Due to Non-Carbapenemase-producing Carbapenem-Resistant *Klebsiella pneumoniae*  Bacteremia: Role of Carbapenem Combination Therapy. *Clinical therapeutics* **2020,** *42* (3), e33-e44.

115. Li, S.; Liu, J.; Chen, F.; Cai, K.; Tan, J.; Xie, W.; Qian, R.; Liu, X.; Zhang, W.; Du, H.; Liu, Y.; Huang, L., A risk score based on pediatric sequential organ failure assessment predicts 90-day mortality in children with *Klebsiella pneumoniae*  bloodstream infection. *BMC Infect Dis* **2020,** *20* (1), 916.

116. Li, Y.; Li, J.; Hu, T.; Hu, J.; Song, N.; Zhang, Y.; Chen, Y., Five-year change of prevalence and risk factors for infection and mortality of carbapenem-resistant *Klebsiella pneumoniae*  bloodstream infection in a tertiary hospital in North China. *Antimicrob Resist Infect Control* **2020,** *9* (1), 79.

117. Nham, E.; Huh, K.; Cho, S. Y.; Chung, D. R.; Peck, K. R.; Lee, N. Y.; Kang, C. I., Characteristics and Clinical Outcomes of Extended-Spectrum beta-lactamase-producing *Klebsiella pneumoniae*  Bacteremia in Cancer Patients. *Infect Chemother* **2020,** *52* (1), 59-69.

118. Papadimitriou-Olivgeris, M.; Bartzavali, C.; Nikolopoulou, A.; Kolonitsiou, F.; Mplani, V.; Spiliopoulou, I.; Christofidou, M.; Fligou, F.; Marangos, M., Impact of Tigecycline's MIC in the Outcome of Critically Ill Patients with Carbapenemase-Producing *Klebsiella pneumoniae*  Bacteraemia Treated with Tigecycline Monotherapy-Validation of 2019's EUCAST Proposed Breakpoint Changes. *Antibiotics (Basel, Switzerland)* **2020,** *9* (11).

119. Shen, Y. F.; Chen, L. Y.; He, F., A STUDY ON CLINICAL OUTCOMES AND DEATH RISK FACTORS IN ADULT PATIENTS WITH *KLEBSIELLA PNEUMONIAE*  SEPTICEMIA. *Acta Medica Mediterranea* **2020,** *36* (5), 3167-3171.

120. Xiao, T.; Zhu, Y.; Zhang, S.; Wang, Y.; Shen, P.; Zhou, Y.; Yu, X.; Xiao, Y., A Retrospective Analysis of Risk Factors and Outcomes of Carbapenem-Resistant *Klebsiella pneumoniae*  Bacteremia in Nontransplant Patients. *J Infect Dis* **2020,** *221* (Suppl 2), S174-s183.

121. Yuan, Y.; Wang, J.; Yao, Z.; Ma, B.; Li, Y.; Yan, W.; Wang, S.; Ma, Q.; Zhang, J.; Xu, J.; Li, L.; Wang, Y.; Fan, E., Risk Factors for Carbapenem-Resistant *Klebsiella pneumoniae*  Bloodstream Infections and Outcomes. *Infection and drug resistance* **2020,** *13*, 207-215.

122. Zhang, G.; Zhang, M.; Sun, F.; Zhou, J.; Wang, Y.; Zhu, D.; Chen, Z.; Chen, Q.; Chang, Q.; Liu, H.; Chai, W.; Pan, H., Epidemiology, mortality and risk factors for patients with K. pneumoniae bloodstream infections: Clinical impact of carbapenem resistance in a tertiary university teaching hospital of Beijing. *J Infect Public Health* **2020,** *13* (11), 1710-1714.

123. Zhang, S.; Yang, Z.; Sun, L.; Wang, Z.; Sun, L.; Xu, J.; Zeng, L.; Sun, T., Clinical Observation and Prognostic Analysis of Patients With *Klebsiella pneumoniae*  Bloodstream Infection. *Front Cell Infect Microbiol* **2020,** *10*, 577244.

124. Balkan, II; Alkan, M.; Aygun, G.; Kuskucu, M.; Ankarali, H.; Karagoz, A.; Sen, S.; Arsu, H. Y.; Bicer, M.; Kaya, S. Y.; Karaali, R.; Mete, B.; Saltoglu, N.; Tabak, F., Colistin resistance increases 28-day mortality in bloodstream infections due to carbapenem-resistant *Klebsiella pneumoniae* . *European Journal of Clinical Microbiology & Infectious Diseases* **2021,** *40* (10), 2161-2170.

125. Eren, E.; Ulu-Kılıç, A.; Türe, Z.; Cevahir, F.; Kılıç, H.; Alp-Meşe, E., Risk factors of mortality in patients with bloodstream infections due to carbapenem resistant klebsiella pneumonia. *Klimik Dergisi* **2021,** *34* (1), 56-60.

126. Hsu, J. Y.; Chuang, Y. C.; Wang, J. T.; Chen, Y. C.; Hsieh, S. M., Healthcare-associated carbapenem-resistant *Klebsiella pneumoniae*  bloodstream infections: Risk factors, mortality, and antimicrobial susceptibility, 2017-2019. *Journal of the Formosan Medical Association = Taiwan yi zhi* **2021,** *120* (11), 1994-2002.

127. Lee, I. R.; Thein, T. L.; Ang, L.; Ding, Y.; Lim, J. J.; Bok, C. F.; Mukherjee, S.; Titin, C.; Kalimuddin, S.; Archuleta, S.; Lye, D. C., Cefazolin versus ceftriaxone as definitive treatment for *Klebsiella pneumoniae*  bacteraemia: a retrospective multicentre study in Singapore. *Journal of Antimicrobial Chemotherapy* **2021,** *76* (5), 1303-1310.

128. Lee, N. Y.; Lo, C. L.; Chen, P. L.; Syue, L. S.; Li, C. W.; Li, M. C.; Ko, W. C., Clinical impact of cefepime breakpoint in patients with carbapenem-resistant *Klebsiella pneumoniae*  bacteraemia. *Int J Antimicrob Agents* **2021,** *57* (2), 106250.

129. Liu, K. S.; Tong, Y. S.; Lee, M. T.; Lin, H. Y.; Lu, M. C., Risk Factors of 30-Day All-Cause Mortality in Patients with Carbapenem-Resistant *Klebsiella pneumoniae*  Bloodstream Infection. *J Pers Med* **2021,** *11* (7).

130. Man, M. Y.; Shum, H. P.; Li, K. C.; Yan, W. W., Impact of appropriate empirical antibiotics on clinical outcomes in *Klebsiella pneumoniae*  bacteraemia. *Hong Kong medical journal = Xianggang yi xue za zhi* **2021,** *27* (4), 247-257.

131. Papadimitriou-Olivgeris, M.; Bartzavali, C.; Georgakopoulou, A.; Kolonitsiou, F.; Mplani, V.; Spiliopoulou, I.; Christofidou, M.; Fligou, F.; Marangos, M., External validation of INCREMENT-CPE score in a retrospective cohort of carbapenemase-producing *Klebsiella pneumoniae*  bloodstream infections in critically ill patients. *Clin Microbiol Infect* **2021,** *27* (6), 915.e1-915.e3.

132. Papadimitriou-Olivgeris, M.; Bartzavali, C.; Georgakopoulou, A.; Kolonitsiou, F.; Papamichail, C.; Spiliopoulou, I.; Christofidou, M.; Fligou, F.; Marangos, M., Mortality of Pandrug-Resistant *Klebsiella pneumoniae*  Bloodstream Infections in Critically Ill Patients: A Retrospective Cohort of 115 Episodes. *Antibiotics (Basel, Switzerland)* **2021,** *10* (1).

133. Perez, F.; Colindres, R. V.; Wilson, B. M.; Saade, E.; Jump, R. L. P.; Banerjee, R.; Patel, R.; Evans, S. R.; Bonomo, R. A., Desirability of Outcome Ranking for the Management of Antimicrobial Therapy (DOOR MAT) Reveals Improvements in the Treatment of Bloodstream Infections Caused by Escherichia coli and *Klebsiella pneumoniae*  in Patients from the Veterans Health Administration. *Clin Infect Dis* **2021,** *73* (7), 1231-1238.

134. Rodriguez, O. L.; Sousa, A.; Perez-Rodriguez, M. T.; Martinez-Lamas, L.; Suarez, R. L.; Martinez, C. T.; Pino, C. P.; Vidal, F. V.; Perez-Landeiro, A.; Casal, M. C., Mortality-related factors in patients with OXA-48 carbapenemase-producing *Klebsiella pneumoniae*  bacteremia. *Medicine* **2021,** *100* (14).

135. Shen, L.; Lian, C.; Zhu, B.; Yao, Y.; Yang, Q.; Zhou, J.; Zhou, H., Bloodstream Infections due to Carbapenem-Resistant *Klebsiella pneumoniae* : A Single-Center Retrospective Study on Risk Factors and Therapy Options. *Microb Drug Resist* **2021,** *27* (2), 227-233.

136. Song, F.; Zhang, K.; Huang, J.; Qian, Z.; Zhou, H.; Cai, J.; Zheng, C.; Zhou, F.; Cui, W.; Zhang, G., Clinical Characteristics, Risk Factors, and Outcomes of Patients with Polymicrobial *Klebsiella pneumoniae*  Bloodstream Infections. *BioMed research international* **2021,** *2021*, 6619911.

137. Sung, H. S.; Lee, J. W.; Bae, S.; Kwon, K. T., Comparison of antimicrobial resistances and clinical features in community-onset Escherichia coli and *Klebsiella pneumoniae*  bacteremia. *The Korean journal of internal medicine* **2021,** *36* (2), 433-440.

138. Tsai, W. C.; Syue, L. S.; Ko, W. C.; Lo, C. L.; Lee, N. Y., Antimicrobial treatment of monomicrobial phenotypic carbapenem-resistant *Klebsiella pneumoniae*  bacteremia: Two are better than one. *Journal of microbiology, immunology, and infection = Wei mian yu gan ran za zhi* **2021**.

139. Wu, X.; Shi, Q.; Shen, S.; Huang, C.; Wu, H., Clinical and Bacterial Characteristics of *Klebsiella pneumoniae*  Affecting 30-Day Mortality in Patients With Bloodstream Infection. *Front Cell Infect Microbiol* **2021,** *11*, 688989.

140. Ang, S. H.; Petrick, P.; Shamsul, A. S.; Ramliza, R.; Kori, N.; Lau, C. L., The risk factors for complications and survival outcomes of *Klebsiella pneumoniae*  Bacteraemia in Hospital Canselor Tuanku Muhriz Universiti Kebangsaan Malaysia. *The Medical journal of Malaysia* **2022,** *77* (4), 440-445.

141. Aslan, A. T.; Kırbaş, E.; Sancak, B.; Tanrıverdi, E. S.; Otlu, B.; Gürsoy, N. C.; Yılmaz, Y. A.; Tozluyurt, A.; Liste, Ü.; Bıçakcıgil, A.; Hazırolan, G.; Dağ, O.; Güven, G. S.; Akova, M., A retrospective observational cohort study of the clinical epidemiology of bloodstream infections due to carbapenem-resistant *Klebsiella pneumoniae*  in an OXA-48 endemic setting. *Int J Antimicrob Agents* **2022,** *59* (4), 106554.

142. Atalay, E.; Bi̇Rengel, M. S.; Çinar, G.; Balik, İ., Investigation of the Effect of Double Carbapenem Therapy on Clinical Outcomes in Patients with *Klebsiella pneumoniae*  Bacteremia. *Flora* **2022,** *27* (2), 296-304.

143. Cao, Z. B.; Yue, C. C.; Kong, Q. X.; Liu, Y. Y.; Li, J. B., Risk Factors for a Hospital-Acquired Carbapenem-Resistant *Klebsiella pneumoniae*  Bloodstream Infection: A Five-Year Retrospective Study. *Infection and drug resistance* **2022,** *15*, 641-654.

144. Chen, Y.; Chen, Y.; Liu, P.; Guo, P.; Wu, Z.; Peng, Y.; Deng, J.; Kong, Y.; Cui, Y.; Liao, K.; Huang, B., Risk factors and mortality for elderly patients with bloodstream infection of carbapenem resistance *Klebsiella pneumoniae* : a 10-year longitudinal study. *BMC geriatrics* **2022,** *22* (1), 573.

145. Chen, Y.; Ying, S.; Qiu, Y.; Jiang, L.; Dong, S.; Dai, J.; Jin, X.; Yu, W., A Novel Nomogram for Predicting Risk Factors and Outcomes in Bloodstream Infections Caused by *Klebsiella pneumoniae* . *Infection and drug resistance* **2022,** *15*, 1317-1328.

146. Liang, X.; Chen, P.; Deng, B.; Sun, F. H.; Yang, Y.; Yang, Y.; He, R.; Qin, M.; Wu, Y.; Yang, F.; Tian, G. B.; Dai, M., Outcomes and Risk Factors of Bloodstream Infections Caused by Carbapenem-Resistant and Non-Carbapenem-Resistant *Klebsiella pneumoniae*  in China. *Infection and drug resistance* **2022,** *15*, 3161-3171.

147. Lima, O.; Sousa, A.; Longueira-Suarez, R.; Filgueira, A.; Taboada-Martinez, C.; Portela-Pino, C.; Nodar, A.; Vasallo-Vidal, F.; Martinez-Lamas, L.; Perez-Landeiro, A.; Rubianes, M.; Perez-Rodriguez, M. T., Ceftazidime-avibactam treatment in bacteremia caused by OXA-48 carbapenemase-producing *Klebsiella pneumoniae* . *European Journal of Clinical Microbiology & Infectious Diseases* **2022,** *41* (9), 1173-1182.

148. Liu, C.; Liu, L.; Jin, M. M.; Hu, Y. B.; Cai, X.; Wan, L.; Zhang, H. Y.; Li, R. Y.; Wu, X. J., Molecular Epidemiology and Risk Factors of Carbapenem-Resistant *Klebsiella pneumoniae*  Bloodstream Infections in Wuhan, China. *Curr Med Sci* **2022,** *42* (1), 68-76.

149. Mairi, A.; Meyer, S.; Tilloy, V.; Barraud, O.; Touati, A., Whole Genome Sequencing of Extended-Spectrum Beta-Lactamase-Producing *Klebsiella pneumoniae*  Isolated from Neonatal Bloodstream Infections at a Neonatal Care Unit, Algeria. *Microbial drug resistance (Larchmont, N.Y.)* **2022**.

150. Meng, H.; Han, L.; Niu, M.; Xu, L.; Xu, M.; An, Q.; Lu, J., Risk Factors for Mortality and Outcomes in Hematological Malignancy Patients with Carbapenem-Resistant *Klebsiella pneumoniae*  Bloodstream Infections. *and Drug Resistance* **2022,** *15*, 4241-4251.

151. Onorato, L.; Sarnelli, B.; D’agostino, F.; Signoriello, G.; Trama, U.; D’argenzio, A.; Montemurro, M. V.; Coppola, N., Epidemiological, Clinical and Microbiological Characteristics of Patients with Bloodstream Infections Due to Carbapenem-Resistant K. Pneumoniae in Southern Italy: A Multicentre Study. *Antibiotics* **2022,** *11* (5).

152. Sheng, Z.; Li, J.; Chen, T.; Zhu, Y.; Yu, X.; He, X.; Zheng, Y.; Ma, C.; Zheng, M.; Wang, P.; Li, Z.; Xu, Y.; Xie, Q.; Su, Z.; Chen, S., Clinical and Microbiological Characteristics of *Klebsiella pneumoniae*  Bloodstream Infection in a Chinese Hospital: Hypervirulent and Multiclonal. *Infection and drug resistance* **2022,** *15*, 3981-3990.

153. Soares de Moraes, L.; Gomes Magalhaes, G. L.; Material Soncini, J. G.; Pelisson, M.; Eches Perugini, M. R.; Vespero, E. C., High mortality from carbapenem-resistant *Klebsiella pneumoniae*  bloodstream infection. *Microb Pathog* **2022,** *167*, 105519.

154. Wu, H.; Mao, Y.; Du, X.; Zhao, F.; Jiang, Y.; Yu, Y., The Value of Neutrophil-To-Lymphocyte Ratio for Evaluating Blood Stream Infection Caused by Carbapenem-Resistant *Klebsiella pneumoniae* : A Retrospective Cohort Study. *Frontiers in Medicine* **2022,** *9*.

155. Ying, P.; Chen, J.; Ye, Y.; Ye, J.; Cai, W., Adipose tissue is a predictor of 30-days mortality in patients with bloodstream infection caused by carbapenem-resistant *Klebsiella pneumoniae* . *BMC Infect Dis* **2022,** *22* (1), 173.

156. Lee, J. A.; Kang, C. I.; Joo, E. J.; Ha, Y. E.; Park, S. Y.; Chung, D. R.; Peck, K. R.; Lee, N. Y.; Song, J. H., Clinical and microbiological characteristics of healthcare-associated infections in community-onset *Klebsiella pneumoniae*  bacteremia. *Infection and Chemotherapy* **2012,** *44* (2), 56-61.

157. Zhang, Y.; Xu, Y.; Huang, Y., Virulence Genotype and Correlation of Clinical Severeness with Presence of the Type VI Secretion System in *Klebsiella pneumoniae*  Isolates Causing Bloodstream Infections. *Infection and drug resistance* **2022,** *15*, 1487-1497.
